# Community-facility linkage models and maternal and infant health outcomes in Malawi’s PMTCT/ART program: A cohort study

**DOI:** 10.1371/journal.pmed.1003780

**Published:** 2021-09-17

**Authors:** Michael E. Herce, Maganizo B. Chagomerana, Lauren C. Zalla, Nicole B. Carbone, Benjamin H. Chi, Michael T. Eliya, Sam Phiri, Stephanie M. Topp, Maria H. Kim, Emily B. Wroe, Chileshe Chilangwa, Jacqueline Chinkonde, Innocent A. Mofolo, Mina C. Hosseinipour, Jessie K. Edwards

**Affiliations:** 1 University of North Carolina Project/Malawi, Lilongwe, Malawi; 2 Institute for Global Health & Infectious Diseases, University of North Carolina School of Medicine, Chapel Hill, North Carolina, United States of America; 3 Department of Epidemiology, Gillings School of Global Public Health, University of North Carolina at Chapel Hill, Chapel Hill, North Carolina, United States of America; 4 Division of Global Women’s Health, Department of Obstetrics & Gynecology, School of Medicine, University of North Carolina at Chapel Hill, Chapel Hill, North Carolina, United States of America; 5 Department of HIV and AIDS, Ministry of Health, Government of the Republic of Malawi, Lilongwe, Malawi; 6 Lighthouse Trust, Lilongwe, Malawi; 7 College of Public Health, Medical and Veterinary Sciences, James Cook University, Queensland, Australia; 8 Baylor International Pediatrics AIDS Initiative, Texas Children’s Hospital, Houston, Texas, United States of America; 9 Department of Pediatrics, Baylor College of Medicine, Houston, Texas, United States of America; 10 Division of Global Health Equity, Department of Medicine, Brigham & Women’s Hospital, Boston, Massachusetts, United States of America; 11 Abwenzi Pa Za Umoyo/Partners In Health—Malawi, Neno, Malawi; 12 Mothers2Mothers/Malawi, Lilongwe, Malawi; 13 UNICEF/Malawi, Lilongwe, Malawi; University of Southampton, UNITED KINGDOM

## Abstract

**Background:**

In sub-Saharan Africa, 3 community-facility linkage (CFL) models—Expert Clients, Community Health Workers (CHWs), and Mentor Mothers—have been widely implemented to support pregnant and breastfeeding women (PBFW) living with HIV and their infants to access and sustain care for prevention of mother-to-child transmission of HIV (PMTCT), yet their comparative impact under real-world conditions is poorly understood.

**Methods and findings:**

We sought to estimate the effects of CFL models on a primary outcome of maternal loss to follow-up (LTFU), and secondary outcomes of maternal longitudinal viral suppression and infant “poor outcome” (encompassing documented HIV-positive test result, LTFU, or death), in Malawi’s PMTCT/ART program. We sampled 30 of 42 high-volume health facilities (“sites”) in 5 Malawi districts for study inclusion. At each site, we reviewed medical records for all newly HIV-diagnosed PBFW entering the PMTCT program between July 1, 2016 and June 30, 2017, and, for pregnancies resulting in live births, their HIV-exposed infants, yielding 2,589 potentially eligible mother–infant pairs. Of these, 2,049 (79.1%) had an available HIV treatment record and formed the study cohort. A randomly selected subset of 817 (40.0%) cohort members underwent a field survey, consisting of a questionnaire and HIV biomarker assessment. Survey responses and biomarker results were used to impute CFL model exposure, maternal viral load, and early infant diagnosis (EID) outcomes for those missing these measures to enrich data in the larger cohort. We applied sampling weights in all statistical analyses to account for the differing proportions of facilities sampled by district. Of the 2,049 mother–infant pairs analyzed, 62.2% enrolled in PMTCT at a primary health center, at which time 43.7% of PBFW were ≤24 years old, and 778 (38.0%) received the Expert Client model, 640 (31.2%) the CHW model, 345 (16.8%) the Mentor Mother model, 192 (9.4%) ≥2 models, and 94 (4.6%) no model. Maternal LTFU varied by model, with LTFU being more likely among Mentor Mother model recipients (adjusted hazard ratio [aHR]: 1.45; 95% confidence interval [CI]: 1.14, 1.84; *p* = 0.003) than Expert Client recipients. Over 2 years from HIV diagnosis, PBFW supported by CHWs spent 14.3% (95% CI: 2.6%, 26.1%; *p* = 0.02) more days in an optimal state of antiretroviral therapy (ART) retention with viral suppression than women supported by Expert Clients. Infants receiving the Mentor Mother model (aHR: 1.24, 95% CI: 1.01, 1.52; *p* = 0.04) and ≥2 models (aHR: 1.44, 95% CI: 1.20, 1.74; *p* < 0.001) were more likely to undergo EID testing by age 6 months than infants supported by Expert Clients. Infants receiving the CHW and Mentor Mother models were 1.15 (95% CI: 0.80, 1.67; *p* = 0.44) and 0.84 (95% CI: 0.50, 1.42; *p* = 0.51) times as likely, respectively, to experience a poor outcome by 1 year than those supported by Expert Clients, but not significantly so. Study limitations include possible residual confounding, which may lead to inaccurate conclusions about the impacts of CFL models, uncertain generalizability of findings to other settings, and missing infant medical record data that limited the precision of infant outcome measurement.

**Conclusions:**

In this descriptive study, we observed widespread reach of CFL models in Malawi, with favorable maternal outcomes in the CHW model and greater infant EID testing uptake in the Mentor Mother model. Our findings point to important differences in maternal and infant HIV outcomes by CFL model along the PMTCT continuum and suggest future opportunities to identify key features of CFL models driving these outcome differences.

## Abstract 2

Michael Herce and co-workers study potential improvements in linkage to care for prevention of mother to child transmission of HIV in Malawi.

## Author summary

**Why was this study done?:**

- Attrition from the prevention of mother-to-child transmission of HIV (PMTCT) continuum threatens health gains for pregnant and breastfeeding women (PBFW) living with HIV and their infants.
- To improve PMTCT services in Africa, 3 community-facility linkage (CFL) models—Mentor Mothers, Community Health Workers (CHWs), and Expert Clients/Patients—have been widely implemented. Available evidence suggests that these CFL models may improve the health of PBFW living with HIV and their infants.
- Despite the promise of CFL models, however, no studies from the current “treat all” era have attempted to examine which of these models works best in “real world” PMTCT programs for ensuring maternal HIV care retention and viral suppression and infant HIV-free survival.

**What did the researchers do and find?:**

- We conducted a descriptive study in Malawi by sampling busy health facilities in 5 districts and, at selected facilities, reviewing medical records for all newly HIV-diagnosed PBFW and their HIV-exposed infants. We then administered a survey to a randomly selected subset of these mother–infant pairs to assess CFL model exposure and HIV outcomes.
- We found that compared to women supported by the most common CFL model, Expert Clients, women supported by CHWs were 13% more likely to start antiretroviral therapy (ART) soon after their HIV diagnosis. Over the 2 years following HIV diagnosis, PBFW living with HIV who received CHW model support spent 14% more time retained on ART and virally suppressed than women in the Expert Client model.
- Infants receiving Mentor Mother model support were 24% more likely to receive HIV testing by age 6 months than infants supported by Expert Clients. The 12-month infant risk of experiencing a positive HIV test result, loss to follow-up, or death did not vary significantly by CFL model.

**What do these findings mean?:**

- CFL models had widespread coverage in the national PMTCT/ART program in our selected sites in Malawi, reaching most mother–infant pairs.
- Important differences were noted in maternal and infant HIV outcomes by CFL model. Favorable HIV outcomes were seen in women supported by the CHW model, and higher infant EID testing uptake was observed in infants supported by Mentor Mothers.
- Although caution should be exercised when applying our findings to other settings, we found that CFL models may have different impacts on maternal and infant HIV outcomes at different steps along the PMTCT continuum in Malawi. Future research should identify the key features of CFL models that drive these outcome differences to better inform PMTCT programming in Malawi.

## Introduction

Universal test and treat for pregnant and breastfeeding women (PBFW) living with HIV, first introduced as the “Option B+” strategy, has saved countless maternal lives and averted thousands of pediatric HIV infections throughout sub-Saharan Africa [1–4]. Despite dramatic expansion of prevention of mother-to-child transmission of HIV (PMTCT) services and coverage with Option B+ [5], recent progress has been threatened by client attrition from the PMTCT continuum. For example, in Malawi, as many as 30% of pregnant women living with HIV become lost to follow-up within 24 months of antiretroviral therapy (ART) initiation, and 14% of infants exposed to HIV fall out of care by 12 months of age [6]. Such follow-up losses can have deleterious downstream health impacts for mothers and infants [7] and undermine progress toward the elimination of mother-to-child transmission of HIV in pursuit of international targets [8,9].

Multiple well-documented psychosocial, health system, and structural barriers contribute to client attrition along the PMTCT continuum [10–16]. Several strategies seek to address these obstacles by strengthening community-facility linkage (CFL)—i.e., the “formalized connection between a health facility and the communities it serves to support improved health outcomes” [17]. Similar to other HIV high-burden countries in sub-Saharan Africa, 3 prominent CFL models have been implemented in Malawi to support PMTCT services: Mentor Mothers, Community Health Workers (CHWs), and Expert Clients (or Patients) [18]. These interventions all aim to enhance routine, facility-based PMTCT services by mobilizing peers or other lay health providers to deliver health education, provide psychosocial support for ART and clinic appointment adherence, and trace mother–infant pairs when they fall out of PMTCT care [17]. More specifically, Mentor Mothers and Expert Clients are typically peer providers living with HIV who offer longitudinal health education and psychosocial support to PMTCT clients by drawing upon their own lived experiences with HIV [19]. CHWs are usually lay health workers who provide clients with psychosocial support, promote medication and appointment attendance, accompany clients to health facilities for clinic visits, and extend the services provided by other, often facility-based, health workers [19,20].

Evidence from sub-Saharan Africa suggests that these and other CFL models may have beneficial effects on a range of outcomes for persons living with HIV [20–25], including for mother–infant pairs [20,26]. Reports from Rwanda and Malawi indicate that nongovernmental organization (NGO)-supported CHWs catalyze improvements in ART uptake, infant PMTCT prophylaxis, and early infant diagnosis (EID) of HIV testing [21,27]. Similarly, Mentor Mothers have been shown to improve 12-month ART retention for clients receiving PMTCT services in Uganda [28,29], and Expert Clients, a somewhat newer CFL provider cadre, have been noted to support several activities ranging from community-based support groups to facility-based counseling and vital sign measurement [30]. A recent cluster randomized controlled trial from Malawi, the PURE trial, suggests that peer support provided in the community (equivalent to the Expert Client model) or the facility (akin to the Mentor Mother model) increases maternal ART uptake and PMTCT retention [25].

Despite the promise of these CFL models, no published reports examine their comparative impact in routine PMTCT programs in the current “treat all” era [26,31]. This represents a critical evidence gap because, although these interventions share a common approach, they often differ in how they deliver psychosocial support to women and their newborns in practice, with implications for which model(s) may work best in “real world” programmatic settings. To address this gap, we conducted a descriptive study to estimate the comparative effects of Mentor Mother, CHW, and Expert Client models on maternal and infant health outcomes in the integrated national PMTCT/ART program in Malawi. In this paper, we present our main findings and report them according to established criteria for observational studies [32].

## Methods

### Ethics statement

The study was approved by the Malawi National Health Sciences Research Committee (#17/05/1812), and the institutional review boards of the University of North Carolina at Chapel Hill, United States of America (#17–1114), Brigham & Women’s Hospital, USA (Reliance Agreement #17–1114), and James Cook University, Australia (HREC #1812). All regulatory bodies approved the study protocol (**S1 Protocol**) and related documents, including exemption of individual informed consent for the review of existing deidentified, routinely collected data.

### Study design and setting overview

We employed an observational cohort study design, enriched by a field survey, to evaluate the effectiveness of CFL models for improving PMTCT outcomes in high-volume health facilities in 5 districts of Malawi (**S1 STROBE Checklist**). Specifically, we examined the impact of 3 CFL models on accelerating ART initiation, reducing loss to follow-up (LTFU), and improving viral suppression among women diagnosed with HIV during pregnancy or breastfeeding. We also examined the impacts of these interventions on outcomes among infants exposed to HIV, including uptake of EID testing and infant LTFU over the first year of life. For the study, we purposively selected 5 districts—Lilongwe, Salima, Zomba, and Mzimba North and Mzimba South (referred to collectively here as “Mzimba”)—that together span 4 of 5 health zones nationally; include diverse urban, peri-urban, and rural catchment areas; and capture the 3 CFL models of interest.

### Community-facility linkage models

During the study period, 3 main CFL models—Mentor Mothers, CHWs, and Expert Clients—provided support to PMTCT clients in study districts. To varying degrees, all 3 models offered PMTCT clients health education, ART adherence support, and “back to care” services for women and infants who disengaged from care, among other services. Data from a CFL model survey (**S1 Appendix**) conducted with 15 CFL model program managers and Ministry of Health (MOH) healthcare supervisors in the 5 study districts provide a summary overview of major CFL model activities and characteristics (**S1 Table**). Donor-supported NGOs implemented the Mentor Mother (i.e., mothers2mothers) and CHW (i.e., the Tingathe program of the Baylor College of Medicine Children’s Foundation) models, whereas a mix of NGOs (e.g., Elizabeth Glaser Pediatric AIDS Foundation) and the Malawi MOH delivered the Expert Client model, depending on the site. All models had been in operation at the study sites for at least several months and, in most cases, a few years, during the study period. The MOH—together with donors, NGOs, and other implementing partners—determined the allocation of CFL models to study districts and individual health facilities based on public health programming priorities. In most cases, this meant that 1 CFL model was operating per site during the period of interest; however, in a few cases, particularly at the largest urban health facilities in Lilongwe district, this opened the possibility of 2 or more models concurrently operating at the same site. Additional details on the programming, policy, and implementation landscape for the CFL models studied here are described elsewhere [33,34].

### Malawi’s national PMTCT/ART program

The establishment and evolution of Malawi’s national PMTCT program have been well described [3,35]. Briefly, in 2002, Malawi began offering PMTCT services as part of its national HIV program, which initially included HIV testing and counseling and single-dose nevirapine. Over a period of scale-up from 2004 to 2010, the PMTCT program expanded to 454 antenatal clinics nationally offering free integrated HIV testing services, maternal combination antiretroviral prophylaxis, and referral to, and limited on-site provision of, ART services for women meeting criteria for treatment for their own health [35]. In 2011, Malawi introduced the Option B+ strategy, becoming the first country globally to offer rapid, universal, and lifelong ART to PBFW living with HIV regardless of CD4 count or stage of clinical disease. To implement Option B+, Malawi recognized and acted on the need to fully integrate its PMTCT and ART programs at all levels, including for supply chain management, health worker training and supervision, longitudinal cohort reporting of client outcomes, and decentralization of services to all facilities offering antenatal care [35]. The Option B+ experience proved foundational for the national transition to a treat all policy, which was undertaken by the Malawi MOH in mid-2016 and codified in recent national HIV management guidelines [36,37]. Under Malawi’s current integrated PMTCT/ ART program, ART for pregnant women living with HIV may be started and continued within antenatal (i.e., “ANC”)/PMTCT clinics, maternity departments, and/or ART clinics. Following delivery, follow-up for lifelong maternal ART and infant HIV exposure (including EID) is typically delivered in the same HIV care setting (usually the ART clinic) using synchronized appointments [6,38]. Despite programmatic integration, variability has been reported in how facilities organize their PMTCT/ART services, with some using referral systems between the antenatal and ART clinics [38].

### Study population

The target population for this study comprised all PBFW living with HIV, and their infants, who were referred to, or newly enrolled in, PMTCT services at a high-volume health facility during introduction of national treat all policy in our 5 study districts. To reach this target population, we used electronic MOH-integrated HIV program data to identify high-volume health facilities and surrounding catchment areas (collectively referred to as “sites”) in each district. We designated a site as “high-volume” if it reported ≥30 PBFW newly diagnosed with HIV in the year prior to treat all introduction, which generally included sites in the 50th percentile and higher for new PMTCT client volumes in the district. These high-volume sites were prioritized since they were the most likely to offer CFL model services. We excluded high-volume sites serving highly transient catchment areas (reflected by high rates of “transfer out” from the PMTCT program) that would severely limit outcome ascertainment in the routine record. We visited all eligible high-volume sites in 4 of the 5 study districts (i.e., 22 total) and a 50% random sample of eligible sites (i.e., 8 of 16) in the last study district due to operational constraints.

At each site, we constructed a study cohort of mother–infant pairs by collecting routine medical record data on all HIV-diagnosed PBFW meeting study eligibility criteria, and, for pregnancies that resulted in live births, their infant’s medical record data. Mother–infant pairs were included in the study cohort if the mother met the following criteria: newly diagnosed or had documented evidence of recent (i.e., within 90 days) HIV infection at any time between July 1, 2016 and June 30, 2017; pregnant or breastfeeding at the time of HIV diagnosis; documented enrollment in, or referral to (i.e., found to have new or recent HIV infection but not linked to care), ART services within Malawi’s PMTCT program; ≥16 years of age at time of PMTCT enrollment; and received any antenatal, maternity, or PMTCT/ART services at a study site. Because most outcomes (e.g., maternal LTFU) were measured using the MOH HIV treatment card, we excluded any pair in which the mother had a missing HIV treatment card.

Next, we invited a random subset of mother–infant pairs that we sampled from the study cohort to complete a 1-time field survey. The field survey collected biomarker and questionnaire data to overcome known limitations with the routine medical record (e.g., missing data), and, thus, enable detailed ascertainment of CFL model exposure, maternal viral load (VL), and infant HIV status. All mother–infant pairs in the study cohort (regardless of whether they ever established care or started ART or were categorized as LTFU in the PMTCT/ART program) were eligible to be sampled for inclusion in the field survey. Our sampling approach is presented in the supporting information (**S2 Table**). Briefly, we sampled a large proportion of the study cohort for the field survey, including 100% of mother–infant pairs at 28 of 30 eligible high-volume sites, due to concerns about high rates of survey nonresponse and operational challenges with conducting community tracing and field survey activities across multiple sites (**S2 Table**). Survey data enriched data for the larger study cohort and were used specifically to (1) impute CFL model exposure among mother–infant pairs; (2) improve imputation of maternal VL among women missing VL data in the routine record; and (3) augment infant HIV testing data.

### Outcomes

Our primary outcome of interest was maternal retention in care in the study cohort, which we measured as LTFU over the PMTCT continuum in order to evaluate all maternal and infant outcomes on the same scale (i.e., with event probabilities increasing over time, rather than decreasing). We defined LTFU according to the MOH definition using dates reported by each facility [36,37]. With this rationale, we described the cumulative incidence of maternal LTFU from study sites at 2 years post-HIV diagnosis among all eligible women. While the primary analysis examined LTFU from study sites, we were also interested in using this outcome as a proxy for loss to care entirely. LTFU from study sites might not correspond to loss to clinical care because some “silent” transfers may not have been documented in the facility records [39]. Thus, we examined the prevalence of such silent transfers using data from the field survey. Other secondary maternal outcomes included the following: the cumulative proportion who initiated ART by 6 months after HIV diagnosis; median time from HIV diagnosis to ART initiation; and proportion having a HIV-1 VL <1,000 copies/ml (i.e., consistent with programmatic definitions of “viral suppression” [40,41]). Since maximizing cumulative time spent retained on ART with viral suppression is the ultimate goal for women living with HIV in any PMTCT program, we also developed a post hoc “longitudinal maternal suppression” metric to estimate the amount of time women stayed in this final, targeted state within the PMTCT continuum.

Secondary outcomes also included the following indicators for newborns exposed to HIV: EID testing uptake (requiring documentation of a HIV-1 DNA PCR test result) from age 6 weeks; infant LTFU, defined according to the MOH definition in the routine clinic record [36,37]; and 12-month in care survival. Because relatively few infant deaths and vertical transmission events were observed, we created a post hoc infant “poor outcome” (i.e., a composite outcome encompassing a documented positive HIV test result, LTFU, or death by 1 year of age) to summarize clinical events of interest among infants exposed to HIV.

### Study procedures, data collection, and data management

Data collection took place between January 15, 2018 and May 31, 2019, and proceeded in 2 steps. In the first step, we reviewed all routinely available MOH medical record data on each woman and her infant in the study cohort from maternal HIV diagnosis through the child’s second birthday. Sources for medical record information included maternal and pediatric HIV treatment cards, the national Baobob electronic medical record, ART registers, infant follow-up cards and registers, maternity registers, and HIV VL and EID HIV-1 DNA PCR testing registers. The MOH Department of HIV and AIDS curated these routine data sources throughout the study period and ensured data quality through a robust supportive supervision protocol implemented quarterly since 2004 to collect and verify site-level data [6]. NGO partners operating in each district supported the MOH Department of HIV and AIDS protocol through monitoring and evaluation activities designed to promote routine data accuracy and completeness for their respective program reporting purposes. From the aforementioned routine information sources, we abstracted data on key variables necessary to reconstruct the PMTCT continuum for all mother–infant pairs in the study cohort, including the following: maternal HIV testing history; 12- and 24-month maternal PMTCT/ART program status (i.e., alive in care, transferred out, stopped ART, died, and lost to follow-up [LTFU]); 6- and 24-month maternal VL (timing per national guidelines); infant first HIV-1 DNA PCR result (from 6 weeks of age per national guidelines); 12- and 24-month infant follow-up program status (i.e., alive in care, transferred out, died, and LTFU); and 12- and 24-month infant HIV sero-status (timing of rapid testing per national guidelines) [36,37].

In the second step of data collection, study and MOH staff used detailed locator information available at the clinic to contact all mother–infant pairs sampled for the field survey. Contact with mother–infant pairs took place at routine clinic visits and through community outreach, telephone calls, and/or in-person tracing to introduce them to the study. Mother–infant pairs expressing interest, and who could be reached to complete written informed consent procedures, were asked to complete the field survey. The field survey had 2 parts: (1) a tablet-based questionnaire comprised of a series of electronic case reporting forms to collect information on participant experiences with CFL models and maternal and infant medical histories, including history of VL and EID testing in the routine PMTCT/ART program; and (2) study-initiated blood sample collection for maternal VL and HIV testing on the child from the index pregnancy (i.e., HIV-1 DNA PCR testing for infants ≤12 months and rapid testing for young children >12 months). In cases where next of kin of a deceased mother were reached, a brief verbal autopsy was conducted to confirm the maternal death and to obtain an approximate date of death.

All abstracted routine medical record information was entered directly into a secure study database by trained staff and subjected to data logic constraints at the time of data entry. Field survey data were combined electronically with the routine medical record data, and the resulting merged database was subjected to weekly local and monthly central quality control checks to identify missing and discrepant data. Queries were addressed during weekly study team meetings, and review of source medical records and case reporting forms. Discrepant values for the same variable observed across 2 or more data sources were resolved by committee adjudication involving the principal investigator, senior analyst, and study coordinator. Additional details about data management processes are provided in the study protocol (**S1 Protocol**).

### Laboratory procedures

Field survey participants who had not completed MOH-recommended VL or EID testing at the time of consent had samples taken for routine “catch up” testing. Field survey participants who had received MOH-recommended VL or EID testing, but who did not have a documented result within 6 months of study consent, also had specimens collected for testing. Blood sampling involved finger or heel (for infants ≤12 months) prick for dried blood spot sample collection [36,37]. All study-specific testing was done at the University of North Carolina Project Tidziwe Laboratory (Lilongwe, Malawi). Quantitative HIV-1 VL testing was done on the Abbot RealTime HIV-1 assay (Abbott Laboratories, Chicago, Illinois, USA); qualitative DNA PCR testing for EID was performed on the Xpert platform (Cephied, Sunnyvale, California, USA).

### Statistical analysis

Our study was powered to detect an 8% absolute difference in maternal retention in care between PBFW living with HIV who did, versus those who did not, receive CFL model support per the field survey. With a 2-sided alpha of 0.05, we estimated that we would have to assess outcomes among 894 mother–infant pairs from sites with a CFL model and 298 mother–infant pairs from sites with no CFL model (i.e., a “traditional” standard of care), thus requiring a total of 1,192 PBFW living with HIV, to have 80% power to detect the specified difference in our primary outcome. After protocol development and during programmatic mapping of available CFL models undertaken prior to study data collection, we recognized that a “traditional” standard of care in which no CFL model operated at a site was typically nonexistent. Because of this and the fact that our sample size calculation was based on estimated maternal retention under a no CFL model condition, we conferred a Study Advisory Committee meeting in November 2017 to review our study analysis plan. During that meeting, the Expert Client model was felt to represent an emerging standard of care in the national PMTCT program, since it typically involved MOH-led community outreach and peer support to enhance PMTCT services. As such, this model was considered the referent group for all planned comparative analyses, and we limited analyses for the few mother–infant pairs receiving no CFL model to maternal LTFU and time to maternal ART. Of note, we did not oversample mother–infant pairs for the field survey at sites found to offer a CFL model during programmatic mapping to meet the original sample size.

We used responses from the field survey to identify or impute CFL model exposure. Specifically, for mother–infant pairs who participated in the field survey, we used the CFL model they reported receiving to indicate their exposure. For mother–infant pairs who did not participate in the field survey, we imputed CFL model exposure based on the CFL model most frequently cited by field survey participants as providing services at the site during the period of interest (variation in CFL model exposure as reported by field survey participants was limited at most study sites; **S3 Table**).

Statistical analyses focused on the study cohort as the analysis population. We estimated the impact of CFL model type on primary and secondary outcomes by assessing the associations between CFL model and the cumulative incidence of each outcome. Specifically, we estimated cumulative incidence functions under each model using inverse probability–weighted Aalen–Johansen estimators [42]. We also compared the subdistribution hazard functions (which is not influenced by differences in the duration of cohort member follow-up time) for each outcome of interest, using weighted subdistribution hazard ratios computed using the Fine and Gray method [43]. In analyses of ART initiation, LTFU, and viral suppression, death was treated as a competing event.

In all analyses, we applied sampling weights to account for the uneven site sampling probabilities across districts. Specifically, sampling weights had a value of 1 for all individuals in the first 4 study districts where we included all eligible high-volume sites, and had a value of 2 (i.e., 1 divided by the sampling fraction of 50%) in the last study district where only half of high-volume sites were sampled. We also applied inverse probability weights to all analyses to account for confounding by facility type/level, which standardized the distribution of health facility level between CFL exposure arms [44]. Inverse probability weights were stabilized by the marginal probability of exposure [45] such that they had the form *P* (*A* = *a*)/*P* (*A* = *a*|*L*), where *A* represents CFL exposure arm and *L* represents facility type. The numerator and denominator of the weights were estimated using logistic regression.

We estimated our longitudinal maternal suppression metric by summarizing the percent of days over the 2 years following HIV diagnosis that women spent in HIV care, on ART, and virologically suppressed, and compared this across CFL models. The percent time suppressed was estimated for all eligible women in the cohort over the entire 2-year period by averaging the product of the probability of being retained on treatment (i.e., after starting ART and prior to, or without, becoming LTFU) at any given time point and the probability of being virally suppressed, given one was on ART. The probability of being on treatment at time *t* was calculated as the cumulative incidence of ART initiation minus the cumulative incidence of LTFU, where cumulative incidence functions were estimated using the Aalen–Johansen estimator, which accounted for right censoring and competing events.

The probability of viral suppression, given one was on ART, was estimated at each time point using a 2-stage procedure that made use of both the VL data abstracted from the routine medical record and the VL measurements obtained from the field survey. First, we estimated the probability of having a survey-obtained VL at each time point, given that a patient did not have a routine VL documented in the medical record. Then, we assigned routinely collected VLs a weight of 1 and survey-obtained VLs a weight defined by the inverse probability that a patient had a survey-obtained VL at that time point. In the weighted VL data, we modeled the probability of viral suppression at each time point using a flexible logistic regression model. In this model, we conservatively assumed that the first 30 days on ART were all unsuppressed [46]. After the first 30 days, we allowed the probability of suppression to vary flexibly over time by modeling time since HIV diagnosis using penalized b-splines.

Two subgroup analyses were conducted using data from the field survey. First, among people classified as LTFU using the facility records, we examined the proportion in the field survey who reported receiving care anywhere within the past 6 months to estimate the extent of silent transfer. Second, for infants exposed to HIV in the field survey, we estimated the prevalence and prevalence ratio (PR) of vertical HIV transmission by 18 months under each CFL model.

Random sampling of sites in the last district was done in STATA (Version 14.1, College Station, Texas, USA). All statistical analyses were conducted using SAS version 9.4 (Cary, North Carolina, USA) and R 3.6.0.

## Results

### Participant flow

**Fig 1:**
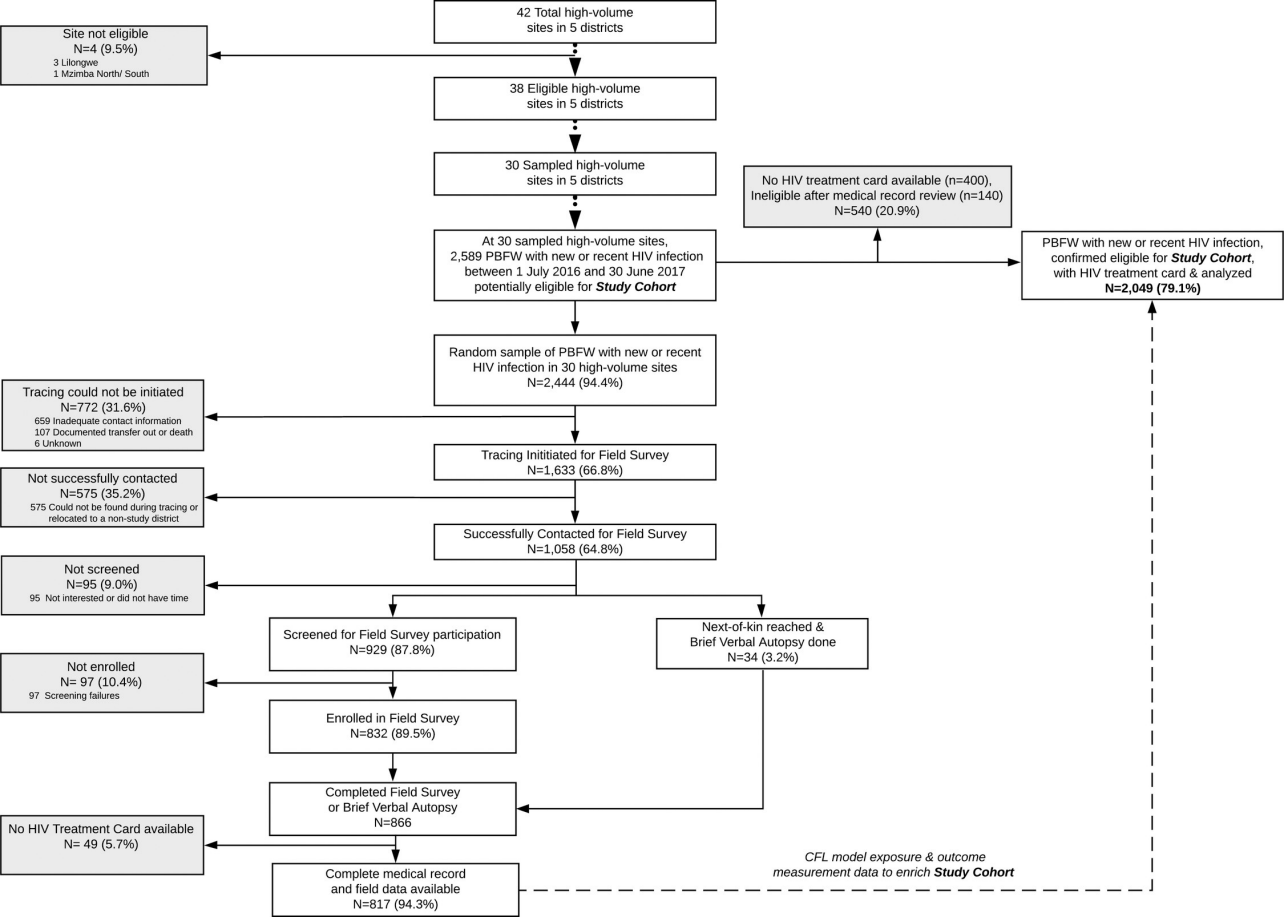
Participant flow through the study. CFL, community-facility linkage; PBFW, pregnant and breastfeeding women.

We reviewed medical records for 2,589 mother–infant pairs potentially eligible for the study cohort (**Fig 1**). Of these, 2,444 were sampled for the field survey (resulting in an overall sampling fraction of 94.4%), and 2,049 had an available HIV treatment card and were analyzed in the study cohort. Of those sampled, 1,633 (66.8%) had interest and/or available locator information to enable tracing procedures, and 1,058 (64.8%) were successfully contacted. Of those contacted, 866 (81.9%) had an outcome ascertained through the field survey (*n =* 832) or brief verbal autopsy (*n* = 34). Overall, 817 (40.0%) of 2,049 mother–infants pairs analyzed had their routine data enriched by field survey or brief verbal autopsy.

### Study cohort characteristics

Most women were 24 years of age or younger (43.7%), received PMTCT services in Lilongwe District (29.8%), and accessed care at a government primary health center (62.2%) (**Table 1)**. According to our classification scheme, 1,763 (86.0%) of mother–infant pairs received support from Expert Clients, CHWs, or Mentor Mothers; 192 (9.4%) received services from ≥2 models; and 94 (4.6%) did not receive any CFL model support. Women who received no CFL model support tended to be younger (i.e., ≤24 years old) than women receiving services from other CFL models. The distribution of models varied by district, as expected, with most women accessing CFL model services at primary health center level. CFL model exposure and other characteristics did not differ substantively between mother–infant pairs overall and those sampled, recruited, and completing the field survey (**S4 Table**).

**Table 1:** Characteristics of PBFW living with HIV in the study cohort by CFL model exposure (*N =* 2,049).

Characteristics		All N = 2,049		No model^a^ N = 94		Community Health Worker^a^ N = 640		Mentor Mother^a^ N = 345		Expert Client^a^ N = 778		≥2 models^a^ N = 192	
		n	%^b^	n	% ^b^	n	% ^b^	n	% ^b^	n	% ^b^	n	% ^b^
Age, years													
	<18	83	5.2	4	7.2	21	4.0	13	4.0	40	6.3	5	2.8
	18–24	698	38.5	30	41.0	209	39.7	111	34.6	269	38.1	79	43.4
	25–29	450	24.8	13	18.1	138	26.2	91	28.4	165	23.8	43	23.6
	30–34	379	21.5	16	27.7	109	20.7	76	23.7	140	20.9	38	20.9
	≥35	178	10.0	5	6.0	50	9.5	30	9.3	76	10.9	17	9.3
	Missing^c^	261		26		113		24		88		10	
District													
	Lilongwe	767	29.8	65	52.8	287	44.8	160	46.4	99	7.7	156	81.3
	Mzimba (North plus South)	400	15.5	0	0.0	0	0.0	185	53.6	179	14.0	36	18.8
	Salima	353	13.7	0	0.0	353	55.2	0	0.0	0	0.0	0	0.0
	Zomba	529	41.0	29	47.2	0	0.0	0	0.0	500	78.2	0	0.0
Facility type/ level													
	Government Primary Health Centre	1,255	62.2	94	100	424	66.3	33	9.6	548	67.9	156	81.3
	Government Rural/ Community Hospital	64	2.5	0	0.0	0	0.0	64	18.6	0	0.0	0	0.0
	Government District Hospital	274	10.6	0	0.0	193	30.2	81	23.5	0	0.0	0	0.0
	Christian Health Association of Malawi (CHAM) Facility	456	24.7	0	0.0	23	3.6	167	48.4	230	32.1	36	18.8
WHO Stage at PMTCT/ ART enrolment													
	1	1,897	99.8	91	100	615	99.5	260	99.6	748	100	183	100
	2	4	0.2	0	0.0	3	0.5	1	0.4	0	0.0	0	0.0
	Missing^c^	148		3		22		84		30		9	

^a^Based on participant self-report, where available, and, where not available, based on single imputation derived from distribution of field survey responses.

^b^Percentage is weighted to account for variation in site sampling across districts.

^c^Missing data were not included in the denominator for calculation of percentages for characteristics of interest.

WHO, World Health Organization; PMTCT, prevention of mother-to-child transmission of HIV; ART, antiretroviral therapy

### Maternal outcomes

**Fig 2:**
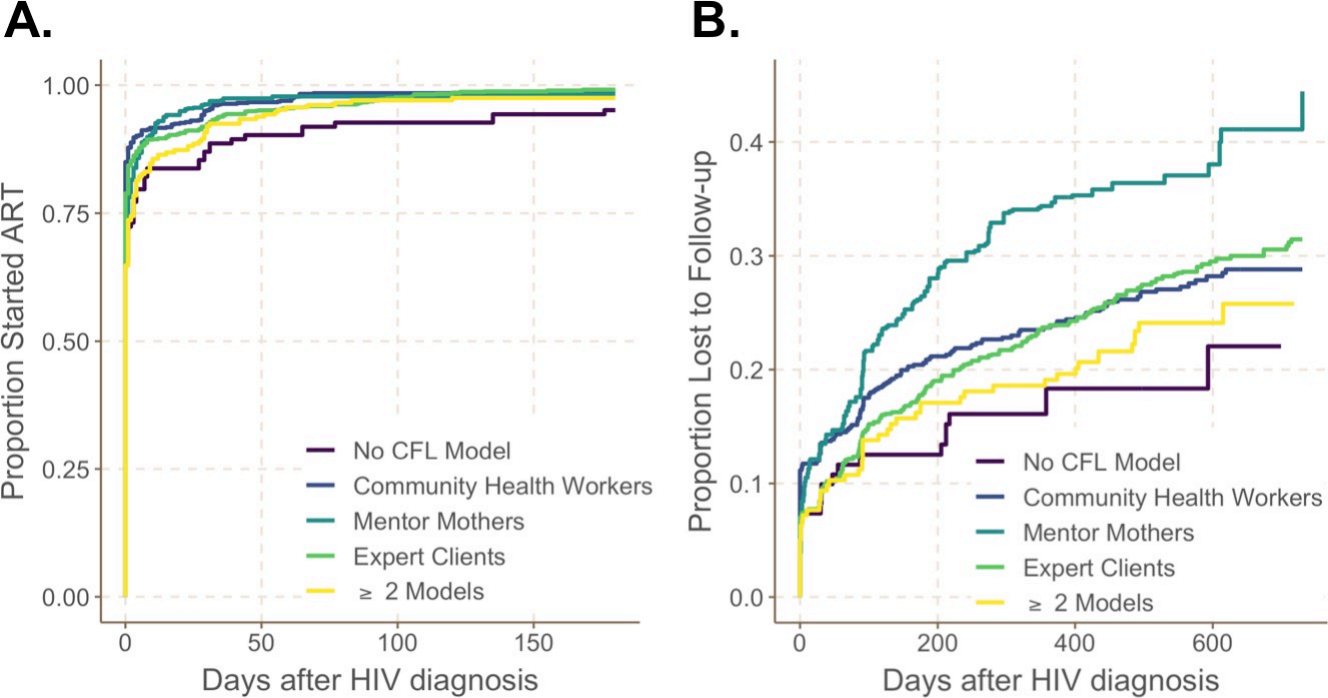
Maternal ART uptake and LTFU by CFL model (*N =* 2,049). (A) Proportion of PBFW living with HIV who started ART. (B) Proportion of PBFW living with HIV lost to follow-up in the national PMTCT/ART program. ART, antiretroviral therapy; CFL, community-facility linkage; LTFU, loss to follow-up; PBFW, pregnant and breastfeeding women; PMTCT, prevention of mother-to-child transmission of HIV.

Of 2,049 total PBFW living with HIV analyzed, 98.5% (95% confidence interval [CI]: 98.0%, 99.0%) started ART by 6 months after their HIV diagnosis (**Table 2).** Time to ART initiation was uniformly rapid, with nearly 90% starting ART within 7 days of HIV diagnosis, consistent with treatment timing under treat all policy (**Fig 2A**). Women receiving the CHW model were 1.13 (95% CI: 1.01, 1.26; *p* = 0.04) times as likely to start ART during the 6 months after HIV diagnosis as those exposed to the Expert Client model. Conversely, compared to women supported by Expert Clients, women receiving the Mentor Mother model (adjusted hazard ratio [aHR]: 0.88, 95% CI: 0.78, 0.99; *p* = 0.03), ≥2 CFL models (aHR: 0.79, 95% CI: 0.68, 0.93; *p* = 0.004), and no CFL model (aHR: 0.76, 95% CI: 0.60, 0.96; *p* = 0.02) were all significantly less likely to initiate ART during the first 6 months following HIV diagnosis (**Table 2**).

**Table 2:** Comparisons of maternal ART initiation and PMTCT/ART program LTFU by CFL model among PBFW living with HIV (*N =* 2,049).

CFL Model	N	No. Events	Unadjusted HR^a^	95% CI	*p*-value	aHR^a^^,^^b^	95% CI	*p*-value
**Maternal ART initiation** ^c^	**2,049**	**2,015**						
Expert Clients	778	770	Ref	—	—	Ref	—	—
CHWs	640	632	1.17	1.06, 1.30	0.002	1.13	1.01, 1.26	0.04
Mentor Mothers	345	337	0.85	0.76, 0.95	0.006	0.88	0.78, 0.99	0.03
≥2 Models	192	188	0.81	0.70, 0.94	0.006	0.79	0.68, 0.93	0.004
No CFL model	94	88	0.76	0.60, 0.95	0.02	0.76	0.60, 0.96	0.02
**Maternal LTFU** ^d^	**2,049**	**574**						
Expert Clients	778	225	Ref	—	—	Ref	—	—
CHWs	640	168	1.00	0.81, 1.22	0.98	0.98	0.79, 1.23	0.89
Mentor Mothers	345	116	1.33	1.06, 1.66	0.01	1.45	1.14, 1.84	0.003
≥2 Models	192	45	0.85	0.61, 1.17	0.32	0.82	0.59, 1.14	0.23
No CFL model	94	20	0.69	0.43, 1.13	0.14	0.68	0.42, 1.11	0.12

^a^All HRs are weighted to account for variation in site sampling across districts.

^b^Adjusted by inverse probability weighting to account for confounding by facility type/level.

^c^Event defined as documented ART initiation in the routine medical record.

^d^Event defined as a documented LTFU in the routine medical record.

aHR, adjusted hazard ratio; ART, antiretroviral therapy; CFL, community-facility linkage; CHW, Community Health Worker; CI, confidence interval; HR, hazard ratio; LTFU, loss to follow-up; PBFW, pregnant and breastfeeding women; PMTCT, prevention of mother-to-child transmission of HIV; SOC, standard of care.

PBFW in the study cohort were followed for a median of 472 (interquartile range [IQR]: 150 to 658) days in care. Overall, 29.8% of these women experienced LTFU by 2 years after HIV diagnosis. Time to LTFU varied by CFL model (**Fig 2B**). Compared to women receiving the Expert Client model, women receiving the Mentor Mother model were significantly more likely to be lost (aHR: 1.45, 95% CI: 1.14, 1.84; *p* = 0.003) in the adjusted analysis (**Table 2**). Conversely, women receiving the CHW model (aHR: 0.98, 95% CI: 0.79, 1.23; *p* = 0.89), ≥2 models (aHR: 0.82, 95% CI: 0.59, 1.14; *p* = 0.23), and no CFL model (aHR: 0.68, 95% CI: 0.42, 1.11; *p* = 0.12) were nonsignificantly less likely to be lost in the adjusted analysis (**Table 2**). Of 155 PBFW living with HIV who were classified as LTFU using facility data and were sampled, recruited, and completed a field survey, only 10 (6.5%) reported receiving care in the past 6 months.

**Fig 3:**
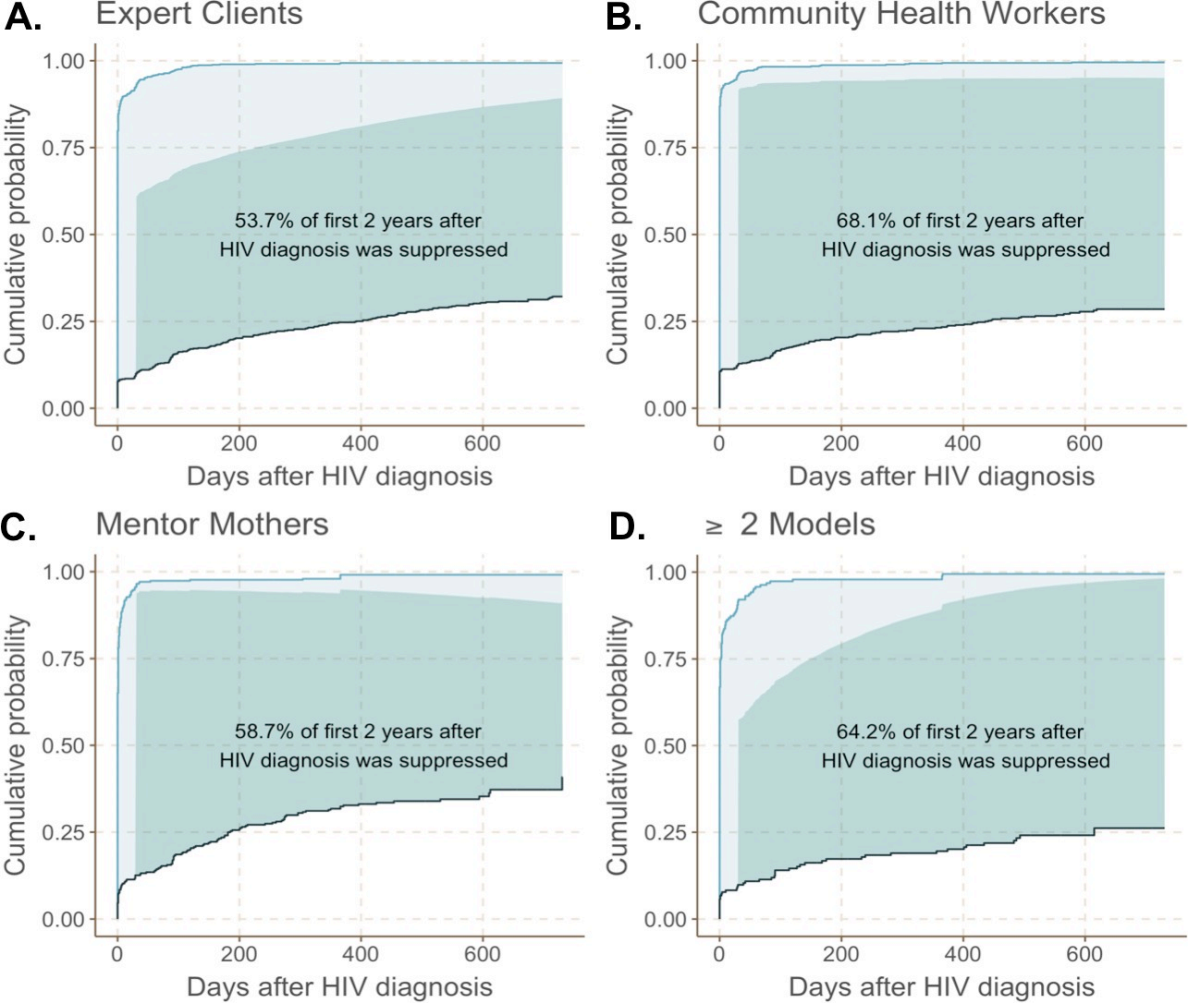
Comparative evaluation of longitudinal maternal virological suppression under 4 different CFL models (*N =* 2,049). Each panel presents, by CFL model type, the cumulative incidence of ART initiation (blue curves), LTFU (black curves), number of days on ART (total shaded area), and number of days with virological suppression (dark shaded area), weighted to account for sampling variation across districts and confounding by health facility level. The first 30 days after HIV diagnosis was not included in estimates of number of days with virological suppression. (A) Expert Client model. (B) CHW model. (C) Mentor Mother model. (D) ≥2 models. ART, antiretroviral therapy; CFL, community-facility linkage; CHW, Community Health Worker; LTFU, loss to follow-up.

For women on treatment, the average probability of being virologically suppressed averaged over 2 years of follow-up from ART initiation was 77.5% (95% CI: 72.6%, 82.4%) for the Expert Client model, 92.0% (95% CI: 87.7%, 96.3%) for the CHW model, 90.0% (95% CI: 84.3%, 95.7%) for the Mentor Mother model, and 93.3% (95% CI: 85.7%, 100.0%) for women receiving ≥2 models. We used these probabilities of virological suppression for women on ART to develop our longitudinal maternal suppression metric represented by the cumulative incidence curves in **Fig 3**. The dark shaded area in each panel represents the proportion of days women spent over 2 years after HIV diagnosis in an optimal state of being retained in care, on ART, and virally suppressed. By CFL model, then, the percentage of days women spent both retained on ART and with viral suppression was 53.7% for the Expert Client model (**Fig 3A**), 68.1% for the CHW model (**Fig 3B**), 58.7% for the Mentor Mother model (**Fig 3C**), and 64.2% for ≥2 models (**Fig 3D**). Compared to women receiving the Expert Client model, women supported by CHWs spent a significantly higher proportion of days—14.3% (95% CI: 2.6%, 26.1%; *p* = 0.02) more—in care, on ART, and virally suppressed. Similarly, women supported by the Mentor Mother model and ≥2 models spent 4.9% (95% CI: −6.7%, 16.6%; *p* = 0.42) and 10.4% (95% CI: −12.0%, 32.9%; *p* = 0.37) more days, respectively, in care, on ART, and virally suppressed than women receiving the Expert Client model, although these estimated differences were not statistically significant.

### Infant outcomes

**Fig 4:**
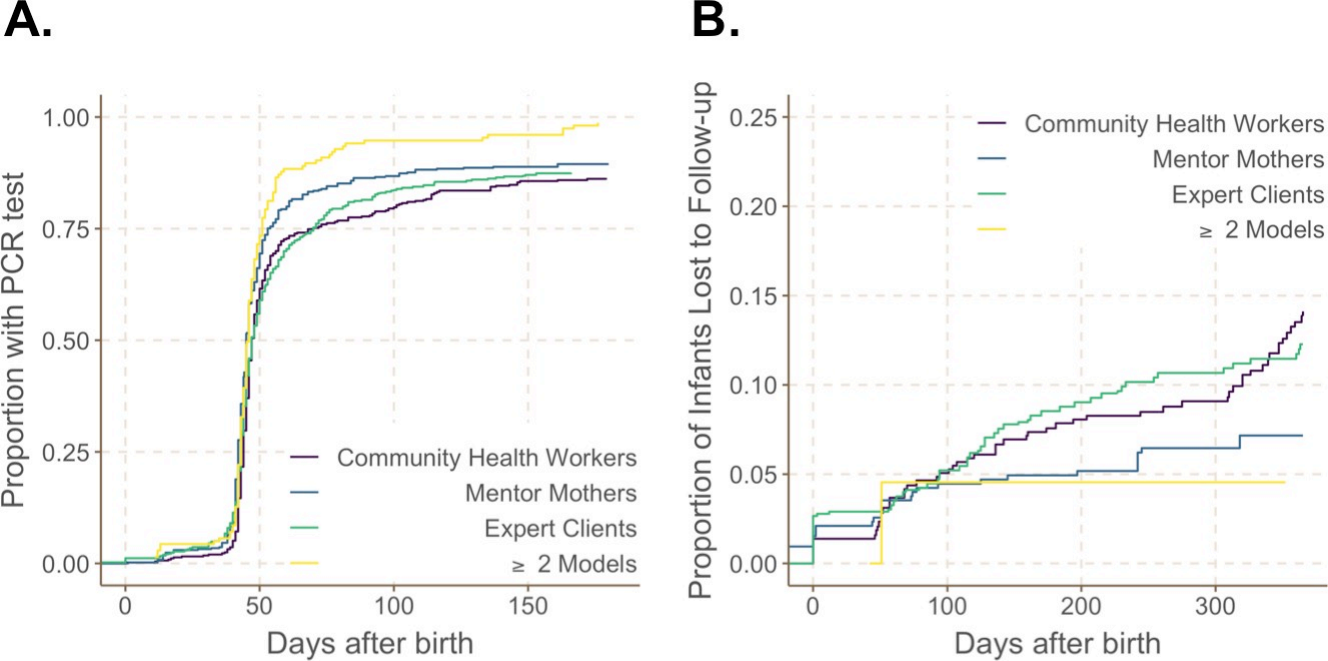
EID testing uptake and LTFU by CFL model among infant exposed to HIV in the national PMTCT/ART program (*N =* 1,326). (A) Proportion of infants exposed to HIV with a documented HIV-1 DNA PCR test result. (B) Proportion of infants exposed to HIV who experienced LTFU. ART, antiretroviral therapy; CFL, community-facility linkage; EID, early infant diagnosis; LTFU, loss to follow-up; PMTCT, prevention of mother-to-child transmission of HIV.

Among mother–infant pairs analyzed, 1,549 (75.6%) had an available infant medical record. Of these, 1,326 (85.6%) infants exposed to HIV had complete EID testing documentation and 1,260 (81.3%) a complete routine follow-up care record. The proportion who received EID testing by 6 months (i.e., 180 days) of age was above 80% for all models (**Fig 4A**). Infants supported by ≥2 models (aHR: 1.44, 95% CI: 1.20, 1.74; *p* < 0.001) and the Mentor Mother model (aHR: 1.24, 95% CI: 1.01, 1.52; *p* = 0.04) were significantly more likely to undergo EID (i.e., DNA PCR) testing by 6 months of age than infants receiving the Expert Client model (**Table 3**). Infants exposed to the CHW model (aHR: 0.96, 95% CI: 0.82, 1.12; *p* = 0.58) were as about as likely to undergo EID testing by 6 months as infants receiving the Expert Client model.

**Table 3:** Comparison of infant health outcomes by CFL model over the first year of life (*N* = 1,260).

CFL Model	N	No. Events	Unadjusted HR^a^	95% CI	*p*-value	aHR^a^^,^^b^	95% CI	*p*-value
**Infant EID testing** ^c^								
Expert Clients	512	448	Ref			Ref	–	–
CHWs	386	334	0.96	0.83, 1.10	0.54	0.96	0.82, 1.12	0.58
Mentor Mothers	234	211	1.31	1.10, 1.56	0.003	1.24	1.01, 1.52	0.04
≥2 Models	128	126	1.34	1.12, 1.59	0.001	1.44	1.20, 1.74	<0.001
**Infant LTFU** ^d^								
Expert Clients	512	58	Ref			Ref	–	–
CHWs	386	55	1.16	0.80, 1.68	0.44	1.08	0.74, 1.59	0.68
Mentor Mothers	234	16	0.71	0.40, 1.25	0.23	0.69	0.36, 1.32	0.26
≥2 Models^e^	23	1	0.64	0.09, 4.42	0.65	0.64	0.09, 4.33	0.64
**Infant composite poor outcome** ^f^ ^,^ ^g^								
Expert Clients	512	71	Ref			Ref	–	–
CHWs	386	68	1.17	0.84, 1.64	0.61	1.15	0.80, 1.67	0.44
Mentor Mothers	234	24	0.89	0.55, 1.42	0.29	0.84	0.50, 1.42	0.51
≥2 Models^e^	23	2	1.11	0.29, 4.26	0.67	1.08	0.29, 4.11	0.91

^a^All HRs are weighted to account for variation in site sampling across districts.

^b^Adjusted by inverse probability weighting to account for confounding by facility type/level.

^c^Events defined as documented EID test result reported in the routine medical record within 6 months of birth.

^d^Event for “Infant LTFU” defined as a documented LTFU by 1 year of age in the routine medical record.

^e^Excludes 1 health facility with incomplete infant follow-up data.

^f^Composite poor outcome encompassing: documentation of a positive HIV test result, LTFU, or death.

^g^Event for “Infant composite poor outcome” defined as any documented evidence in the routine medical record of a positive HIV test result, LTFU, or death by 1 year of age.

aHR, adjusted hazard ratio; CFL, community-facility linkage; CHW, Community Health Worker; CI, confidence interval; EID, early infant diagnosis; HR, hazard ratio; LTFU, loss to follow-up.

On average, infants exposed to HIV were followed for a median of 524 days (IQR: 390 to 686) in PMTCT care. Across models, less than 15% of infants exposed to HIV were lost to follow-up at 12 months (**Fig 4B**). Compared to infants receiving the Expert Client model (12.2%), 12-month LTFU risk was numerically higher among infants receiving the CHW model (14.1%) and numerically lower for infants receiving the Mentor Mother model (7.2%) or ≥2 models (4.5%). Infants exposed to HIV receiving the Mentor Mother model were 0.69 times as likely (95% CI: 0.36, 1.32; *p* = 0.26) to be LTFU at 12 months as infants who received Expert Client services, but not significantly so.

Over 12 months of follow-up, infants exposed to HIV had a risk of composite poor outcome that varied by CFL model, but without any statistically significant differences: 14.9% for infants who received the Expert Client model, 18.2% for infants exposed to the CHW model, 10.5% for infants supported by the Mentor Mother model, and 9.1% for infants who received ≥2 models. Infants receiving the CHW, Mentor Mother, and ≥2 models were 1.15 (95% CI: 0.80, 1.67; *p* = 0.44), 0.84 (95% CI: 0.50, 1.42; *p* = 0.51), and 1.08 (95% CI: 0.29, 4.11; *p* = 0.91) times as likely, respectively, to experience a poor outcome at 1 year compared to those receiving the Expert Client model; however, these results were not statistically significant (**Table 3**).

Relatively few infants had a final HIV status determination documented in the routine record by 18 months of age. A total of 44 (2.8%) infants were determined to be HIV infected, of whom 36 (81.8%) were known to be alive, in care, and on ART; 4 of these infants were newly HIV diagnosed during the field survey (**S5 Table**). While we could not reliably estimate the final vertical transmission risk for all mother–infant pairs because of missing infant medical record data, we could make limited inferences based on EID testing data from the field survey. For this subgroup, we estimated the prevalence and PR of vertical transmission at 18 months by CFL model as follows: Expert Client model (2.6%; PR: n/a [referent]); CHW model (3.1%; PR: 1.22, 95% CI: 0.45, 3.11); Mentor Mother model (0.98%; PR: 0.43, 95% CI: 0.22, 3.17); and ≥2 models (6.3%; PR: 2.43, 95% CI: 0.00, 22.39).

## Discussion

For this descriptive study, we conducted the first comparative evaluation, to our knowledge, of CFL models from a real-world PMTCT program in the “treat all” era. Across the study sites, we noted widespread coverage of Mentor Mother, CHW, and Expert Client models in Malawi’s national PMTCT/ART program, collectively reaching an estimated 95% of mother–infant pairs. Whereas previous research has focused largely on characterizing individual CFL models, we aimed to compare maternal and infant outcomes across multiple models. In so doing, we observed important differences in maternal and infant health outcomes by CFL model at different steps along the PMTCT continuum.

Due to a rapidly evolving and improving PMTCT landscape in Malawi, most study sites had implemented at least 1 CFL model. As such, our ability to make comparisons to our originally planned referent group (i.e., no CFL model support) was limited to maternal LTFU and maternal ART initiation. Our decision to use the Expert Client model as the referent group was scientifically sound, increased our precision to estimate the impacts of CFL models on maternal and infant outcomes, and was based on evidence from a preimplementation mapping exercise that established Expert Clients as the most prevalent CFL model. In so far as the Expert Client model is officially adopted by the MOH, it could represent an emerging standard of care for PMTCT programming in Malawi, particularly in settings without NGO support since the Expert Client model may be less resource intensive to implement than other models [34].

Early within the PMTCT continuum, our time to event analyses suggest that the CHW model was associated with slightly higher, and the Mentor Mother model slightly lower, rates of maternal ART initiation compared to the Expert Client model. These differences notwithstanding, most women initiated ART soon after their HIV diagnosis irrespective of CFL model, consistent with Malawi’s Option B+ policy and more recent national guidelines advancing a rapid “treat all” approach during the study period [36,47]. While rapid time to ART has been shown to improve retention in care and viral suppression in some settings [48,49], in the context of PMTCT, faster time to ART, and in particular starting ART on the day of HIV diagnosis, may not necessarily translate into superior program outcomes [4,50]. Later in the PMTCT continuum, more pronounced differences in outcomes between CFL models were apparent. For example, compared to the Expert Client model, maternal LTFU was higher for women supported by the Mentor Mother model and lower when women received ≥2 CFL models. The greater focus of Mentor Mothers on providing facility-based services during the study period may have contributed to the higher LTFU seen, as more intensive community tracing and “back to care” services known to improve retention were not as central to the model at that time [51]. Indeed, prior studies have suggested lower maternal retention when peer support is provided in facilities rather than in the community [25]. The higher LTFU observed among women receiving Mentor Mother support could also be explained, in part, by greater care disengagement prior to ART start given the lower ART initiation seen in this group. Finally, LTFU could have been influenced by how health facilities organized their PMTCT/ART services at study sites. Prior work from Malawi indicates that LTFU may be higher at facilities where women initiate ART in the antenatal clinic before being referred to the ART clinic for ART continuation and follow-up [38]. Associations between CFL model and LTFU were strongest during the first 6 months after HIV diagnosis, consistent with prior work showing this time to be a particularly risky period for PMTCT client disengagement from HIV care [4,35].

Few studies have described the impact of CFL models on maternal viral suppression. We noted that 90% or more of women on ART receiving the CHW, Mentor Mother, or ≥2 models experienced viral suppression, which was higher than that seen among women receiving the Expert Client model. The proportion of ART-treated women with viral suppression we observed is comparable to figures reported previously from Malawi for PMTCT clients retained in care and women reporting a HIV diagnosis and being on ART [40,52]. Further research is needed to understand how CFL model services influence ART adherence in support of viral suppression [53], and how CFL models may affect identification of possible virological failure and treatment regimen change (as necessary) [52].

Sustaining ART and viral suppression over time are the ultimate goals of the second and third 95s for PBFW living with HIV [54]. As such, we applied a novel longitudinal suppression metric to estimate the cumulative time women spent in an optimal state of being retained in care, on ART, and virally suppressed in each CFL model. Unlike previous studies, this metric allowed us to estimate average viral suppression over a 2-year follow-up period and to make use of field survey data that accounted for women who had episodes of care disengagement and/or who missed routine VL testing in the national PMTCT/ART program. Looking at the PMTCT continuum in this way, women who received support from the CHW model spent significantly more days over 2 years—14.3% more—in care, on ART, and with a VL <1,000 copies/ml than women receiving other models. While descriptive data from Malawi have revealed the contributions CHW models make to promoting maternal engagement with the PMTCT continuum, particularly around PMTCT program enrollment and ART uptake [23,27,50], few reports have examined the impacts of CHW models on long-term maternal outcomes [20]. Evidence from the early Option B+ era suggests suboptimal maternal retention and postdelivery ART maintenance despite the availability of CHW support [50]. Our study’s longitudinal maternal suppression metric points to the beneficial impact that CHWs have on helping women spend more time in an optimal state of HIV care within the PMTCT continuum. We postulate that this impact was due to comparatively greater nongovernmental resourcing to sites implementing the CHW model, and an explicit mandate for CHWs to move between facilities and communities to support PMTCT service delivery and actively trace clients who had fallen out of care [23,27]. While greater governmental resourcing could have also played a role, particularly at large district hospitals where one-third of CHW model beneficiaries accessed PMTCT, it is unlikely to have affected the magnitude of our estimated associations since we controlled for facility type/level in our analysis through inverse probability weighting. The superior outcomes we observed among women who received the CHW model have implications for how health systems organize CFL model services. Our findings suggest that some of the general health system strengthening activities specific to CHWs, described in detail elsewhere [27], may generate long-term benefits for PBFW living with HIV, arguing for a more diagonal approach to PMTCT programming and CFL model integration [55].

Apparent impacts of CFL models on infant outcomes were mixed. For EID testing, overall uptake within the first 2 months of life approached 75% for all models, which was higher than previous population-based estimates from Malawi [40]. Disaggregated by CFL model, infants exposed to HIV who received the Mentor Mother model or ≥2 models had higher testing rates by 6 months of age than those supported by Expert Clients. Similarly, infants receiving the Mentor Mother model or ≥2 models had a numerically lower risk of LTFU by 12 months of age. For our composite infant poor outcome, infants exposed to HIV who received the Mentor Mother model seemed to fair best, being 0.84 times as likely to experience death, LTFU, or a positive HIV test result as infants who received Expert Client services, although this finding was not statistically significant. Our findings echo results from other studies on the Mentor Mother model in the region. In Zimbabwe, for example, PMTCT clients receiving Mentor Mother support were twice as likely to return with their infants for EID testing compared to unsupported clients [56], and, in Uganda, vertical transmission at 18 months was lower for infants at facilities with (6.8%), versus those without (8.7%), Mentor Mother services [28]. In the Malawi PURE trial, facility-based peer support akin to that provided by Mentor Mothers was associated with higher infant HIV testing uptake (80%) at 1 year compared to community-based support provided by Expert Clients (68%) (although PURE was not powered to detect a difference between these 2 peer support approaches) [25].

We estimated that the same CFL model was differentially associated with HIV outcomes for mothers and infants at different steps in the PMTCT continuum. Such differential impacts suggest that the individual features and services that comprise CFL models may be delivered with varying focus, fidelity, and effectiveness as mother–infant pairs progress through the continuum and may be interacting with the health system in different ways depending on the setting to support delivery of HIV testing, treatment, and care [34]. While similar complexity has been seen in other studies (e.g., the differential impacts of facility-based peer support on maternal LTFU and infant testing noted above in the PURE study), our findings nonetheless emphasize the need to further characterize the key individual features that constitute CFL models. Indeed, the different outcomes experienced by mothers and infants receiving the same model may reflect variability in implementation of key CFL model features, including the frequency and timing of client contacts, attention to back-to-care tracing, and other pragmatic supports to mother–infant pairs [26]. Understanding the association between these CFL model features and the time women spend in an optimal state of viral suppression will be the focus of a forthcoming analysis and can help advance an essential minimum package of CFL services for PMTCT programs. For example, frequent client home visits or low client-to-staff ratios may be necessary to further a client’s trust in a CFL provider, afford CFL providers more time to interact with their clients, or enable individualized counseling and client-centered support to promote longitudinal maternal care retention and viral suppression. To support government ownership and long-term maintenance of CFL models, costing the essential CFL model features driving maternal and infant outcomes is also critical and should be an area of future investigation. Similarly, there is a need for further study of the materials inputs, interactions with the public health system, and fidelity to protocols required to properly implement CFL models in settings that differ by demography, health system tier, and investments in human resources for health [34].

We acknowledge several limitations with our study. First, at some sites, mother and infant records were either incomplete or unlinked, resulting in missing data that were encountered more frequently in infant than maternal records. Although we accounted for missing data on CFL model exposure and maternal VL through imputation based on field survey data, we could not adjust for imbalances in the distribution of unmeasured sociodemographic characteristics and other covariates across CFL types, which may have introduced bias or limited the accuracy of our estimates due to residual confounding. Moreover, as our study was descriptive in nature, any observed association between CFL model type and study outcome should not be interpreted as implying causation. Second, missing contact details in the routine medical record made it challenging to contact—either by phone or via home visits—some mother–infant pairs for the field survey. As a result, even though a high proportion of cohort members were sampled for the field survey, only 40% completed a survey or verbal autopsy, and, thus, possible selection bias in field survey administration may have resulted in survey respondents not being fully representative of the larger cohort. As such, although we used exhaustive tracing procedures and study and programmatic resources to maximize field survey participation, it is likely that our field survey respondents included mother–infant pairs more likely to have had contact with the health system, thereby limiting the utility of the field survey for making generalizable inferences about the larger cohort. Missing data in the routine records made accounting for this limitation intractable using available quantitative approaches. This limitation notwithstanding, we are reassured by the fact that our maternal and infant retention figures were comparable to those reported by the national PMTCT program overall [6]. Third, the use of single imputation to estimate our exposure at sites where survey respondents reported an even distribution of CFL model services may have led to exposure misclassification. However, the CFL models selected for imputation at the 5 sites where this was an issue generally agreed with the predominant CFL model reported by the site during programmatic mapping and our CFL model survey. Fourth, changes to CFL models over time could have introduced secular trend bias; however, findings from an embedded concurrent mixed methods evaluation argue against there having been major changes in the ways CFL models provided support to mother–infant pairs during the study period [34]. Fifth, our study was limited to high-volume health facilities in 5 purposively selected districts in Malawi, and, as such, our results may not be generalizable to smaller facilities, other districts in Malawi, or settings outside Malawi. Sixth, because of correlations between district and CFL model type, it is possible that any differential associations between CFL model and study outcomes were due, at least in part, to differences in district-level health system resourcing and infrastructure, which we could not account for fully due to collinearity between district and CFL model in our analyses. Lastly, final HIV status ascertainment at 12 to 24 months of age (or 6 weeks following cessation of breastfeeding) was frequently missing in the routine infant medical record. Due to these missing data and the relatively few observed vertical transmission events, we could not reliably estimate the final mother-to-child transmission risk for the entire study population. We sought to mitigate these limitations by using a pragmatic study design, which applied a field survey with biomarker assessment and probability sampling to help overcome missing exposure and outcome data in the medical record for the study cohort.

## Conclusions

We observed widespread reach of CFL models in Malawi and important differences in maternal and infant HIV outcomes by CFL model at different steps along the PMTCT continuum. We noted favorable maternal outcomes in the CHW model and greater infant EID testing uptake in the Mentor Mother model. Examining maternal outcomes longitudinally, we found that women supported by the CHW model spent significantly more time in an optimal state of PMTCT/ART care—retained on ART and virologically suppressed—than women receiving any other model. Although caution should be applied when extending our findings to other settings, our results point to important differences in maternal and infant HIV outcomes by CFL model and imply future opportunities to identify key CFL model features driving these outcome differences to form the basis of an “essential” CFL model package for the PMTCT/ART program in Malawi and possibly other similar programs in Africa.

## Supporting information

S1 Protocol Study protocol. (PDF)

S1 STROBE Checklist STROBE checklist. (DOCX)

S1 Appendix Community-facility linkage model survey. (PDF)

S1 Table CFL model activities and characteristics (*N* = 15 respondents). (DOCX)

S2 Table Field survey sampling by study site. (DOCX)

S3 Table Distribution of community-facility linkage model exposure as reported by field survey participants, by study site. (DOCX)

S4 Table Characteristics of pregnant and breastfeeding women living with HIV in the study cohort (*N* = 2,049) and a subset of field survey participants (*N* = 817). (DOCX)

S5 Table Last known program status among infants exposed to HIV at up to 18 months of follow-up in Malawi’s PMTCT/ART program (*N* = 1,549). (DOCX)

## Abbreviations

aHR: adjusted hazard ratio
ART: antiretroviral therapy
CFL: community-facility linkage
CHW: Community Health Worker
CI: confidence interval
EID: early infant diagnosis
IQR: interquartile range
LTFU: loss to follow-up
MOH: Ministry of Health
NGO: nongovernmental organization
PBFW: pregnant and breastfeeding women
PMTCT: prevention of mother-to-child transmission of HIV
PR: prevalence ratio
VL: viral load
