# Supplementary material for: Community-facility linkage models and maternal and infant health outcomes in Malawi’s PMTCT/ART program: A cohort study
Source: PLoS Med. 2021 Sep 17;18(9):e1003780. doi: 10.1371/journal.pmed.1003780 (PMC8516224; doi:10.1371/journal.pmed.1003780)
Supplement: S1 Protocol — (PDF) [file pmed.1003780.s001.pdf]

Full Protocol Title: **Promoting Mother-Infant Retention along the HIV Care Continuum: A Comparative Effectiveness Evaluation of Three Models for Community-Facility Linkage**

Short Title: **Malawi Mother-Infant Retention**

Version 1.2, 3 October, 2017

Principal Investigator: Michael Herce, MD, MPH, MSc

Co-Principal Investigator: Innocent Mofolo, BSc, MSc

Co-Investigators:
Nicole Carbone, MPH
Maganizo Chagomerana, MSc, PhD
Chileshe Chilangwa, MSc, BSc
Jacqueline Chinkonde, PhD
Jess Edwards, PhD, MPH
Michael Eliya, MSc
Irving Hoffman, PA, MPH
Mina Hosseinipour, MD, MPH
Joseph Njala, DCM, MPH
Emmanuel Singogo, MSc, PhD
Stephanie Topp, PhD, MPhil, MPH
Emily Wroe, MD, MPH
Chifundo Zimba, PhD

Location of Study (Districts): Mzimba North/South, Lilongwe, Salima, Zomba, and Neno

Duration: 26 months

Funding Source: This project is supported by USAID and PEPFAR

Table of Contents

Personnel and Institutions 3
1.0 Abstract 5
2.0 Background and Justification 6
3.0 Literature Review 7
4.0 Central Hypothesis 10
5.0 Goal and Objectives 11
5.1 Overall Research Goal 11
5.2 Objectives 11
6.0 Methodology 11
6.1 Study Design Overview 11
6.2 Study Setting 12
6.3 Study Population 12
6.4 Sample Size 13
6.5 Study Procedures by Objective 18
6.6 Data Collection & Management 39
7.0 Data analysis 41
7.1 Study Outcomes 41
7.2 Analysis Plan by Objective 42
8.0 Results Dissemination & Expected Results 48
8.1 Results Dissemination Strategy 48
8.2 Community Engagement 49
8.3 Expected Results 49
9.0 Ethical considerations 50
9.1 Overview 50
9.2 Physical Risks 50
9.3 Psychosocial Risks 50
9.4 Methods to Minimise Risk 50
9.4 Anticipated Benefits to Participants 52
9.5 Participant Compensation 52
9.6 Safety Monitoring and Unanticipated Problems 52
9.7 Study Discontinuation 54
9.8 Regulatory Review 54
10.0 Capacity Strengthening 54
11.0 Work Plan 55
12.0 Budget 56
12.1 Budget Justification 56
13.0 References 57

Personnel and Institutions

Principal Investigator

Michael Herce, MD, MPH, MSc
Assistant Professor, Dept. of Medicine,
University of North Carolina, Chapel Hill
130 Mason Farm Rd.,
Bioinformatics Building, CB# 7030
Chapel Hill, NC 27599-7030, USA
Phone: +1-919-966-2537
Fax: +1-919-966-6714
Mobile (Malawi): +265-888-960-558
Mobile (Zambia): +260-962-220-145
Email: michael_herce@med.unc.edu

Co-Principal Investigator

Innocent Mofolo, MSc
Executive Director, Lilongwe Medical Relief Fund Trust
Administrative Director, UNC Project
University of North Carolina Project
Tidziwe Centre
Kamuzu Central Hospital
Mobile (Malawi): +265-888-202-152
Email: imofolo@unclilongwe.org

Co-Investigators

Nicole Carbone, MPH
SOAR Study Coordinator
UNC Project-Malawi
Tidziwe Centre,
Private Bag A-104
Lilongwe, Malawi
Phone (Malawi): +265-991-927-419
Email: nicolebcarbone@gmail.com

Maganizo Chagomerana, MSc, PhD
Head of Analysis and Manuscript Writing Unit,
UNC Project-Malawi
Tidziwe Centre,
Private Bag A-104
Lilongwe, Malawi
Email: mchagomerana@unclilongwe.org

Chileshe Chilangwa, BSc, MSc
Country Director
mothers2mothers Malawi
Private Bag B315, Lilongwe, Malawi
Tel: +265 1 774124
Email:
chileshe.chilangwa@m2m.org**Jacqueline Chinkonde, PhD**
UNICEF Malawi
P.O. Box 30375
Airtel Complex
Area 40/31
Lilongwe 3, Malawi
Phone (Malawi): +265-177-0770
Fax: +265-177-3163
Email: jnkhoma@unicef.org

Jessie Edwards, PhD, MSPH
Assistant Professor of Epidemiology
University of North Carolina, Chapel Hill
2102E McGavran-Greenberg Hall, CB#7435
Phone (US): +1-919-966-7438
Email: jessedwards@unc.edu

Michael Eliya, MSc
Department of HIV and AIDS,
Ministry of Health,
P.O. Box 30377
Lilongwe 3, Malawi
Mobile (Malawi): +265-888-867-569
Email: michael.eliya86@gmail.com

Irving Hoffman, PA, MPH
Professor, Dept. of Medicine,
University of North Carolina, Chapel Hill
International Director, UNC Project-Malawi
Tidziwe Centre,
Private Bag A-104
Lilongwe, Malawi
Mobile (Malawi): +265-888-846-526
Email: irving_hoffman@med.unc.edu

Mina Hosseinipour, MD, MPH
Professor, Dept. of Medicine,
University of North Carolina, Chapel Hill

Chief Scientific Officer, UNC Project-Malawi
Tidziwe Centre,
Private Bag A-104
Lilongwe, Malawi
Mobile (Malawi): +265-888-202-153
Email: mina_hosseinipour@med.unc.edu

Joseph Njala, DCM, BSc, MPH
SOAR Study Manager
UNC Project-Malawi
Tidziwe Centre,
Private Bag A-104
Lilongwe, Malawi
Phone (Malawi): +265-888-450-254
Email: jnjala@unclilongwe.org

Emmanuel Singogo, MSc, PhD
Research Manager
Dignitas International
Zomba, Malawi
Phone (Malawi): +265-888-607-969
Email: e.singogo@dignitasinternational.org

Stephanie Topp, PhD, MPhil, MPH
Senior Lecturer in Global Health & Development
James Cook University
James Cook Drive
Townsville, 4812, QLD, Australia
Phone (Australia): +61-747-813-476
Email: globalstopp@gmail.com

Emily Wroe, MD, MPH
Chief Medical Officer
Abwenzi Pa Za Umoyo/ Partners In Health
Neno District Hospital
P.O. Box 56
Neno, Malawi
Mobile (Malawi): +265-888-282-535
Email: ewroe@pih.org

Chifundo Zimba, BSc, PhD
Behavioral Scientist/ Post-doctoral Fellow
UNC Project-Malawi
Tidziwe Centre,
Private Bag A-104
Lilongwe, Malawi
Phone (Malawi): +265-1-755-056
Email: czimba@unclilongwe.org

1.0 Abstract

In Malawi, and other resourced-constrained sub-Saharan Africa (SSA) countries with high HIV burden, several service delivery models have emerged to increase PMTCT care retention for HIV-positive mothers and their infants by strengthening connections between health facilities and their surrounding communities. Through this study, we will rigorously characterize three such models of "community-facility linkage (CFL)," and compare their impact against each other and the standard of care according to routinely collected health outcomes for mother-infant pairs (MIPs), including maternal retention in care and viral suppression, and infant HIV-free survival. Specific objectives are to: (1) establish a clear typology for CFL models by describing the main components of, and key stakeholder perspectives on, three such models in Malawi; (2) Describe MIP health outcomes in each model, and compare outcomes across models and versus the standard of care (SOC); and (2a) Examine associations between individual components of CFL models and MIP health outcomes, controlling for confounding. We will complete our specific objectives by conducting an efficient two-stage cohort study. In the first stage, we will ascertain individual-level outcomes for a retrospective cohort comprised of *all* mothers enrolled in the national PMTCT program from July 2016 through June 2017 across approximately 40 health facilities and their surrounding communities in 5 districts representative of Malawi (each health facility and the surrounding communities to which the facility is responsible denotes one "facility catchment area" or "study site"). In the second stage, we will employ an epidemiological sampling method to prospectively ascertain MIP vital status, maternal viral load, and infant HIV status. Applying this method, we will create a nested cohort of approximately 1,200 randomly selected MIPs from our full retrospective cohort, including those lost to follow-up who will be traced in the community. For these MIPs, we will ensure viral load testing for ART-treated mothers and infants/ young children, and DNA PCR or HIV rapid testing for infants/ young children, and revise outcomes estimates for the full cohort accordingly. Such a two-stage approach will enable us to efficiently make robust inferences about the comparative impact of different CFL service delivery models and their main components under routine programmatic conditions. Study findings will identify components of community-based MIP support that are associated with maternal care retention and infant HIV-free survival to refine and accelerate scale up of promising CFL practices in Malawi and SSA. It is envisioned that this study will also increase local capacity to conduct high-impact, HIV-related operational research to build upon our findings, and further accelerate Malawi's progress toward achieving national 90-90-90 targets.

2.0 Background and Justification

Despite the impressive maternal and child health gains generated by the Option B+ and test and start strategies in Malawi, including increased maternal ART access and decreased rates of vertical transmission, client attrition from the PMTCT care continuum threatens recent progress. As many as 15% of HIV-positive pregnant women do not start ART,[1] and another 31% become lost to follow-up within 12 months of treatment initiation.[2] Similarly, high loss to follow-up is seen among HIV-exposed infants under 12 months of age and young children 12 months of age and older (henceforth referred to as "HEIs"), with 21% and 36% out of care by 12 and 24 months of age, respectively.[2] Such mother-infant pair (MIP) drop-out from the PMTCT continuum has deleterious "downstream" effects, as failure to deliver quality PMTCT services—including HIV diagnosis, treatment, and care for mothers and infants—leads to higher vertical transmission rates and worse clinical outcomes for HIV-positive mothers and HEIs alike.[3] Taken together, the effects of client loss from the PMTCT continuum may undermine Malawi's progress toward ambitious UNAIDS-endorsed 90-90-90 targets.[4]

To improve MIP care retention in Malawi, several models have emerged to strengthen community-facility linkage (CFL), a concept defined as any "formalized connection between a health facility and the communities it serves to support improved health outcomes".[5] Similar to other SSA settings, three models have been widely implemented to complement Malawi's National PMTCT Programme and enhance the <u>standard of care</u> (SOC). Under the current SOC, Ministry of Health (MOH) Health Surveillance Assistants (HSAs), a cadre that moves between clinics and decentralized "health posts" to enact a broad array of public health activities, support PMTCT clients primarily through health talks and tracing of MIPs who have defaulted from HIV care. However, HSA staffing levels are often insufficient to meet needs in the community (approximately 1 HSA per 1,000 households), and the resources and person-time available to HSAs to support dedicated PMTCT activities has historically been limited. In contrast, the three most common CFL models—1) mentor mothers; 2) expert clients; and 3) community health workers (CHWs)[6]—mostly or exclusively have a PMTCT focus and offer intensive psychosocial support to PMTCT clients. Mentor and expert clients are types of peer health workers—women living with HIV who offer longitudinal health education and psychosocial support, often via peer group meetings, to PMTCT clients.[7] CHWs are community-based health workers who provide clients with psychosocial support, promote medication and appointment attendance, accompany clients to health facilities for clinic visits, and support the services provided by other, often facility-based, health

workers.[7] All three models mobilize their respective CFL "provider"—i.e. the mentor mother, expert clients, or CHW—to trace HIV-positive women and HEIs who fall out of care, and encourage these women and infants to return to the clinic by phone contact or by targeted home visits in the community.

While the rationale underpinning these models has been supported by evidence generated from general ART and PMTCT programs across SSA,[6-8] a rigorous description of each model, including their unique and shared characteristics, and the extent to which the models are implemented with fidelity to their design, is currently unavailable. Moreover, a clear understanding of each model's major activities and defining features, including levels of CFL provider remuneration, supervisory structures, provider-to-client ratios, and relationships with clients and the formal health system, is similarly lacking. Finally, how and to what extent these components, both individually and collectively as part of CFL models, impact MIP care retention and joint health outcomes has not been well characterized, particularly in the era of test and start and against UNAIDS established 90-90-90 benchmarks.

3.0 Literature Review

Employing a public health approach to PMTCT, the Option B+ strategy of universal test and treat for pregnant and breastfeeding women has saved countless maternal lives and averted thousands of paediatric HIV infections in Malawi and elsewhere in SSA.[9, 10] In just its first year of implementation in Malawi, Option B+ increased maternal ART uptake by over 700%.[1, 11] However, despite these laudable achievements, sustaining mothers on ART and ensuring that they and their infants receive the full benefits of retention along the PMTCT care continuum remains a major challenge.

Interrupting the PMTCT care continuum at multiple points are a variety of psychosocial, health system, and structural barriers. These barriers encompass lack of social support,[12] fragmented social networks,[13] gender inequality,[14] infrequent male partner involvement,[15] internalized and enacted HIV stigma,[13, 15, 16] high travel and opportunity costs for accessing facility-based HIV services,[17] and limited counselling to facilitate acceptance of a new HIV diagnosis,[15] among other barriers.[18]

To mitigate these barriers, new and evolving strategies have been proposed that deploy lay health workers and peer cadres from the community to engage MIPs and bridge service offerings between communities and facilities. For PMTCT programs, these lay cadres have traditionally included community health workers (CHWs), mentor mothers, and expert clients, each of whom engage MIPs at community and/or facility levels to provide psychosocial support along the PMTCT care continuum.[19] While data regarding the impact of these cadres are still emerging, programs involving lay health workers have demonstrated early promise for improving maternal HIV prevention knowledge and uptake of such PMTCT services as HIV testing & counselling (HTC), ART, and early infant HIV diagnosis (EID).[19]

CHWs are a trained cadre of lay health worker that promote public health, link clinics and communities through referrals and home visits, and enhance uptake and delivery of health services.[18] CHWs may facilitate or directly provide a host of health services in response to the clinical and public health issues faced by their communities, ranging from counselling on maternal health and breast-feeding, supporting persons with mental illness, managing malaria and other uncomplicated paediatric diseases, and providing health education for chronic conditions such as HIV/AIDS, tuberculosis, and non-communicable diseases, among others.[20] How CHWs are organized can vary widely based on their employer (i.e. government versus civil society), training and supervision, and compensation and opportunities for career progression.[18] In SSA, CHWs have been deployed in numerous HIV care and ART programmes in a variety of country contexts. In Zambia, peer CHWs have been trained to provide home-based support and counselling for ART adherence, resulting in greater task shifting of health care worker responsibilities and decreased loss to follow-up in the national HIV programme.[21] In Rwanda, a community-based HIV treatment programme providing comprehensive psychosocial support to PLHIV on ART, including longitudinal accompaniment offered by CHWs, resulted in 92% programme retention at 24 months post-ART initiation.[22]

In Malawi, lay CHWs have been involved in a number of community-based care and treatment programs, including for palliative care, HIV-associated malignancies, and ART/ PMTCT, among others.[8, 23, 24] For PMTCT, the *Tingathe* program provides a modest work-related stipend to CHWs who support HIV-infected pregnant women and their children from the time of their first ANC visit through

breastfeeding cessation or successful ART initiation for infants diagnosed with HIV infection.[8] Using this approach, the *Tingathe* program has catalysed improvements in ART uptake (and uptake of other ARV prophylactic regimens prior to Option B+ introduction), facility-based delivery, infant PMTCT prophylaxis, and EID testing.[8] Similarly, Partners In Health—Malawi/ Abwenzi Pa Za Umoyo (APZU), established a CHW program in 2006 to support primary care service delivery in rural Neno District. The APZU program employs over 700 trained CHWs district-wide who serve as health educators, accompany patients with HIV, TB and other maladies to health facilities, and strengthen linkages for patients to the formal health system, including for PMTCT services.[25]

Mentor mothers are mothers living with HIV who are employed, trained, and supervised to support health workers in under-resourced health facilities to deliver services as part of the health care team.[6] The impact of mentor mothers has been reported previously from PMTCT programs throughout SSA. In Zimbabwe, a MSF-managed program demonstrated that clients receiving mentor mother support were twice as likely to return with their infants for EID testing compared to unsupported clients.[26] Clients interviewed in this program reported decreased stigma, greater empowerment, and increased agency to disclose their HIV status to their male partner(s) and successfully negotiate condom use.[26] In Uganda, the mentor mother model improved 12-month ART retention for HIV-positive mothers at mothers2mothers-supported facilities compared to clinics without mothers2mothers (m2m) services (90.9% vs. 63.6%, respectively).[27] Assessment of MTCT at the end of breastfeeding demonstrated a durable impact to the m2m model, with the rate of HIV acquisition among 18-month-old infants being 6.8% in m2m-supported facilities compared to 8.7% in facilities without m2m.[27] In South Africa, the presence of mentor mothers yielded favourable results on process indicators for HIV status disclosure, clinic attendance, and safe breastfeeding practices.[6] In Malawi, m2m manages mentor mothers in over 100 clinics nationally who lead group and individual counselling sessions to provide psychosocial support and health education to new mothers and pregnant women living with HIV.[6]

Expert clients are an emerging cadre who, like mentor mothers, are women living with HIV and who have experience navigating the PMTCT care continuum. However, expert clients typically have functioned on a volunteer basis with a strong community focus to meet a broad service delivery mandate, undertaking tasks ranging from providing psychosocial support and leading community-

based support groups,[28] offering facility-based counselling and vital sign measurement,[1] and tracing HIV-infected women who disengage from care in their communities.

PMTCT programs involving CHWs, mentor mothers, and expert clients each take different approaches to engage MIPs, link facilities and communities, and reach populations of women who may not otherwise seek PMTCT services.[19] Yet despite their promise, limited evidence rigorously describes these models, and the impact they and their components have on important joint PMTCT outcomes for mothers and their infants. Indeed, the impact of these CFL models, and the influence of the cadres who staff them, likely depends on a range of previously under-described variables, including levels of remuneration, frequency of client contacts, staffing ratios, supervisory structures and training, and relationships with clients and the formal health system.[19] To bridge these knowledge gaps, identify the essential elements of CFL models, and, ultimately, improve combined outcomes for HIV-infected pregnant women and their infants in an integrated PMTCT continuum, rigorous impact evaluation and characterization of CFL models is urgently needed.[19, 29]

4.0 Central Hypothesis

Our central hypothesis is that all three CFL models will be associated with increased MIP care retention, maternal viral suppression, and infant 12-month HIV-free survival compared to the standard of care, and that a CFL model featuring CHWs will be shown to be most effective, being associated with a higher proportion of HIV-positive women virologically suppressed and retained in care at 12 months, and infants alive, HIV-free and retained in care at age 12 months compared to other models. We further hypothesize that the following components of CFL models will be significantly associated with MIP retention in care and will constitute the most essential features of effective CFL models: low ratio of clients to CFL provider, high frequency of CFL provider-client interaction (i.e. ≥1 time per month), early "defaulter tracing" activities, and availability of one-on-one support from CFL providers.

5.0 Goal and Objectives

5.1 Overall Research Goal

To characterize widely adopted community-facility linkage models and rigorously assess the impact of the model and their components on MIP care retention and other priority maternal-infant health outcomes in Malawi.

5.2 Objectives

Our research objectives are:

1. To establish a clear typology for CFL models by describing the main components of, and key stakeholder experiences of and perspectives on, 3 common approaches in Malawi.
2. To describe MIP health outcomes in 3 CFL models, and compare outcomes across models and versus the standard of care using an epidemiologic sampling strategy.

    2a. To examine associations between individual components of CFL models and MIP health outcomes, controlling for confounding.

6.0 Methodology

6.1 Study Design Overview

We propose to rigorously define and compare three widely adopted models for community-facility linkage with each other and the standard of care using a mixed methods approach involving both quantitative and qualitative data collection.

For the quantitative component, we will first use stratified random sampling to select facility catchment areas in 5 Malawi districts where implementing partners have focused PMTCT programming. We will then visit each selected area to construct a retrospective cohort of HIV-infected women who entered the national PMTCT program and received CFL services or the SOC between July 1, 2016 and June 30, 2017. These dates have been chosen to align with the latest change to Malawi HIV guidelines supporting universal "test and treat" strategy, which became national policy on April 1, 2016 and reached stable rolled out on July 1, 2016. Using existing data, we will gather individual-level retrospective data on each woman and her infant from PMTCT enrolment through the child's second birthday. Next, we will enrich these data using an epidemiological sampling method designed to overcome the limitations traditionally associated with retrospective data (e.g. missing data, outcome misclassification, etc.). In this second stage, we will randomly select a sample of women from our full

cohort to create our nested cohort. These women will complete a questionnaire about their experiences with CFL models and services, undergo viral load testing, and have their children ages 12–24 months receive HIV testing. Such prospective data collection will enable us to confirm primary outcomes, fill in missing data, and improve study data quality more generally.

For the qualitative component, we will conduct both rapid and in-depth site assessments. Rapid site assessments will encompass: 1) a structured CFL model survey; and 2) structured observations of clinic visits and of client interactions with expert clients, mentor mothers, CHWs, HSAs, and other MOH healthcare workers (HCWs) in the three models plus the SOC. In-depth site assessments will require CFL model surveys and structured observations as done with rapid assessments, but will additionally incorporate 3) focus group discussions (FGDs) with CFL providers and MOH HCWs; and 4) in-depth interviews (IDIs) with purposively selected key stakeholders, including members of District Health Management Teams (DHMT) and supervisors/ managers of CFL programmes. A final qualitative data collection activity will be IDIs with HIV-positive mothers from our nested cohort.

Data from the qualitative component will be used to achieve objective 1; data from the quantitative component will be used to realize objective 2; and data from both the qualitative and quantitative components will be used to complete objective 2a.

6.2 Study Setting

The study setting includes 5 districts representative of Malawi, with a diversity of partner- and government-supported PMTCT programming. The 5 districts, encompassing Salima, Lilongwe, Neno Zomba, and Mzimba North/South (considered as one district for the purposes of this study given their longstanding history as a single district until very recently), were purposively selected to reflect the breadth of Malawi's demography—Neno, Salima, and Mzimba North/South are largely rural; Zomba is predominantly rural with a large peri-urban area situated in central Zomba town; and Lilongwe has a large, central urban pocket surrounded by peri-urban and rural locales.

6.3 Study Population

We will begin our study by reviewing existing data from a generalizable sample of women (and their infants/ young children) from selected facilities in the 5 districts meeting the following eligibility criteria:

- Newly diagnosed or having documented evidence of HIV infection between July 1, 2016 and June 30, 2017 in selected health facilities;
- Documented referral to or enrolment in Malawi's PMTCT/ ART program;
- Pregnant (at any gestational age) or breastfeeding at the time of HIV diagnosis/ positive HIV status identification;
- ≥16 years of age at the time of PMTCT referral/ enrolment (includes adults and emancipated minors according to Malawi law);
- Received Antenatal or PMTCT services at a facility catchment area served by a CFL model of interest or SOC.

Existing, routine data for these women and their infants will form the basis for our full retrospective cohort. From these data, we will randomly select women and their infants (i.e. mother-infant pairs) to participate in the nested cohort.

6.4 Sample Size

After enumerating the full retrospective cohort, we will randomly select eligible HIV-positive women and their HEIs for our nested cohort. For this nested cohort, our sample size will be determined by a power calculation for our primary outcome of interest—maternal retention in care 12 months after ART initiation. For this calculation (*see summary in* **Table 2** *below*), we assume that 75% of MIPs receive services from a CFL model of interest in our selected districts, and that approximately 73% of HIV-positive mothers receiving the SOC will be retained in care at 12 months post-ART initiation (based on the most recent, first quarter 2017 data from the Malawi national program). With a two-sided alpha of 0.05, we will have 80% power to detect a programmatically meaningful 8% difference in the proportion of mothers retained in care between those who do and do not receive CFL services by enrolling 894 mothers from CFL sites and 298 mothers from SOC sites into our nested cohort (for a total of 1,192 mothers). Assuming that approximately 75% of HIV-positive mothers in SOC sites will be virologically suppressed (a weighted composite of 88% suppression among the 73% of HIV-positive mothers in care[2] and approximately 40% suppression among the 27% of HIV-positive mothers not in care),[31] we will have 83% power to detect a 8% difference in the proportion of HIV-suppressed mothers between those who do and do not receive CFL services. Finally, assuming that we will enrol about one HEI for every mother in the nested cohort, and that 92% of all HEIs not receiving CFL services (i.e. receiving SOC) are alive and HIV-free at 12 months (based on published data that 7% of HEIs become HIV-infected by 12-months[9] and 96% of HIV-exposed but *uninfected* infants are alive at 12 months),[30] we will have 90%

power to detect a 5% difference in HIV-free survival between HEIs who do and do not receive CFL models of interest by recruiting 1,192 total HEIs..

Table 2: Summary of power and sample size calculations for primary outcomes of interest.

Outcome	Estimated % in SOC sites	Effect Size	Power	Sample Size in CFL Sites	Sample Size in SOC sites
Maternal 12-month retention in care	73%	8%	0.80	894 mothers	298 mothers
Maternal viral suppression	75%	8%	0.80	894 mothers	298 mothers
Infant 12-month HIV-free survival	92%	5%	0.90	894 infants	298 infants

For our qualitative and mixed methods study procedures, we have not calculated sample sizes *a priori*, as we will not be conducting statistical hypothesis testing. For IDIs with HIV-positive PMTCT clients, we will select about 60 women across all 5 districts from the approximately 1,200 mother-infant pairs in our nested cohort. For other study activities, we anticipate enrolling the following additional numbers (see section 6.5 below for further details): 1) up to about 8 facility and department in-charges (or designees) and CFL program managers from each of our 20 facility catchment areas to serve as key informants for completing CFL model surveys; 2) about 20 HIV-positive PMTCT clients for private structured observations; 3) a total sample of between about 120 and 240 participants to conduct 30 FGDs with MOH healthcare workers and CFL providers (recruiting a minimum of 4 and maximum of 8 participants per focus group); 4) approximately 10 IDIs with CFL programme site-level supervisors; 5) approximately 5 IDIs with DHMT members (1 per district); and 6) about 5 to 10 IDIs with CFL programme regional/ national managers/ leaders.

6.4.1 Sampling Approach for Facility Catchment Areas

Across the 5 study districts, we have identified the characteristics of interest for sampling facility catchment areas to be the district and CFL model (or SOC). To recruit our study population, we will first enumerate all high-volume health facility catchment areas within these districts, where “high volume” is defined as ≥30 pregnant women newly diagnosed with HIV infection in the catchment area within the last year. We will confirm the CFL model of interest/ SOC active in each facility catchment area prior

to data collection during the study start up phase through consultation with CFL programme managers /directors and District Health Offices (DHOs).

We will then stratify all facility catchment areas according to both district (i.e. 5 districts) *and* CFL model/ SOC (i.e. 4 total models) to create 20 "joint strata". Within each district, we will randomly select a fixed proportion (ranging between 66% and 100%) of high-volume facility catchment areas to identify approximately **40 facility catchment areas** that will serve as study sites. Table 1 provides an example of our approach to sampling community facility catchment areas within the districts of interest. A minimum of two facility catchment areas ("clusters") per stratum will be needed to quantify the within and between cluster variability in outcomes of interest (i.e. "rate of homogeneity"), and to provide overall variance and precision estimates. This sampling strategy serves as the starting point for recruiting the study population.

Table 1: Illustrative sampling approach for study sites.

**District**	**CFL Model** (SOC=0, Mentor Mother=1, Expert client=2, CHW=3)	**Joint stratum number**	**Estimated Number of Sampled Facility Catchment Areas**
1 (Salima, total 6 high-volume sites, 100% sampled)	0	1	0
1 (Salima)	1	2	0
1 (Salima)	2	3	0
1 (Salima)	3	4	6
2 (Lilongwe, total 12 high-volume sites, 75% sampled)	0	5	2
2 (Lilongwe)	1	6	3
2 (Lilongwe)	2	7	2
2 (Lilongwe)	3	8	2
3 (Mzimba North/ South, total 7 high-volume sites, 100% sampled)	0	9	2
3 (Mzimba North/South)	1	10	4
3 (Mzimba North/South)	2	11	2
3 (Mzimba North/ South)	3	12	0

4 (Neno, total 5 high-volume* sites, 100% sampled)	0	13	0
4 (Neno)	1	14	0
4 (Neno)	2	15	0
4 (Neno)	3	16	5
5 (Zomba, total 16 high-volume sites, 81% sampled)	0	17	2
5 (Zomba)	1	18	3
5 (Zomba)	2	19	6
5 (Zomba)	3	20	2
**TOTAL**		**20**	**40**
*Due to the highly rural and dispersed population in Neno, "high-volume" will be more than 10 pregnant women newly diagnosed in the catchment area within the past 1 year.			

As described in further detail below (see section 6.5.1), CFL site assessments will be conducted at about 20 purposively selected facility catchment areas where MOH PMTCT services are available and where one of the three CFL models of interest, or the SOC, are being offered.

To successfully meet our nested cohort enrolment target (accounting for missing locator information, maternal death, and other enrolment barriers), we will randomly sample at least 30 HIV-positive mothers per site from a fully enumerated list of all HIV-positive women meeting eligibility criteria, and seek to recruit a final nested sample of approximately 1,200 HIV-positive mothers and their HEIs (~30 MIPs per site x ~40 sites) from across all study sites. Based on Malawi HIV Unit program data, we anticipate that about 30 randomly selected HIV-positive mothers per site will represent a sample of between approximately 15% to 100% of all HIV-positive mothers newly enrolled into PMTCT during the full cohort period at each site, depending on PMTCT client volumes at selected facility catchment areas. This nested cohort sample size will not only allow for site-specific revised estimates of patient outcomes, but will also contribute site-level data to generate overall estimates for the full cohort for each CFL model.

To arrive at projected weights for each observation in our sampling scheme—which vary across joint strata—we have made the following assumptions: 1) at the *time of construction of the full MIP cohort*, we anticipate that approximately 73% of HIV-infected mothers will be alive and in care, with 4% documented as having transferred out, stopped ART, or died, and another 23% documented as lost to

follow-up (LTFU) to the national PMTCT/ART program (defined by the MOH as all those who have not returned to the clinic for more than 2 months after the date when they were expected to run out of antiretrovirals and are not documented as having transferred out, stopped ART, or died); 2) mortality is 30% among HIV-infected mothers who are LTFU; and 3) a coefficient of variation of 0.15 should be adequate to obtain relatively conservative projections of precision in each joint stratum. Applying these considerations to our MIP target population, we will select between 66% and 100% of high-volume sites and about 30 MIPs at each site from across the 20 joint strata to enrol our nested cohort (**Table 3**).

Table 3: Illustrative sampling approach for individual mother-infant pairs at facility catchment area level.

**Joint stratum number**	**Total high-volume catchment areas in strata**	**Expected catchment areas sampled (66 - 100%)**	***Expected Range* of number of PMTCT mothers available for sampling per joint stratum**	**Approximate Total Mothers sampled**	**Approximate Mothers with complete routine care data (.73 SOC, .80 CFLs)**	**Approximate Mothers Needing Tracing (0.27 SOC, 0.20 CFLs)**
1*	0	0	0	0	0	0
2	0	0	0	0	0	0
3	0	0	0	0	0	0
4	6	6	300 - 400	180	144	36
5*	2	2	80 – 100	60	44	16
6	4	3	400 – 500	90	66	24
7	2	2	150 – 200	60	48	12
8	2	2	60 – 100	60	48	12
9*	2	2	90 – 120	60	44	16
10	4	4	100 – 200	150#	120	30
11	2	2	300 – 400	90#	72	18
12	0	0	0	0	0	0
13*	0	0	0	0	0	0
14	0	0	0	0	0	0
15	0	0	0	0	0	0
16	5	5	50 – 100	90^	72	18
17*	2	2	60 – 100	60	44	16
18	3	2	150 – 200	60	48	12
19	9	6	900 – 1,100	180	144	36
20	2	2	100 – 200	60	48	12
**Total**	**45**	**40**	**2,740 – 3,720**	**1,200**	**942**	**258**

**Denotes SOC stratum (see Table 1 above)*
#Denotes strata where over-sampling will take place to achieve balance across strata
^Lower estimated number of mothers sampled given highly rural population (see Table 1 above)

6.5 Study Procedures by Objective

As part of this study, no new drugs, biological agents, or investigational devices will be used. Any and all illegal, reportable activities and adverse events will be reported to Malawian authorities according to national guidelines and in line with standard practices and routine clinical care.

6.5.1 Study Procedures for Objective 1

6.5.1.1 Objective 1 Overview

A clear description of expert client, mentor mother, and CHW CFL models and a rigorous evaluation of their impact on integrated health outcomes for HIV-positive pregnant and breastfeeding mothers and their infants have not been conducted previously under routine program conditions in Malawi. To characterize these models, a dedicated study team will conduct approximately **20 total site assessments** at about 20 facility catchment areas purposively selected from among the approximately 40 study catchment areas to capture a representative sample of sites in our 5 selected districts reflecting a diversity of demography (i.e. peri-urban versus rural area), CFL/SOC models, and health system dimensions (e.g. district hospital versus primary health center). The 20 total site assessments will be comprised of: approximately 10 rapid site assessments (about 2 per district) and approximately 10 in-depth site assessments (about 2 per district).

Rapid site assessments will involve: 1) conducting a CFL model survey and 2) completing a structured field observation. **In-depth site assessments** will involve the same CFL model survey and structured field observation as done with rapid site assessments, but will include additional qualitative data from 3) FGDs with frontline MOH HCWs and CFL providers; and 4) IDIs with CFL site-level supervisors. While more than one model may be available in the same facility catchment area, we will assess the extent of model overlap prior to sampling for in-depth site assessments. We will add district- and regional/ national-level perspectives to site assessments by conducting IDIs with DHMT officials involved with PMTCT activities and CFL programme managers operating in the district, or regionally/ nationally where appropriate.

6.5.1.2 Site Assessment Overview

During both rapid and in-depth site assessments, study staff will complete a CFL model survey to characterize the CFL model(s) available, CFL provider characteristics and activities (including the enablers and incentives, training and supervision, and job aides available to CFL providers), the health system tier, facility infrastructure, resources, and basic demography (i.e. rural versus urban/ peri-urban

setting), and the specific services offered, among other variables. Similarly, for both rapid and in-depth site assessments, study staff will conduct structured observations of clinic visits and of client interactions with expert clients, mentor mothers, CHWs, and healthcare workers (HCWs) in the three types of models plus the SOC.

For the 10 in-depth site assessments only, focus group discussions (FGDs) will be conducted with groups of professional and lay MOH health workers separately, as well as with CFL providers. In addition, in-depth site assessments will include IDIs with CFL site-level supervisors (or similar). In each of the 5 study districts, we will additionally interview a representative from the DHMT whose role is relevant to PMTCT and CFL coordination. We will also interview district-level CFL programme managers (or similar), or a member of CFL programme leadership operating at regional or national level should CFL programme management be based outside the district. Taken together, these data will provide both frontline and managerial perspectives on CFL and SOC activities and rich contextual information about cross-cutting issues of relevance to all facility catchment areas.

6.5.1.2.1 Community-Facility Linkage Model Survey

6.5.1.2.1.1 Sampling for CFL Model Surveys

Our dedicated, trained study team will complete a pre-coded, structured CFL model survey with the assistance of up to about 8 key informants at each of approximately 20 purposively selected sites, including the CFL programme site supervisor (or designee) for the catchment area plus the MOH clinic and departmental in-charges responsible at the facility.

6.5.1.2.1.2 Eligibility and Recruitment for CFL Model Surveys

Key informants assisting in the completion of the CFL Model Survey will be eligible to participate in the survey if they meet the following inclusion criteria: are ≥18 years of age; serve as CFL model site supervisor (or similar) or MOH facility/ clinic department in-charge (or designees); and are generally familiar with HIV and/or health services available in the facility catchment area. We will exclude any key informant if they are unwilling or unable to provide verbal informed consent in English. A trained member of study staff will identify and approach potential key informants in person and ask if they would be willing to help complete the CFL model survey. We will recruit key informants during breaks, lunch, and other off hours, and offer to conduct the survey at a convenient time of their choosing, so as not to take clinic and program staff away from their important duties. Key informants who support

CFL survey completion during their off hours or other break will receive a nominal lunch allowance (in the amount of $2–4 USD) in accordance with local MOH policy.

6.5.1.2.1.3 Consent Procedures for CFL Model Surveys

All potential key informants will undergo verbal informed consent by a trained member of the study team. The study procedures, risks, and benefits will be discussed in detail. The key informant will have the opportunity to ask questions and deliberate before they decide whether or not they consent to participate. Verbal informed consent procedures will be performed in English, as the informants we aim to recruit are all conversant in English. An information sheet will be made available to the key informants. Study staff will explain to the potential key informants that their participation will in no way affect their employment or standing with their employer. As the survey will be brief, conducted at or near the site where key informants are employed, and will be done during a break or other off hours, no financial reimbursement other than the standard MOH lunch allowance will be provided to key informants.

6.5.1.2.1.4 Data Collection for CFL Model Surveys

The survey is structured to characterize CFL components as well as facility and health system characteristics, and includes items from the WHO Service Availability and Readiness Assessment (SARA) tool.[32] A member of the study team will implement the survey together with consenting key informants. Responses will be collected through clinic and program walk-throughs and by administering questions to key informants. After obtaining verbal informed consent, the study staff member will read the relevant survey items and all response options aloud to the key informant in English, and write down their responses on a paper-based survey form. The survey will take approximately 2 hours total to complete at each study site, with each key informant being asked to spend between 10 and 60 minutes answering questions related to their area of expertise. The survey does not include any questions intended to obtain health-related or other personal information from key informants. Rather, the survey will rigorously describe CFL model components and context, on such variables as: 1) CFL provider factors like training and supervision, counselling and health education responsibilities, defaulter tracing and patient follow-up activities, use of mHealth and other job aides, peer support group facilitation, and remuneration packages (including monetary and non-monetary incentives), among other variables; 2) Facility-level factors that may influence CFL activities, such as health worker staffing levels, infrastructure and health commodity availability, and provision of HIV

testing services (HTS), ANC, PMTCT, ART, child health and laboratory services, among other variables; 3) Socio-demographic factors for facility catchment areas (drawn largely from demographic and health surveys and district socio-economic profiles), including education levels, infant mortality, and adult population HIV prevalence (de-aggregated by district/ zone); and 4) Presence and extent of CFL program and implementing partner overlap, among other variables.

We may pilot test the survey with approximately 5 to 10 UNC PMTCT project managers and MOH facility / department in-charges in Lilongwe prior to data collection to tailor the survey language to the local context and to ensure local acceptability and comprehension of all survey items.

6.5.1.2.2 Structured Field Observations

Structured observations acknowledge the importance that health system and CFL model performance play in MIP retention, and are intended to capture important data on health facility and CFL model operations, fidelity to CFL model guidelines and training materials, service quality and responsiveness and, importantly, patient-provider relations.

6.5.1.2.2.1 Sampling for Structured Field Observations

Structured observations will take place at the same 20 health facility catchment areas/ sites where the CFL model surveys will be conducted (including the approximately 10 sites selected for rapid site assessment and approximately 10 selected for in-depth site assessment).

6.5.1.2.2.2 Data Collection for Structured Field Observations

Field observations will consist of: directly observing encounters between MIPs and HCWs as well as between MIPs and expert clients, mentor mothers, and CHWs; reviewing facility and program registers and records; and generally observing the flow and dynamics of service delivery activities in facilities and the surrounding community. The data will be formalized into research memos and will contribute to building a picture—alongside data from CFL surveys and focus group discussions—of CFL/SOC service delivery quality, participant responsiveness to services offered, intensity or "dose" of psychosocial support provided, CFL model/SOC adherence to guidelines/ training materials, and the underlying mechanisms, workflows, and human interactions that drive CFL model/SOC operations.

Structured field observations will be divided into two categories: public observations and private observations. Public observations will involve a trained study team member as a passive observer in

public observable zones within health facilities, such as waiting areas, departmental registry rooms and vitals measurement stations, or at CFL program offices and meeting venues in communities. In all instances, permission to be based in these areas will be established with the overall-in charges (for facilities) and with CFL program supervisors (for communities) prior to the commencement of study activities. Informed consent will not be sought out for public observations. Based on a list of themes outlined in the semi-structured "Public Observation Activity" tool, the trained observer will sit or stand in an unobtrusive location to observe patient flow, one-on-one and group interactions involving HCWs and CFL providers, and other elements of day-to-day operations. Each 'block' of observation will last 1-2 hours. As far as possible, the observer will not participate in conversations or activities although where necessary may answer direct questions. Short hand notes will be recorded around general environment and workflow, CFL provider/ HCW service delivery behaviours, communication and rapport-building patterns, and patient, HCW and CFL provider group behaviours, among others. Notes will be transcribed in full at the end of the day (or as soon as possible thereafter), and formalized into a summary memo.

Dependent on the CFL model of interest, a *second* category of observations—private observations—may take place in more private settings, such as ANC/ART/HCC clinic examination rooms and HCT counselling rooms for facilities, and in clients' residences during CFL provider home visits or other community-based venues, such as support group meetings, for example. Observations will be conducted once or twice in each type of private setting, both in the facility and the surrounding community, and should take about 1 to 2 hours. During this time the trained observer will adopt a completely passive role and (following consent procedures) will not participate in conversations or service delivery or interact with either the patient or provider. Direct observations will be conducted and short hand notes collected on the following themes, among others: services provided; CFL/SOC service delivery quality; participant responsiveness to services offered; intensity or "dose" of psychosocial support provided; CFL model/SOC adherence to guidelines/ training materials; service delivery environment; patient, HCW, and CFL provider communication styles and rapport building; equipment and/or consumables used; and possible underlying mechanisms driving CFL model/SOC operations. Notes will be written up in full at the end of the day (or as soon as possible thereafter), and formalized into a summary memo.

6.5.1.2.2.3 Sampling for Private Field Observations

Sampling for patient participation in the private observations will be consecutive at each site, based on relevant clinical and CFL programmatic activities taking place at the site over the field observation period. We will seek to enrol approximately 20 HIV-infected women (or about 1 woman at each site undergoing a site assessment).

6.5.1.2.2.4 Eligibility and Recruitment for Private Field Observations

Patients will be eligible to participate in a private field observation if they meet the following inclusion criteria: are ≥16 years of age; have documented HIV infection; and have previously joined the national PMTCT programme in the facility catchment area. We will exclude any potential participant if they are unwilling or unable to provide verbal informed consent in a study language (i.e. Chichewa, Yawo, Tumbuka, or English). A trained member of study staff will identify and approach potential participants in person and ask if they would be willing to be part of the private field observation. We will recruit potential participants during the flow of routine clinical and CFL programmatic activities during the designated observation period. For all private observations, we will seek formal verbal informed consent from patients prior to commencing observations.

6.5.1.2.2.5 Consent Procedures for Structured Observations

Since the focus of structured observations relate to the scope and manner in which services are provided and no personal or otherwise identifying information will be sought or recorded about providers or patients, we are seeking a waiver of formal consent for public observations, and plan to seek verbal informed consent for patients participating in private observations. For all private observations, patients will be informed of the observers' presence and have the opportunity to decline participation. Whenever requested, the observer will exit the room, home, or other private setting for the duration of the encounter and then may re-enter prior to the next patient, as appropriate. All potential patient participants will undergo verbal informed consent by a trained member of the study team. The study procedures, risks, and benefits will be discussed in detail. The potential patient participant will have the opportunity to ask questions and deliberate before they decide whether or not they consent to undergo private observation. Verbal informed consent procedures will be performed in Chichewa, Yawo, Tumbuka, or English based on potential participant preference. An information sheet will be made available to all participants. Study staff will explain to potential participants that their decision to take part or not take part in the private observation will in no way

affect their healthcare or other services they receive.

6.5.1.2.2.6 Additional Procedures for Structured Field Observations

Observations will be conducted over approximately 2–3 days, incorporating time spent in facility departments, such as ANC, HIV Care (HCC) and ART clinics, HTC rooms, the maternity ward, and the laboratory, as well as the community venues where CFL providers and supervisors work. In facilities, the study observer(s) will introduce themselves to all HCWs and staff, accompanied by the facility in-charge, in a general round of introductions at the beginning of the observation-period, and subsequently sit in a single space (as much as possible) in departments of interest, making shorthand notes related to their observations of health facility operations, healthcare worker interactions (verbal and non-verbal), patient-provider interactions and informal conversations. In communities, the trained study observer(s) will introduce themselves to all CFL providers, accompanied by the CFL program supervisor, at the beginning of the observation period. Subsequently, observer(s) will accompany providers as they make home visits or conduct support group sessions and will take shorthand notes on any informal conversations, their interactions with clients, general community members, healthcare workers, other CFL providers, and overall impressions of relations and communication among clients, HCWs, and CFL providers. Notes will be structured under general thematic headings, including: operations; environment and workflow; communication patterns; and work patterns/ behaviours. Where possible, notes will be transcribed daily into an electronic log for later analysis.

6.5.1.3 Focus Group Discussions

6.5.1.3.1 Sampling for Focus Group Discussions (FGDs)

Focus groups will take place in approximately 10 sites selected for in-depth assessment. In selected sites (i.e. facility catchment areas), 3 FGDs will be conducted, giving approximately 30 for the study. These will target: 1) MOH healthcare workers including both professional (e.g. COs/Nurses) and lay (e.g. HSAs) healthcare workers; and 2) CFL providers (e.g. mentor mothers, expert clients, community health workers, etc.). Sampling for participation in FGDs at each site will be opportunistic based on an open invitation to all relevant (e.g. ANC, ART, CFL programme, etc.) staff in and around the facility.

6.5.1.3.2 Eligibility and Recruitment for FGDs

FGD participants must be: ≥18 years of age; an expert client, mentor mother, CHW, other type of CFL provider, or frontline MOH HCW involved with ANC/PMTCT/EID service delivery; and generally familiar with PMTCT/ HIV services in their district. We will exclude any potential FGD participant if they are unwilling or unable to provide verbal informed consent. For pragmatic reasons, including the need to minimise the perception of special treatment, sampling will be opportunistic and based on a first come, first served basis until the target group number (maximum of 8) are met. To minimize expectancy bias, a standard invitation letter will be sent initially to facility in-charges, requesting planning assistance, and then to individual potential participants, as appropriate, explaining the nature and purpose of the FGDs. The study team will work with the in-charge and CFL programme site supervisors to schedule a meeting with the relevant MOH HCWs and CFL providers, respectively, to introduce the study activities and invite participants to join the FGDs at specified times convenient to the HCWs and CFL providers.

6.5.1.3.2.1 Consent Procedures for FGDs

Participation in the FGDs will be completely voluntary and subject to verbal informed consent. While we anticipate that the majority of potential FGD participants, particularly HCWs, will prefer to undergo consent procedures in English, we will offer consent in the participant's choice of English, Chichewa, Yawo, or Tumbuka. Moderators will make it clear that participants do not need to answer questions that make them feel uncomfortable. All those taking part in FGDs will receive the Malawi Kwacha equivalent of $4, and a snack and refreshment for their participation.

6.5.1.3.3 Data Collection for FGDs

A small team of trained study FGD moderators will conduct all FGDs using semi-structured discussion guides that will explore general domains of feasibility and implementability, including: *acceptability* (the extent to which CFL models are perceived as suitable or satisfying to clients); *demand* (the extent to which CFL models are used by clients); *implementation* (the extent to which CFL models are delivered according to program objectives and work plans in challenging field contexts and offer comprehensive services); *practicality* (how are CFL models managed, and to what extent do CFL models complement existing systems and circumstances in MOH programs and clinics); *adaption* (the extent to which CFLs model function in different settings like clinics, communities, homes, etc.); *integration* (the ease with which CFLs model are integrated within the existing health system); and *expansion* (the perceived ease with which a CFL model, or its components, can be scaled up or become owned by government). Within

this thematic framework, questions will be open-ended to allow the moderator to probe for emerging and minor themes. The discussion guides will include a standard opening statement for use by the moderator to clarify FGD processes and participant roles, and will be structured such that participants are encouraged to respond mostly to the moderators and not one another. FGDs will last approximately 1 to 2 hours each. All FGDs will be audio-recorded (due to the impracticality of taking sufficiently detailed notes during a multi-participant discussion). FGDs for expert clients, mentor mothers, community-health workers, and HSAs may be conducted in Chichewa, Yawo, or Tumbuka, as appropriate and based on participant preference, and then transcribed verbatim and translated into English in a single step by trained study staff. All other FGDs will be conducted in English. Participants will be informed that they may use a participant number or pseudonym for the purposes of identifying themselves during FGDs. Those who do not wish to have their voice recorded at all will be ineligible to participate. The audio-recordings will not be marked with any identifying information. Digital audio-recordings will be stored on a password-protected computer, accessible only to trained study staff. After transcription, audio recording will be stored for a maximum of 2 years and then destroyed.

6.5.1.3.4 Additional Procedures for FGDs

Moderators will be trained and mentored by the study investigators on FGD procedures, FGD standard operating procedures (SOPs), and standardized discussion guides. During FGDs, moderators will monitor group dynamics to minimize dominance and shyness bias.

6.5.1.4 Managerial In-Depth Interviews

6.5.1.4.1 Sampling for Managerial In-Depth Interviews (IDIs)

IDIs will take place with CFL site-level supervisors at approximately 10 sites selected for in-depth assessment, as well with DHMT members and CFL programme managers at district and regional/ national levels. In selected sites (i.e. facility catchment areas), 1 IDI will be conducted with a CFL site-level supervisor (or similar), giving approximately 10 for the study. At District level, 1 IDI will be conducted with a DHMT representative whose role is relevant to PMTCT and CFL coordination, giving approximately 5 total for the study. Also, whenever possible, we will interview a total of 5 to 10 CFL programme manager(s) at district level, or failing that, members of CFL programme leadership based at regional or national levels should management reside outside the districts. Sampling will be purposive and based on prior identification of individuals occupying relevant roles of interest.

6.5.1.4.2 Eligibility and Recruitment for Managerial IDIs

IDIs participants must be: ≥18 years of age; a DHMT member (or designee), a CFL site-level supervisor (or equivalent), or a district-, regional-, or national-level CFL programme manager (or similar); and generally familiar with PMTCT/ HIV services available in the relevant district(s). We will exclude any potential IDI participant if they are unwilling or unable to provide written informed consent. A standard invitation letter will be sent initially to potential participants, followed by a phone-call and/or in-person visit to explain the study. Where interested and available, a date and venue convenient to the participant will be identified.

6.5.1.4.2.1 Consent Procedures for Managerial IDIs

Participation in the IDIs will be completely voluntary and subject to written informed consent in English. Interviewers will make it clear that participants do not need to answer questions that make them feel uncomfortable. All those taking part in managerial IDIs will receive the Malawi Kwacha equivalent of $10, and a snack and/or refreshment for their participation.

6.5.1.4.3 Data Collection for Managerial IDIs

A small team of trained study interviewers will conduct all IDIs using semi-structured discussion guides that will explore general domains of feasibility and implementability, including: *implementation and fidelity* (the extent to which CFL models are delivered according to program objectives and work plans in challenging field contexts and offer comprehensive services); *practicality* (how are CFL models managed, and to what extent do CFL models complement or duplicate existing systems and activities with other partner health projects and MOH programs and clinics); *adaption* (the extent to which CFLs model function in different settings like clinics, communities, homes, etc.); *integration* (the ease with which CFLs model are integrated within the existing health system); and *expansion* (the perceived ease with which a CFL model, or its components, can be scaled up or become owned by government). Within this thematic framework, questions will be open-ended to allow the moderator to probe for emerging and minor themes. IDIs will last approximately 60 minutes each.

All IDIs will be audio-recorded and then transcribed verbatim. IDIs will be conducted in English given that participants are professional managerial staff. The audio-recordings will not be marked with any identifying information. Digital audio-recordings will be stored on a password-protected computer,

accessible only to trained study staff. After transcription, audio recording will be stored for a maximum of 2 years and then destroyed.

6.5.1.4.4 Additional Procedures for Managerial IDIs

Interviewers will be trained and mentored by the study investigators on IDI procedures, IDI standard operating procedures (SOPs), and use of standardized discussion guides.

6.5.2 Study Procedures for Objectives 2 & 2a

6.5.2.1 Objectives 2 & 2a Overview

In "real-world" PMTCT programmatic settings where the absolute numbers of MIPs is large, prospectively ascertaining clinical outcomes, including maternal viral load and infant/young child HIV status, is often challenging for an implementation research study such as this one. Therefore, we will learn about MIP health outcomes using an efficient, two-stage cohort design.

In the first stage, we will retrospectively construct a cohort of HIV-infected women entering the national PMTCT program and receiving community-facility linkage (CFL) services or the SOC between July 1, 2016 and June 30, 2017 in our 40 randomly selected facility catchment areas. For all areas, we will review existing, routine MOH data for all eligible women and their infants to abstract retrospective data on our outcomes of interest—12-month maternal retention in care, viral suppression for HIV-positive mothers, and 12-month infant HIV-free survival. (Of note, maternal and infant outcomes have two different "starting points" for determining the 12-month outcome, which we will account for in our analysis—in the case of HIV-positive mothers the "starting point" is ART initiation date and for infants it is date of birth).

In the second stage, we will use epidemiological sampling to obtain outcomes on a nested cohort of approximately 1,200 randomly selected MIPs drawn from the full retrospective cohort (*see section 6.4 Sample Size*). Information will be collected from these 1,200 participating MIPs through a detailed questionnaire and blood sampling for maternal viral load and infant HIV testing, and will be enriched by qualitative in-depth interview (IDI) data for 60 women purposively selected from among the MIPs in the nested cohort. IDIs will explore maternal perceptions, experiences, and satisfaction with CFL models and services, including with their key components, and how these services have affected their longitudinal engagement with and retention in the national ART program.

Data from the first and second stages will be combined to present the HIV care cascade for MIPs in aggregate, and by CFL model/ SOC, from HIV testing through ART initiation and viral suppression. Uptake of HIV services at the three main steps of the HIV care cascade will be calculated according to UNAIDS definitions and compared against established "90-90-90" benchmarks (*see section 7.2.2.2 below*).

6.5.2.2 First Stage Data Collection: Chart Review

In the first stage of data collection, we will seek out outcome information for all MIPs in the full retrospective cohort by abstracting existing clinical and demographic data from routine MOH treatment cards and registers and from electronic medical records (where available) at our 40 selected facility catchment areas. Study staff will have no direct contact with MIPs in the retrospective cohort, apart from those MIPs selected to potentially participate in the nested cohort.

Information to be abstracted will include such data as: HIV testing history, 6-, 12-, and 24-month post-ART initiation maternal vital status (e.g. alive in care, transferred out, stopped ART, died, and lost to follow-up); 6- and 24-month maternal viral load (timing per national guidelines); exposed-infant first HIV-1 DNA PCR result (from 6 weeks of age per national guidelines); 12- and 24-month HEI vital status (including death); and 12- and 24-month HEI HIV sero-status (timing per national guidelines). We will also abstract the first documented DNA PCR test result for all HEIs from HCC registers and treatment cards and laboratory records, which will include results for samples taken at 6 weeks of age and later, including "late" test results for HEIs who enrolled into follow-up after 6 weeks of age. Similarly, many HIV-exposed children undergo their 12- and 24-month rapid tests after these time points due to late presentation to clinic for "milestone" visits, HIV test kit stock outs, and other reasons. As a result, the "median age at first HIV rapid testing" may be closer to 15 or 18 months than the recommended 12 months, providing valuable information about HEI HIV status at or near the conclusion of breastfeeding. Because HEIs enrolling into routine HIV care through June 30, 2017 are eligible to join the full retrospective cohort, some HEIs will not reach age 24 months before administrative censoring (anticipated to take place between end 2017 and end 2018, depending on the district). The 12-month calendar period for cohort construction was selected to maximize power for the primary outcome (i.e. 12-month maternal HIV retention in care). 24-month outcomes for infants who are censored before reaching age 24 months will be represented by those who are observed at age 24 months and by a simple sensitivity analysis (*see section 7.0 Data Analysis*).

6.5.2.3 Second Stage Data Collection

6.5.2.3.1 Nested Cohort Procedures Overview

Randomly selected nested cohort MIPs will undergo written informed consent, complete a structured questionnaire about their experiences with CFL models and other health services, and undergo one-time HIV testing (i.e. by collecting a dried blood sample [DBS] from the mother for viral load testing and either performing rapid HIV-1/2 antibody testing or collecting a DBS sample on the HEI for HIV infection ascertainment, as indicated based on HEI age per national testing guidelines).

6.5.2.3.2 Selecting Patients for Potential Nested Cohort Participation

All women meeting eligibility criteria for the retrospective cohort will be eligible for random sampling and inclusion in the nested cohort, regardless of their ART status in the national program (i.e. regardless of whether they are known to be active and in care or defaulted/ lost to follow-up). At each study site, we will enumerate a complete list of study-eligible PMTCT clients from the full retrospective cohort in chronological order of the date of their PMTCT referral/ enrolment. For relatively small enumeration lists (e.g. containing fewer than approximately 50—70 clients), all clients will be included as potential participants. For larger enumeration lists, we will randomly select every second to n^{th} PMTCT client for potential participation in the nested cohort, with the interval depending on the total number of eligible mothers at the site from which to choose at least 30 participants (e.g. if there are 80 mothers available for sampling, we will select every second entry; if there are 200 mothers available for sampling we will select every fourth entry; etc.). We will continue this selection process until we enrol at least 30 nested cohort participants at each study site. It is possible that we are unable to complete study procedures (described in detail below) on the first MIPs selected at each site. In such a case, we will continue randomly sampling from the enumeration list until we reach at least 30 consenting MIPs who have completed study procedures in fulfilment of our site-based enrolment target.

6.5.2.3.3 Contacting Selected Potential Nested Cohort Participants

We will review treatment master cards for patients selected for potential participation to determine whether they are active and in care or whether they have defaulted / are lost to follow-up in the national program.

For selected potential participants who are currently in care, we will determine when their next scheduled ART appointment will take place, and if it is scheduled to occur within about 4 weeks of their selection, we will send them a SMS reminder shortly before their scheduled appointment to remind

them to come to the clinic together with their infant. The SMS content will be generic, brief, not mention HIV, and will not disclose any health or identifying information. The SMS will include a message to the effect of: “Please come to the clinic on (date) with your infant for important health information.” In areas where cellular network coverage is poor, or in cases where the available clinic records do not list a reachable phone number, a staff member may accompany selected CFL providers and/or HCWs already working in the community and/or assigned to that particular client to deliver this same message, or ask the CFL provider or HCW to provide the message themselves, during the course of a routine home visit or other routine programmatic encounter with the client. Working closely with CFL providers and facility health workers, we will then approach selected potential participants on the day they return to the facility for their appointment, to tell them about the study in a sensitization session and proceed with written informed consent procedures if they are interested in volunteering for the study.

For potential participants who are in care but have an appointment scheduled for approximately 4 weeks or more from the time of selection, or at a time when study staff will not be available at the site, study staff may attempt to contact them by telephone using contact information available in the clinic record or CFL program files. If phone contact is successful, study staff will use a phone script to briefly explain the study and ask if the potential participant would consider coming to the clinic at a convenient time within the next several weeks to be sensitized about the study. Failing a phone contact, or in areas where cellular network coverage is poor, study staff will ask selected CFL providers and/or HCWs already working in the community and assigned to that particular client to deliver this same message during the course of a routine home visit or other routine programmatic encounter with the client. Depending on the time frame for data collection and the availability of CFL providers and/or HCWs in the selected facility catchment area, study staff may work through established CFL or MOH tracing mechanisms to contact potential participants in person at their home or other location in the community and offer potential participants the option of coming to the clinic at an agreed-upon time to learn more about the study, or to proceed with study sensitization, informed consent, and nested cohort procedures in the community (following the approach detailed under section 6.5.2.3.8 below). Potential participants will be informed that they will be reimbursed for their time and/or transport. Potential participants will also be given the opportunity to refuse to proceed with study procedures or to decline to learn more about the study by coming to the clinic, should that be their preference. For

potential participants agreeing to come to the clinic to learn more about the study at a later time, we will send them a SMS reminder shortly before the agreed upon time to remind them to come to the clinic together with their infant. The SMS content will be generic, brief, not mention HIV, and will not disclose any health or identifying information. The SMS will include a message to the effect of: "As agreed, please come to the clinic on (date/ time) with your infant to learn more about the study."

For selected potential participants who have defaulted or are lost to follow up in the national program, study staff will attempt first to reach these women by phone and encourage them to return to the clinic to proceed with their routine HIV care, and will make an arrangement to meet with the potential participant after they complete routine clinic procedures on their next visit. Failing a successful phone contact, the potential participant will be sought in person using information available on the master card/ clinic file or known by HCWs or CFL providers, via study or public transportation, bicycle or on foot, as appropriate. Further details on tracing procedures are provided under section 6.5.2.3.8 below.

6.5.2.3.3.1 Consent Procedures for Nested Cohort Procedures

All nested cohort potential participants whether approached in the facility or the community, will undergo written informed consent by a trained study staff member. The study procedures, risks, and benefits will be discussed in detail. Potential participants will have the opportunity to ask questions and deliberate before they decide whether or not they wish to consent to participate. Written informed consent procedures will be performed in Chichewa, Yawo, Tumbuka, or English, based on participant preference. A copy of the informed consent form will be made available to all participants. Study staff will explain to all potential participants that their decision to join or not join the study will in no way affect their health care, or their child's healthcare, either at the health facility or in the community. As nested cohort procedures seek to enhance the current standard of care, and will be aligned with the schedule of existing clinic visits whenever possible, a financial reimbursement of the Malawi Kwacha equivalent of $4 will be provided only to participants who travelled to the clinic for study purposes and who consented to join the study.

6.5.2.3.4 Nested Cohort Structured Questionnaire

We will administer a brief paper- or tablet-based questionnaire during consenting participants' facility- or community-based study encounter to examine client attitudes, met needs, and unmet needs, as well as services offered, received, and preferred, relating to PMTCT and CFL models/ SOC. Needs assessed

through the questionnaire will focus principally on services intended to promote access to and retention along the PMTCT care continuum, including: HTC; ART; couples' counselling and testing; adherence and other psychosocial support; initial linkage to care, missed appointment, and "defaulter" follow-up; infant ARV prophylaxis; nutritional supplementation; and EID testing, among others. Examining access and retention needs will help document the landscape and hierarchies of needs that are currently being met, or not yet being met, by on-going CFL programs/ SOC. Such a focus will enable the study to identify CFL program components that are most useful or impactful to clients, as well as those that should be considered for inclusion in future CFL programs. To these ends, we will examine the following need dimensions for CFL services, including: *availability* (e.g. what, if any, services do PMTCT clients report receiving to help them navigate the PMTCT care continuum, including interventions to promote linkage to care and treatment retention, as well as address stigma and lack of male partner involvement); *physical and financial access to services* (e.g. distance, time, [direct and opportunity] costs, and other barriers to receiving such services at facilities and in the community); and *acceptability* (of specific services and who provides them). In addition, the questionnaire will assess individual-level attitudes and perceptions of CFL services focused on *satisfaction*, *empathy*, *activation*, and *respect*—each of which are established concepts that appear to play a role in HIV care engagement in sub-Saharan Africa.[33-35] Finally, the questionnaire will elicit an individualized CFL program history and examine other CFL-related elements from the client perspective, including: CFL program overlap within the same catchment areas and for the same clients; perceived appropriateness / usefulness of CFL services; psychosocial support and counselling needs following new HIV diagnoses; and experiences with re-engaging with PMTCT care and facility-based services after missing clinic appointments and/or defaulting from the ART program.

6.5.2.3.5 Nested Cohort Maternal and Infant Viral Load Ascertainment

Study staff will support routine HIV laboratory monitoring at MOH ART clinics in selected districts for maternal nested cohort participants. Consenting nested cohort participants on ART, including HIV-infected mothers and infants/ young children on treatment, will undergo blood sample collection for quantitative HIV-1 viral load (RNA PCR) testing according to national guidelines. Depending on the standard practice already in place at the facility, blood sampling may proceed by venipuncture (i.e. 10 ml of whole blood) or by finger prick for DBS collection (i.e. 1 DBS card containing 5 wells of approximately 50 µL each, or 250 µL total) for mothers and by DBS collection for infants/ young

children. For participants completing study procedures in the community, blood sampling will proceed by DBS collection. In all cases, we will follow MOH guidelines for the preparation, collection, handling and transport of DBS samples for VL testing.[36] If participants have not undergone MOH-recommended 6-month or 24-month viral load (VL) testing at the time of study consent, samples for “catch up” VL testing will be obtained by study staff for testing at the appropriate MOH-designated laboratory. If these participants have already complied with the recommended MOH VL testing schedule, but do not have a VL test result documented within 3 to 6 months or longer of study consent, study staff will collect a study-specific blood sample for VL testing by venipuncture (i.e. 10 ml of whole blood) or by heel/finger prick for DBS collection (i.e. 1 DBS card containing 5 wells of approximately 50 µL each, or 250 µL total) depending on the age of the participant (e.g. DBS collection for all infants/ young children), sample transportation logistics and participant preference, to be done at the UNC Project Tidziwe Laboratory in Lilongwe. While it is envisioned that most women and infants/ young children will receive VL testing at approximately 6 and 24 month post-ART initiation per national guidelines, VL testing may occur outside these time points depending on the point at which participants started ART, the timing of study field activities in selected districts, and whether or not the woman or infant/young child accessed routine VL testing through the national program. In cases where the HIV VL returns greater than 1,000 copies/ml, excess blood sample may be sent for HIV genotype/ resistance testing in selected cases to assess for the presence of HIV-1 resistance-conferring mutations, study resources permitting.

All VL test (and any genotype test) results will be returned to the clinic from which the patient was originally identified. We will subsequently inform all patients and mothers of patients that the test results are ready and have been submitted to the clinic. We will recommend they return to discuss these results with the clinic healthcare providers. This will support good clinical care and will also provide additional encouragement to those who have disengaged/ defaulted from care to re-engage.

6.5.2.3.6 Nested Cohort Exposed Infant Biological Outcome Ascertainment

For HEIs of maternal nested cohort participants, study staff will support routine EID testing procedures at MOH ART/HCC clinics in selected districts according to national guidelines.[36, 37] Thus, for HEIs over 6 weeks but under 12 months of age who are without a documented DNA PCR test result at the time of study field activities, we will collect a routine DBS sample for “catch up” DNA PCR testing to be performed through the existing MOH EID testing system. Similarly, for HEIs 12 months of age and older without a documented HIV rapid test result, we will support “catch up” infant rapid HIV serological

testing through review of existing MOH records and referral of HEIs with unknown status for HIV testing services (HTS).

For HEIs of nested cohort participants who are over 6 weeks but under 12 months of age, and who have previously undergone MOH EID testing but who are without a documented HIV test result within about 3 months of their mother's study encounter, we will collect 1 DBS card sample (i.e. 5 wells of 50 µL each or 250 µL total) following MOH-recommended collection procedures for study-specific DNA PCR testing to be done at the UNC Project Tidziwe Laboratory in Lilongwe. For HEIs 15 months of age or older who have not received routine HIV testing within about 3 months of their mother's study encounter, study staff will conduct study-specific HIV rapid antibody testing in accordance with MOH HTC procedures.[36, 37]

In all cases, we will follow MOH guidelines for HIV testing, including the preparation, collection, handling and transport of DBS samples for EID testing.[36, 37] All EID DNA PCR test results will be returned to the clinic from which the HEI was originally identified. We will subsequently inform all mothers that their HEI's test results are ready and have been submitted to the clinic. We will recommend they return to discuss these results with the clinic healthcare providers. This will support good clinical care and will also provide additional encouragement to those disengaged/ defaulted from care to re-engage.

Due to the dispersion of HEI ages at the time of maternal and infant enrolment in the nested cohort, it will not be possible to ensure testing at a pre-specified age. We will adjust for any variability in timing of HIV testing during the analysis phase (*see section 7.0 below*).

6.5.2.3.7 Routine Care and Treatment for Maternal and Infant Nested Cohort Participants

As we are proposing an observational study without intervention, routine HIV treatment and care for MIPs enrolled in the nested cohort will continue to be provided by MOH HCWs at MOH facilities according to standard clinical practice, in strict accordance with the Malawi Ministry of Health integrated PMTCT/ART guidelines.[36]

Specifically, as part of routine clinical care, all pregnant women will receive the following standard services from the MOH per national guidelines: All pregnant women will receive HTS using the opt-out method at their first ANC visit. All pregnant women diagnosed with HIV infection will undergo ART education and counselling and confirmatory HIV testing prior to starting first-line regimen 5A

(TDF/3TC/EFV) as soon as possible. Women with a documented contraindication to 5A (e.g. renal failure or psychosis) will start an alternative regimen in accordance with national guidelines. Maternal prophylaxis will include cotrimoxazole (CPT) and isoniazid preventive therapy during pregnancy and thereafter per national guidelines. HIV RNA will be measured at 6 and 24 months post ART initiation, and every 2 years thereafter or in the event of clinical suspicion of treatment failure based on new WHO Stage 3 or 4 condition after more than 12 months on ART. Mothers will be seen monthly for 6 months after the initial ANC visit and then quarterly per Malawi HIV program recommendations.

As part of routine care provided by the MOH, all HEIs will be enrolled in the national HIV Care Clinic and will receive 12 once-monthly follow-up visits as detailed on the national pink card. HEIs will also receive nevirapine prophylaxis, CPT, HIV testing services, feeding counselling, and immunization services per national guidelines.[36]

6.5.2.3.8 Community-based tracing and study activities for nested cohort participants

For women who do not have an upcoming scheduled visit or who have been classified as "lost to follow-up" per the MOH definition, we will use all available locator information in the routine care records to contact the client by phone and/or home visit to consent the mother and conduct the questionnaire and viral load testing at a mutually agreed upon time and setting (e.g. the health facility, the client's home, or other private location in the community). In this way, we will efficiently assess biological outcomes for *all* MIPs randomly selected at baseline.

Once the need for tracing is determined, potential participants will be sought first via telephone if a phone number is available from the treatment master card. Failing that, the potential participant will be sought in person using information available in the clinic files or master card, CFL provider files or known by HCWs or CFL providers, via study or public transportation, bicycle or on foot, as appropriate. Once found, tracers will encourage the patient to return to care. In-person tracing will be conducted with study staff trained in patient confidentiality, HIV psychosocial counselling and the protection of human research participants, leveraging existing CFL program and MOH-supported community-based tracing systems established in selected districts.

Once found, study staff and MOH CHWs and/or CFL providers will encourage the patient to return to HIV care in the national program, as is routinely done in MOH health facilities and by most CFL programs. While in the community, study tracers will obtain informed consent to proceed with study

procedures (including DBS sample collection for mother and infant and asking the mother to complete a structured questionnaire). Study tracers will follow a standard operating procedure (SOP) developed specifically for this study that details how to identify a mutually agreed upon safe and confidential location to carry out community-based study procedures. Data will be collected from patient next-of-kin if the patient is dead. Potential participants who choose not to participate in the study will be replaced by the next randomly selected woman from the clinic enumeration list as noted under section 6.5.2.3.2 above.

6.5.2.4 Maternal In-Depth interviews (IDIs)

6.5.2.4.1 Sampling for Maternal IDIs

From among the MIPs enrolled in our nested cohort at the approximately 10 sites selected for in-depth site assessments (see Section: 6.5.1.1), we will purposively select approximately 60 HIV-positive mothers, or about 6 clients per site, to conduct IDIs examining their experiences with CFL models of interest and the SOC. We believe this number will be sufficient to identify major and minor themes, and achieve saturation of factors relating to MIP engagement with and retention in the national PMTCT/ ART program.

6.5.2.4.2 Eligibility and Recruitment for Maternal IDIs

We will seek to include a variety of women ≥16 years of age from our nested cohort, including women from all 5 study districts and women accessing services at different tiers of the health system. We will recruit these women for IDIs from among consenting nested cohort participants using a quasi-random selection; that is, selection of approximately every fifth to n^{th} woman participating in the nested cohort during the study period until about 6 women at the site have been enrolled and interviewed. Where a potential interview candidate declines to participate, a replacement will be selected until we achieve our IDI enrolment target.

6.5.2.4.3 Consent Procedures for Maternal IDIs

Participation in the IDIs will be completely voluntary and subject to written informed consent in the participant’s choice of English, Chichewa, Yawo, or Tumbuka. IDI facilitators will explain to potential participants that in order to ensure optimally accurate response capture, individuals who do not wish to have their voice recorded will be ineligible to participate. Facilitator will also explain that participants do not need to answer any question that makes them feel uncomfortable. All those taking

part in IDIs will be eligible for the Malawi Kwacha equivalent of $4 in transportation reimbursement, and a snack and refreshment for their participation.

6.5.2.4.4 Data Collection for Maternal IDIs

Interviews will be framed by semi-structured IDI guides developed to cover issues demonstrated in the literature to be important to understanding retention in care for MIPs, including: psychosocial support, social networks, gender inequality, male partner involvement, stigma, financial barriers, and other structural issues. IDI guides will include a section asking participants to reflect on their experiences working with CFL providers and negotiating the PMTCT care continuum, including how information availability, socio-cultural norms, and the ways CFL providers and HCWs treat clients affect past and current health seeking behaviour. IDI questions will be open-ended to enable research assistants to probe for causal mechanisms influencing participant behaviour.

Interviews will be structured to elicit information about participants' perceptions, choices and behaviour in relation to their care-seeking history both for themselves and their children. IDI guides will be constructed with reference to existing literature about barriers and facilitators to retention in the PMTCT care continuum both for themselves and their children, but will involve iterative and open-ended discussions that enable new themes and/or experiences to be discussed. For participants who are alive and in care, we will seek to understand the barriers and facilitators of engagement and the perceived utility and experiences with CFL providers. For participants alive and no longer in care or in care elsewhere, or who have a HEI who died, we will start with the same set of questions, but expect that the issues and experiences brought up through probing will differ.

IDIs will last approximately 1 hour, and will be conducted in a private space. IDIs will be conducted in English, Chichewa, Yawo, or Tembuka based on participant preference. All IDIs will be audio-recorded (to ensure optimal response capture), and then transcribed verbatim and translated into English in a single step by trained study staff. Audio-recordings will not be marked with any identifying information.

6.5.2.4.5 Additional Procedures for Maternal IDIs

As a back-up form of data capture, extensive hand-written notes will be taken during interviews and typed up in full as soon as possible. Digital audio-recordings will be stored on a password-protected computer, accessible only to trained study staff. After transcription, audio recording will be stored for

a maximum of 2 years and then destroyed. Transcriptions and notes will be imported into software for managing qualitative analysis (e.g. NVivo, Atlas TI, Dedoose) then subject to an iterative process of coding using multi-step processes of deductive and inductive techniques. The UNC Project—Malawi team will share the data with the UNC Chapel Hill team and other authorized co-investigators through an online file sharing system that is consistent with all organizations' data protection policies.

6.6 Data Collection & Management

6.6.1 Data Collection Overview

We will leverage UNC's on-going health worker mentorship activities in Lilongwe district, as well as UNICEF OHTA's and other study partners' monitoring and evaluation and health system strengthening activities in the remaining study districts, to support complete and accurate data collection on routine data recording and reporting tools, including MOH registers and treatment cards. Routine data from these existing registers and treatment cards will be collected for our retrospective cohort. Study-specific data for site assessments, structured surveys, structured observations, FGDs, IDIs, and nested cohort outcome ascertainment will be collected separately by study staff using study-specific case reporting forms and other paper-based data collection tools.

6.6.2 Data Collection by Objective: Summary Outline

The following outline summarizes the organization and hierarchy of data to be collected under this protocol, and how each component contributes to the completion of our primary research objectives:

I. **Objective 1**
  a. Site Assessments (Rapid and In-Depth)
    i. Community-Facility Linkage (CFL) model survey
    ii. Structured Field Observations
      1. Public observations
      2. Private observations
    iii. Focus Group Discussions (FGDs)
      1. Professional/ Lay Health Care Workers
      2. CFL Providers
    iv. Managerial In-depth Interviews (IDIs)
      1. CFL Programme Site Supervisors & Regional / National Managers
      2. District Health Management Team Representatives

II. **Objective 2**

a. Chart Review (abstraction of existing programmatic data only)

b. Nested Cohort (primary collection of study-specific data)

  i. Biological outcome ascertainment

    1. Maternal

    2. Infant

  ii. Structured client questionnaire

  iii. Maternal in-depth interviews

6.6.3 Data Collection & Management Procedures

Routine MOH clinical and program records at study sites will be maintained in locked rooms per MOH standard operating procedures. These records contain identifying information, including name, date of birth, and addresses. Clinicians, Nurses, HSAs and Clerks are responsible for accurately filling the registers and treatment cards for clients during clinic visits. A trained and experienced UNC Data Associate will abstract existing clinical and demographic data from these routine data sources for the purposes of our retrospective cohort, and enter them into a secure Microsoft Access (Redmond, Washington, USA) database. Routine, existing data to be entered will encompass socio-demographic and clinical information, including documentation of HIV exposure, EID and HTC testing dates, and HIV rapid test, DNA PCR, and RNA PCR (i.e. maternal viral load) results, as well as baseline clinical data (e.g. WHO clinic stage), HCC and ART program enrolment dates, and ART initiation information (e.g. ART start date and ART regimen) for women found to be HIV-infected and their HIV-exposed/ HIV-infected infants.

All study-specific paper data on study forms, logs, registers, and other documents will be maintained in secure, locked filing cabinets in locked rooms at selected study sites and will only be accessible to study staff during working hours. In Lilongwe, these documents will be kept at the UNC Project Office situated on the grounds of Bwaila Hospital. In Neno, documents will be stored in a secure location at the Partners In Health Offices or the Neno District Health Office based on available space. In Mzimba North/South, Salima, and Zomba, study records will be stored securely in locked filing cabinets in a locked room identified by the DHO’s office or CFL program partner. Once field-based study activities are completed, all study-specific forms will be stored at the UNC Project Office in Lilongwe. In all cases, personal identifying information and health information will be stored separately and will only be linkable through the use of a link log stored in a separate, secure location.

All electronic data will be saved onto encrypted password-protected computers and/or tablets and uploaded to the encrypted password-protected UNC Project server at regular intervals. Quality assurance and quality control procedures will be in place throughout the project, with regular assessment of missing or inconsistent records. We will conduct regular database queries of extreme and missing values. The PI, co-PI and the data manager (contact information above) will be ultimately responsible for all data security issues. All data will be stored per NHSRC guidelines. All identifying data will be purged from the database prior to creating the analytical dataset.

7.0 Data analysis

7.1 Study Outcomes

We will use routinely available program data for our retrospective cohort enriched by prospective nested cohort data to ascertain the following outcomes:

Primary Outcome:

- 12-month post-ART initiation retention in care for HIV-positive mothers

Secondary Outcomes:

- Proportion of pregnant women newly registered in ANC who received HIV testing services
- Proportion of HIV-infected PMTCT clients initiated on anti-retroviral therapy
- 6-month post-ART initiation retention in care for HIV-positive mothers
- Proportion of HIV-positive mothers achieving HIV virological suppression from 6 months post-ART initiation (at MOH-recommended times of 6 and 24 months post-ART and, through the study, at time points between 12 and 24 months post-ART initiation)
- 12-month infant HIV-free survival
- Exposed infant “early” HIV infection status (i.e. HIV-1/2 DNA PCR result) from age 6 weeks
- 24-month child HIV-free survival
- 12- and 24-month infant HIV sero-status
- Median time from PMTCT enrolment to maternal ART initiation
- Median age of ART initiation for HIV-infected infants and young children
- Proportion of HIV-infected infants and young children achieving HIV virological suppression (from 6 months post-ART initiation per MOH guidelines)

- Proportion of HIV-infected mothers and infants who "returned to care" within 30 and 60 days of meeting the national ART program definition of loss to follow-up/ "defaulting" (i.e. not known to have transferred out, stopped or died AND have not returned to the clinic for 2 months after the woman is expected to have run out of ARVs).
- Proportion of HEIs who "returned to care" within 30 and 60 days of meeting the national HIV Care Clinic (HCC) programme definition of loss to follow-up/ "defaulting" (i.e. not known to have transferred out, stopped or died AND have not returned to the clinic for 2 months after the "next appointment date" given at the last HCC visit).

7.2 Analysis Plan by Objective

7.2.1 Analysis Plan for Objective 1

7.2.1.1 Survey Data

We will present proportions for CFL components that can be presented as categorical variables, such as: the proportion of facility catchment areas with observed, practicing CFL providers (i.e., expert clients, mentor mothers, or CHWs); proportion of catchment areas with more than one CFL model observed operating; proportion of CFL providers in selected facility catchment areas who are remunerated; proportion of facility catchment areas offering one or more women's support groups; proportion of facility catchment areas offering one-on-one client counselling; proportion of facility catchment areas providing follow-up after a missed visit (e.g., by phone call, SMS, or home visit); among others. For CFL components that are best summarized as continuous variables, we will calculate means with standard errors or medians with interquartile ranges, as appropriate, for such variables as: average number of CFL providers per facility catchment area; average number of clients per CFL provider; average CFL provider catchment area population size; average number of CFL encounters provided to clients per month; average number of "defaulter" tracing contacts with clients per month; average number of CFL provider contacts with clients during the first 3 months post-ART initiation; average mentor mother/ expert client/ CHW remuneration; etc. We will compare characteristics between CFL models using the Chi-square and Fisher's Exact test for categorical variables, and the Student's t-test and one-way ANOVA for normally distributed continuous variables. For other continuous variables, we will use the nonparametric Wilcoxon Rank-sum test and Wilcoxon Signed-rank test as appropriate.

7.2.1.2 FGD, IDI, and Structured Observation Data

To contextualize our survey findings, we will conduct a qualitative analysis involving FGD and IDI transcripts and research memos from structured observations. Specifically, we will search for patterns in the data that help: 1) explain the efficacy, fidelity, and efficiency of CFL models and the SOC; 2) describe the various capabilities of CFL providers and the systems in which they operate; and 3) define the most salient "core elements" of each CFL model and 4) establish distinguishing and common features for each CFL model.

To identify major and minor themes that explain those patterns, we will analyse FGD transcripts and structured observation memos using content analysis methodology and appropriate analytical software (i.e. NVivo, Atlas TI, Dedoose, etc.). The process of eliciting themes will involve: a) familiarization through careful reading of FGD transcripts and research memos, noting emergent themes; b) performing open coding in which codes are created based on identified themes, codes are assigned to specific sections of transcripts, and double-coding is conducted on a sample of data to promote inter-coder reliability; c) developing a codebook; d) performing data reduction in which an inventory is taken of what is related to the given code, capturing the variation or richness of each theme and noting differences between individuals or among subgroups; e) data display using matrices and tables; and, f) interpretation in which we search for relationships among themes or concepts identified and develop diagrams in order to map out relationships in the data. Particular attention will be paid to the rationale for how themes are grouped, and to the types of messages grouped together. Two investigators, one of which will include the principal investigator, will review all qualitative data, independently identify emergent themes, and confer to agree upon final coding and findings.

For all qualitative analyses, we will apply established qualitative research methods including principles of triangulation, negative case analysis, and respondent validation. Triangulation, a powerful tool to cross-validate qualitative research findings, refers to comparing results from multiple sources (in this case, results of structured observations and focus group discussions, in addition to the findings of in-depth interviews from Objective 2 below) to identify patterns of data convergence or divergence across the models being studied. Negative case analysis involves the identification of experiences that appear to contradict the theoretical assumptions underpinning the study (i.e. that CFL models promote MIP retention and engagement in the PMTCT continuum) and generates rival explanations. Respondent

validation and peer debriefing allow for the review and confirmation of preliminary descriptions and findings with research participants. The aforementioned methods will be used in combination to ensure that our qualitative findings are internally valid and that our conclusions are robust.

7.2.2 Analysis Plan for Objective 2 & 2a

7.2.2.1 Cohort Data

We will calculate maternal retention in care as the proportion of mothers alive and in care at 6, 12, and 24 months post-ART initiation among all eligible women at the start of the cohort. We will also calculate median days from PMTCT enrolment to ART start to examine the effects of CFL models on time to ART initiation. We will look for differences in the delay of ART initiation across the CFL models and SOC using the Aalen-Johansen estimator to account for the competing event of death. We will report crude HEI mortality at 12 and 24 months as part of our survival analysis. We will define infant 12-month HIV free survival as the proportion of HEIs alive with a negative 12-month HIV-rapid test among all infants enrolled in the HEI birth cohort. Similarly, we will define infant 24-month HIV free survival as the proportion of HEIs alive with a negative 24-month HIV-rapid test among all infants enrolled in the HEI birth cohort. We will use Kaplan Meier techniques to compare infant HIV-free survival at 12 and 24 months across the 3 CFL models and SOC accounting for informative censoring and differences in baseline patient characteristics between the models using inverse probability weights. We will censor all survival outcomes on the last date of field activities in a given district (most likely between February 1, 2018 and April 30, 2019, depending on the district), the date of transfer, or the date of the last clinical encounter, as appropriate.

We will explore associations between CFL model and individual-level maternal care retention and infant HIV-free survival at 12 and 24 months using multivariate log binomial regression modelling. We will estimate the effect of receiving any CFL service on the risk of 12-month maternal LTFU and viral suppression, controlling for other measured individual- and catchment area-level covariates, including age, pregnancy number, district, facility type, and number of women registered in the PMTCT cohort, among others; we will conduct a sub-group analysis for women who initiated ART while breastfeeding. We will repeat this analysis to examine effects of CFL model components—including client-to-provider ratio, frequency of provider-client interaction, pro-active versus reactive visit adherence approach, presence of CFL provider remuneration, CFL provider training frequency and duration, availability of one-on-one psychosocial support, and other variables—on maternal 12-month LTFU, maternal viral

suppression, and infant 12- and 24-month HIV-free survival. For maternal viral suppression, we will use survival analysis methods to control for variability in the timing of outcome ascertainment and to enable comparison of viral suppression between groups accounting for interval censoring.[38, 39] For the 24-month HIV-free survival outcome, we will control for infants who do not reach 24 months of age by the time of outcome ascertainment by excluding them from the analysis (so that the outcomes for all infants are represented by those who made it to 24 months in the study). As a check on the robustness of this approach, we will conduct a sensitivity analysis and use inverse probability weighting of those with outcomes at 24 months (compared to the total study sample) to account for any potential differences between infants who have and do not have a 24-month outcome at the time of field outcome ascertainment. Risk differences, unadjusted risk ratios, and adjusted risk ratios with accompanying 95% confidence intervals will be estimated for each model. Of note, these estimates will include the MIPs lost to routine care who are traced through the study, therefore allowing for more robust estimates of true outcomes in the study cohort.

All estimates will be corrected based on the outcomes of sampled MIPs using methods established for general HIV programs.[40] The weights for outcomes of interest will be derived from the product of the inverse probability of sampling for the facility catchment area (1/[0.25]), and the inverse probability of sampling for the MIP (between 1/[80/100] and 1/[10/100] depending on the final enumeration cohort), and the inverse probability of participation given that a MIP was sampled ($1/[P(R = 1 | W = w)]$).

7.2.2.2 90-90-90 Virtual Survey Analysis

Using data from the full retrospective cohort, enriched by nested cohort data, we will calculate HIV care cascade indicators for MIPs and compare results across CFL models/SOC according to established UNAIDS 90-90-90 definitions (i.e. 90% of women and infants know their HIV status; 90% of HIV-infected women and infants are sustained on ART; and 90% of women and infants on ART achieve viral suppression). The following 90-90-90 indicators will be calculated and presented together with their associated 95% confidence intervals (CIs):

Proportion of women who know their HIV status (i.e. "*Known Maternal HIV status*"): For this indicator examining the first step of the HIV care cascade (i.e. the "first 90"), we will define our denominator as the number of pregnant women with a recorded ANC or Maternity Department visit in the 12 months preceding the evaluation date (e.g. the last day of study activities in the targeted district). For the numerator, we will calculate the number of pregnant women with either a documented negative HIV test result in ANC or Maternity within the last 12 months (of the evaluation date) or documented positive HIV test/ evidence of being HIV-positive before the evaluation day (e.g. a women is known to be receiving ART).

Proportion of infants who know their HIV status (i.e. "*Known Infant HIV status*"): For this indicator, we will define our denominator as the number of HIV-exposed infants enrolled in the national HIV Care Clinic follow-up program or registered in the MOH EID testing logbook in the 12 months preceding the evaluation day (e.g. the last day of study activities in the targeted district). For the numerator, we will ascertain the number of infants with either a documented negative DNA PCR or rapid HIV test result within the last 12 months (of the evaluation date) or documented positive HIV test/ evidence of being HIV-positive before the day of evaluation (e.g. infant is known to be on ART).

Proportion of women receiving anti-retroviral therapy (i.e. "*Maternal ART Initiation*"): For this second step of the HIV care cascade (i.e. "the second 90"), we will calculate the proportion of HIV-infected pregnant and breastfeeding women initiating ART during the 12 months preceding the evaluation day among all PMTCT clients with a documented positive HIV test/ evidence of being HIV-positive before the day of evaluation.

Proportion of infants/ young children receiving anti-retroviral therapy (i.e. "*Infant ART Initiation*"): Similar to the related maternal indicator, for this infant ART initiation indicator, we will calculate the proportion of HIV-infected infants/ young children initiating ART during the 12 months preceding the evaluation day among all infants with a documented positive HIV test/ evidence of being HIV-positive before the day of evaluation.

Proportion of PMTCT mothers and infants/young children who started ART and have a suppressed HIV viral load ("*Viral suppression*"): Using viral load test result data, we will calculate and present separately the proportion of ART-initiated, HIV-infected mothers ***and*** ART-initiated HIV-infected infants/ young children who achieved viral suppression. For this final step of the cascade (i.e. the "third 90"), we will use the numerators from the maternal and infant ART initiation indicators for our respective denominators, and will assess the subset of these denominators with documented suppressed viral load test results within 12 months of the evaluation day. We will define viral suppression using a number of established thresholds for HIV-1 viral load, including less than 20 copies/ ml (the lower limit of detection for VL testing performed on plasma collected by venipuncture), less than 550 copies/ml (the lower limit of detection for VL testing performed on DBS) and less than 1,000 copies/ml (the definition of viral suppression used in the recent MOH Malawi Population-Based HIV Impact Assessment).

7.2.2.3 Maternal IDI Data

For qualitative data, the same analytical approach will be employed as described for Objective 1 above, except that we will search for patterns in the data that help explain client experiences with CFL model services and navigating the PMTCT care continuum for mothers and their children.

8.0 Results Dissemination & Expected Results

8.1 Results Dissemination Strategy

Dissemination of results will occur through three mechanisms: 1) a report to the NHSRC and HIV Unit of the Ministry of Health; 2) a presentation at the annual National AIDS Commission research dissemination meeting; and 3) a manuscript published in an international, peer-reviewed journal. All presentations, abstracts, and manuscripts derived from this impact evaluation will be made available for review by our partners, including NHSRC and the HIV Unit of the Malawi Ministry of Health.

The primary audience for results dissemination and utilization will be policy makers and program managers working on PMTCT programs in Malawi and elsewhere in sub-Saharan Africa. To ensure that we reach our target audience, stakeholder engagement will be a major, continuous focus of the project from the time of protocol development through study conclusion. In addition, to ensure that our study is conducted to the highest ethical and methodological standards, and that it addresses relevant evidence gaps, we will convene a Study Advisory Committee (SAC). The SAC's role during the study will be to provide guidance and strategic recommendations on stakeholder engagement and the direction, performance, and results of the study to ensure the best achievable outcome within the time frame and financial resources available. During study implementation, the SAC will meet periodically by teleconference or in-person, realistically no more than twice annually, and will be asked to provide input and suggestions on: the study protocol; study operations to meet the study objectives; dissemination and stakeholder/ community engagement; data analyses and possible sub-studies; and strategies to support inclusion of relevant lessons learned into district, national, and regional policy. We will appoint at least 3 experts to the SAC with knowledge of the subject matter and context, each of whom will provide guidance to the study team. Among these experts, we will include at least one UNC faculty expert, one high-level government official from the Malawi Ministry of Health, one member of the USAID/Malawi health team, one member of the district health management team from a selected district with experience in community-based service delivery (e.g. the District Health or Nursing Officer), representatives from NGO focused on providing CFL services (e.g. mothers2mothers, Tingathe, and Dignitas), as well as one or more other prominent local experts who have extensive prior research experience. SAC members will be invited to comment on circulated first drafts of the study protocol and survey, interview guides, and other study tools either virtually, or preferably, during a pre-launch SAC meeting or teleconference.

Interim and final study results will be shared with the HIV Unit/ MOH, USAID, and other stakeholders. We will report all interim results available through the end of calendar year 2017 and early 2018 to HIV Unit/ MOH, USAID/Washington and USAID/Malawi at that time. Interim results will also be presented via a study brief and PowerPoint in mid-2018 at a local dissemination meeting held at UNC Project headquarters. Final study results will be presented at a national-level dissemination meeting in mid-to-late 2019 that will include a PowerPoint presentation, information about data sharing and a discussion with stakeholders about the interpretation of results and action plans for their use. A full report or a summary document will also be distributed at the meeting.

8.2 Community Engagement

We will engage the community in catchment areas where we plan to conduct study activities through a variety of mechanisms, including through our CFL program partners who maintain strong ties to the community, as well as via pre-launch community sensitization activities. We will work with the UNC Project—Malawi Community Advisory Board to proactively address any concerns presented by the community throughout the life of the study, and to develop appropriate community messaging about study activities and findings. Finally, we will hold district-level dissemination meetings following national-level dissemination to ensure that important study findings are shared with the community participants and health workers who made the study possible.

8.3 Expected Results

We expect this study will provide evidence about the role of CFLs in improving MIP health outcomes and supporting HIV-positive mothers to navigate the PMTCT continuum of care. We expect this research to generate a clear typology of CFL models that will enable common terminology, and a shared focus on the most essential elements of CFL models, to be used among stakeholders when discussing strategies for retaining MIPs in PMTCT programs in the region. Lastly, we envision that our study will identify those components of community-based client support that are associated with maternal retention in care and infant HIV-free survival, helping to refine and accelerate scale up of promising CFL models in Malawi and elsewhere in sub-Saharan Africa.

9.0 Ethical considerations

9.1 Overview

As we propose to use largely existing routine care data, the risks incurred with the study activities described herein are minimal. The principal risk involved is the possibility of an inadvertent disclosure of confidential patient health information, which could include HIV status. At all steps of the study, we will protect participant privacy and confidentiality to reduce this risk.

9.2 Physical Risks

Blood sample collection carries a small risk of pain, bleeding, bruising, and (in rare cases) infection. To minimize these risks, the following measures will be taken: 1) only properly trained study staff will perform or support MOH-led blood sample collection activities; 2) the PI will review with study staff how to properly carry out phlebotomy procedures; 3) sterile technique will be used for all blood draws; and 4) we will seek to obtain specimens at only one time point. Based on these measures, the magnitude and likelihood of phlebotomy risk is expected to be consistent with those normally encountered during the course of routine medical care for healthy persons.

9.3 Psychosocial Risks

Study participants may become embarrassed, worried, anxious, or uncomfortable when discussing sensitive topics during FGDs or IDIs, when answering questions from the nested cohort questionnaire, or when approached during tracing activities in the community. Study staff will be trained how to identify and mitigate issues that may cause psychosocial distress, and will use established counselling techniques when discussing participant challenges with HIV care. There is also a risk of inadvertent disclosure of HIV status or other personal health information.

9.4 Methods to Minimise Risk

All participants will be instructed during the informed consent process that they may discontinue any study procedure, including questionnaires and IDIs at any time for any reason, such as experiencing psychosocial stress related to any question(s). Risks relating to psychological discomfort will be further minimized by: 1) reducing respondent burden through streamlining of questionnaires and IDI guides; and 2) referring participants to appropriate counselling, as necessary. We do not anticipate that focus group discussions will pose any risk of psychological discomfort to stakeholders beyond that encountered in the course of everyday professional conversations. However, due to the potentially

sensitive nature of questions surrounding perceptions of the functionality of the health system or CFL models, we will ensure through a comprehensive verbal informed consent process that participants are aware of: 1) their right to withdraw; 2) their right to not respond to any question(s) they do not wish to answer; and 3) that their responses will not be linked back to them in any way or shared with their employers or supervisors.

We will strive to minimize the risk of unintended confidentiality breeches involving study-related information by: 1) integrating our study procedures within routine health-related operations whenever possible so as not to draw attention to study participants; 2) conducting nested cohort enrolment procedures, IDIs, and FGDs in private settings that are conveniently located for participants such that all information is kept strictly confidential and not inadvertently disclosed to anyone not involved in the study activity; and 3) engaging trained CFL providers to continue providing routine stigma mitigation messaging and report any instances of harassment to the study team.

We will use our team's previous experience with patient tracing under the OHTA project[41] to ensure patient confidentiality is maintained at all times. Tracers will be well-trained and will only state generic health-related purposes or other socially acceptable, non-identifiable reasons for any home visits. We will also work closely with HSAs and CFL providers whenever possible, and will ensure that there is a female research assistant or other female colleague present for all tracing activities to ensure maximal gender sensitivity during study activities. Study staff involved in tracing will not wear any identifiable clothing or badge when making home visits. All study staff members will be trained in appropriate implementation methods.

All information collected on paper during the course of this study will be kept securely and confidentially in a locked cabinet. Case report forms will be identified using the participant's study number only. All identifying study information, including participant locator information used for tracing activities, will only be available to study staff and will be stored securely, separately from the other information we collect from participants. Any other information we collect will be identified on forms and computer or tablet files by a study number only, not the participant's name or other identifying information. Information entered into the study database will be kept separately from participant identifying details and protected by a password. Only restricted study staff will have access to and be able to link identifying details with study data, ensuring that the participants' information

remains confidential. Indeed, data security measures will be strictly adhered to, including the immediate de-identification of data for analysis and the storage of data on secure, password-protected servers and encrypted computers and tablets.

Participant information will remain confidential at all times, unless we are required by law to release information. Reports about the study and results that may be published in scientific journals will not include any identifying information that would allow participants identities to be revealed or deduced.

9.4 Anticipated Benefits to Participants

There may be no benefit to individuals who participate in this study. However, participants who have defaulted from the national program may benefit from being followed up by trained study staff and may decide to re-engage in care following phone contacts or home visits. Participants receiving study-supported maternal viral load or infant DNA PCR or rapid HIV testing may receive the benefits of early detection of treatment failure or infant HIV infection. Finally, if the study reveals ways to improve CFL services or MIP retention along the PMTCT care continuum, then future patients, and in some cases the individuals providing data themselves, may benefit.

9.5 Participant Compensation

As nested cohort study activities are intended to reinforce and in some cases enhance the existing standard of care, and will take place once, aligned with the schedule of routine care, compensation will follow local recommendations of providing the Malawi Kwacha equivalent of $4. Because in-depth interviews may take 1 hour or more to complete, these individuals will be reimbursed the Malawi Kwacha equivalent of $4 for their time and travel expenses. Lastly, since FGDs will last 1 to 2 hours, and in many cases will require travel to a central location in the district, travel reimbursement in the Malawi Kwacha equivalent of $4 will be provided to FGD participants.

9.6 Safety Monitoring and Unanticipated Problems

Unanticipated problems involving risks to study participants refers to any incident, experience, or outcome that:

- Is unexpected (in terms of nature, severity, or frequency) given the research procedures that are described in the protocol-related documents, such as the IRB-approved research protocol and consent documents;

- Is related or possibly related to an individual's participation in the research; and
- Suggests that the research places participants or others at a greater risk of harm (including physical, psychological, economic, or social harm) related to the research than was previously known or recognized.

Given the observational nature of this implementation research study and the minimal risks involved with participating, we do not foresee any unanticipated problems taking place. Because we will be enhancing the standard of care for nested cohort participants by tracing patients who have disengaged from HIV care, and by offering viral load and EID testing for mother-infant pairs, we feel the risks associated with participation can be categorized as minimal. Nevertheless, the study PI and co-investigators will be vigilant in reviewing study data to ensure participant safety. We will put into place a data and safety monitoring plan that calls for regular review of study data by the PI and the study manager, Mr. Joseph Njala, who is an accomplished Malawian clinician with years of experience caring for MIPs in the Malawi HIV and PMTCT programs and helping develop national guidelines. The PI and Mr. Njala will review compiled data on nested cohort participants at regular intervals, particularly quantitative maternal and infant HIV-1 viral load and infant EID test results, to look for evidence of virological failure among ART-treated mothers and infants, and new HIV diagnoses among HEIs, which are expected to be the most common adverse events. Any mother or infant found to have a viral load over 1,000 copies/ml will be referred to the nearest MOH health facility for intensive adherence support counseling and clinical evaluation for repeat viral load testing and potential switch to second-line ART in accordance with national HIV guidelines. Similarly, any HEI identified with new HIV infection will be referred to the nearest MOH health facility for clinical evaluation and immediate ART initiation per national guidelines. All cases of suspected social harm will be documented on a case reporting form, reviewed by the study PI, mitigated directly whenever possible, and reported to all local and international regulatory bodies. All study staff will be trained to follow a study standard operating procedure detailing how to activate existing, routine referral mechanisms in all cases where a participant is suspected of being in need of medical attention or psychosocial support. In all instances, we will look to leverage our existing robust collaborations with MOH health facilities, CFL programmes, and other non-governmental implementing partners built over 20 years of UNC Project's presence in Malawi to strengthen HIV diagnostic and monitoring services, promote participant engagement in HIV care, and enhance the existing standard of care.

9.7 Study Discontinuation

This study may be discontinued at any time by the sponsors, the Ministry of Health, or UNC as part of their duties to ensure that research participants are protected. Individual-level study data will only be used for analyses related to this study; summary statistics and de-identified aggregate data may be used to improve routine service delivery in study districts. UNC Project—Malawi, UNC, and protocol-affiliated investigators and staff members will not use study data for other, unrelated analyses without the express permission of the MOH.

9.8 Regulatory Review

This protocol will be reviewed by the National Health Sciences Research Committee of Malawi (NHSRC) and the Biomedical Institutional Review Board of the University of North Carolina at Chapel Hill, USA (UNC IRB). Co-investigators from other institutions will establish reliance agreements, as applicable, to have their institutions follow guidance provided by NHSRC and UNC IRB. All study procedures will conform to U.S. and Malawian ethical standards regarding research involving human participants and will follow international guidelines for Good Clinical Practice.

10.0 Capacity Strengthening

Continuing UNC's track record for building local research capacity in Malawi, we envision this study as a platform to engage junior Malawian investigators more deeply in operational research and implementation science. We will accomplish this by leveraging UNC Project—Malawi's existing Fogarty Global Health Training Program in HIV implementation science to identify promising Malawian trainees who can engage meaningfully in the research described herein by way of a relevant sub-study or secondary analysis using data generated from this proposal. This study will also support Dr. Chagomerana (the senior analyst on this study) and other junior Malawian investigators in their career development, providing valuable preliminary data and small grant opportunities to develop their research skills and to pursue independent research funding.

11.0 Work Plan

The overall project duration is 26 months, including: standard operating procedures (SOP) preparation and refinement, training of study staff and briefing of district-level partners, field-based data collection, data cleaning and analysis, dissemination, and report and manuscript writing.

Study Activity	2017												2018												2019					
	1	2	3	4	5	6	7	8	9	10	11	12	1	2	3	4	5	6	7	8	9	10	11	12	1	2	3	4	5	6
Stakeholder engagement	■	■	■	■	■	■	■	■	■	■	■	■	■	■	■	■	■	■	■	■	■	■	■	■	■	■	■	■	■	■
Protocol development				■	■	■	■	■																						
Questionnaire, IDI/ FGD guide & CFL survey development					■	■	■	■																						
Regulatory/IRB review					■	■	■	■	■	■																				
CRF and SOP development & refinement								■	■	■																				
All IRB/ regulatory approvals received										■																				
Recruit and hire study staff							■			■																				
Develop database & data systems									■	■																				
Partner Mapping/ Facility catchment area sampling & selection									■	■																				
Training of study staff										■																				
Study implementation											■	■	■	■	■	■	■	■	■	■	■	■	■	■	■	■	■	■		
Data management & analysis											■	■	■	■	■	■	■	■	■	■	■	■	■	■	■	■	■	■		
Dissemination of interim results																		■												
PowerPoint presentation & summary document																													■	
Dissemination meeting																														■
Final technical report & manuscript writing																													■	■

12.0 Budget

12.1 Budget Justification

Overview

For this study, we will leverage existing routine data collection systems available from study partners, including UNICEF, Partners In Health, mothers2mothers, and others. As such, most of the infrastructure required to implement this study is already in place. We anticipate completing all study field activities in about 18 months, with 3 months of preparatory work in year 1 and 3 months of close-out activities in year 2 (apart from investigator-level analyses and report writing).

Malawi Personnel

Field Activity Supervisor: One supervisor will oversee all operational aspects of the study at 100% effort over 2 years.

Research Assistants: There will be 2 research assistants/ data collectors working at 100% effort for 2 years.

Senior Data Analyst: The Senior Data Analyst will donate 25% effort over 2 years to this project (data generated from this project will provide preliminary data for the Senior Data Analyst to develop independent funding proposals).

Data Officer: We will conduct data entry and management at 50% effort for 2 years.

Office-related Costs

Airtime: In order for staff to remain in contact, we have budgeted approximately $.5/person/day x 1,200 person-days.

Stationary: We have budgeted for $100/ month for 18 months to spend on supplies to include pens, stationary, printer cartridges, white board markers, flip chart, markets, etc.

Monthly vehicle rental: A UNC Project vehicle will be donated in kind for study activities.

Vehicle fuel, repairs, and maintenance: We have budgeted for $150/ month for 18 months to spend on vehicle fuel, repairs, maintenance, insurance, and any incidental parts.

Results Dissemination

Dissemination meetings: In years 1 and 2, we will hold a 1-day dissemination meeting with partners from GOM and civil society to present and discuss the results of our study at the cost of $2,000 per meeting.

13.0 References

1. Tenthani L, Haas AD, Tweya H, et al. Retention in care under universal antiretroviral therapy for HIV-infected pregnant and breastfeeding women ('Option B+') in Malawi. *AIDS (London, England)* 2014; **28**(4): 589-98.

2. MOH. Integrated HIV Program Report January - March 2016: Government of Malawi, Ministry of Health, 2016.

3. Barker PM, Mphatswe W, Rollins N. Antiretroviral drugs in the cupboard are not enough: the impact of health systems' performance on mother-to-child transmission of HIV. *Journal of acquired immune deficiency syndromes (1999)* 2011; **56**(2): e45-8.

4. GOM. Malawi AIDS Response Progress Report 2015. Lilongwe, Malawi: Government of Malawi, 2015.

5. UNICEF. Community-facility linkages to support the scale up of lifelong treatment for pregnant and breastfeeding women living with HIV. New York, New York: USA, 2015.

6. m2m. Current Practices to Improve Uptake, Retention, and Adherence to Option B+ in Malawi. Lilongwe, Malawi: mothers2mothers Malawi, 2014.

7. Wouters E, Van Damme W, van Rensburg D, Masquillier C, Meulemans H. Impact of community-based support services on antiretroviral treatment programme delivery and outcomes in resource-limited countries: a synthetic review. *BMC health services research* 2012; **12**: 194.

8. Kim MH, Ahmed S, Buck WC, et al. The Tingathe programme: a pilot intervention using community health workers to create a continuum of care in the prevention of mother to child transmission of HIV (PMTCT) cascade of services in Malawi. *Journal of the International AIDS Society* 2012; **15 Suppl 2**: 17389.

9. UNAIDS. On the Fast Track to an AIDS-Free Generation. Geneva, Switzerland: Joint United Nations Programme on HIV/AIDS, 2016.

10. Schouten EJ, Jahn A, Midiani D, et al. Prevention of mother-to-child transmission of HIV and the health-related Millennium Development Goals: time for a public health approach. *The Lancet* 2011; **378**(9787): 282-4.

11. Chimbwandira FM, E.; Makombe, S.; Midiani, D.; Mwansambo, C.; Njala, J.; Chirwa, Z.; Jahn, A.; Schouten, E.; Phelps, R.; Giselman, A.; Holmes, C.B.; Maida, A.; Gupta, S.; Tippett Barr, B.A.; Modi, S.; Dale, H.; Aberle-Grasse, J.; Davis, M.; Bell, D.; Houston, J. Impact of an innovative approach to prevent

mother-to-child transmission of HIV--Malawi, July 2011-September 2012. *MMWR Morbidity and mortality weekly report* 2013; **62**(8): 148-51.

12. Ware NC, Idoko J, Kaaya S, et al. Explaining adherence success in sub-Saharan Africa: an ethnographic study. *PLoS medicine* 2009; **6**(1): e11.

13. Merten S, Kenter E, McKenzie O, Musheke M, Ntalasha H, Martin-Hilber A. Patient-reported barriers and drivers of adherence to antiretrovirals in sub-Saharan Africa: a meta-ethnography. *Tropical medicine & international health : TM & IH* 2010; **15 Suppl 1**: 16-33.

14. Falnes EF, Moland KM, Tylleskar T, de Paoli MM, Msuya SE, Engebretsen IM. "It is her responsibility": partner involvement in prevention of mother to child transmission of HIV programmes, northern Tanzania. *Journal of the International AIDS Society* 2011; **14**: 21.

15. Cataldo F, Chiwaula L, Nkhata M, et al. Exploring the Experiences of Women and Health Care Workers in the Context of PMTCT Option B Plus in Malawi. *Journal of acquired immune deficiency syndromes (1999)* 2017; **74**(5): 517-22.

16. O'Gorman DA, Nyirenda LJ, Theobald SJ. Prevention of mother-to-child transmission of HIV infection: views and perceptions about swallowing nevirapine in rural Lilongwe, Malawi. *BMC public health* 2010; **10**: 354.

17. Posse M, Meheus F, van Asten H, van der Ven A, Baltussen R. Barriers to access to antiretroviral treatment in developing countries: a review. *Tropical medicine & international health : TM & IH* 2008; **13**(7): 904-13.

18. Busza J, Walker D, Hairston A, et al. Community-based approaches for prevention of mother to child transmission in resource-poor settings: a social ecological review. *Journal of the International AIDS Society* 2012; **15 Suppl 2**: 17373.

19. Marcos Y, Phelps BR, Bachman G. Community strategies that improve care and retention along the prevention of mother-to-child transmission of HIV cascade: a review. *Journal of the International AIDS Society* 2012; **15 Suppl 2**: 17394.

20. Bhutta ZAL, Z.S.; Pariyo, G.; Huicho, L. Global Experience of Community Health Workers for Delivery of Health Related Millennium Development Goals: A Systematic Review, Country Case Studies, and Recommendations for Integration into National Health Systems. Geneva, Switzerland: World Health Organization, 2010.

21. Torpey KE, Kabaso ME, Mutale LN, et al. Adherence support workers: a way to address human resource constraints in antiretroviral treatment programs in the public health setting in Zambia. *PloS one* 2008; **3**(5): e2204.

22. Rich ML, Miller AC, Niyigena P, et al. Excellent clinical outcomes and high retention in care among adults in a community-based HIV treatment program in rural Rwanda. *Journal of acquired immune deficiency syndromes (1999)* 2012; **59**(3): e35-42.

23. Herce ME, Elmore SN, Kalanga N, et al. Assessing and responding to palliative care needs in rural sub-Saharan Africa: results from a model intervention and situation analysis in Malawi. *PloS one* 2014; **9**(10): e110457.

24. Herce ME KN, Wroe EB, Keck JW, Chingoli F, Tengatenga L, Gopal S, Phiri A, Mailosi B, Bazile J, Beste JA, Elmore SN, Crocker JT and Rigodon J. . Excellent clinical outcomes and retention in care for adults with HIV-associated Kaposi sarcoma treated with systemic chemotherapy and integrated antiretroviral therapy in rural Malawi. . *Journal of the International AIDS Society* 2015; **[in revision]**.

25. Admon AJ, Bazile J, Makungwa H, et al. Assessing and improving data quality from community health workers: a successful intervention in Neno, Malawi. *Public Health Action* 2013; **3**(1): 56-9.

26. Shroufi A, Mafara E, Saint-Sauveur JF, Taziwa F, Vinoles MC. Mother to Mother (M2M) peer support for women in Prevention of Mother to Child Transmission (PMTCT) programmes: a qualitative study. *PloS one* 2013; **8**(6): e64717.

27. Zikusooka CMK-M, D.; Bwanika, J. B.; Akena, D.; Kwesiga, B.; Abewe, C.; Watsemba, A.; Nakitende, A. External Evaluation and Cost-Benefit Analysis of mothers2mothers' Mentor Mother Programme in Uganda: Executive Sumary, 2015.

28. Herce ME, Mtande T, Chimbwandira F, et al. Supporting Option B+ scale up and strengthening the prevention of mother-to-child transmission cascade in central Malawi: results from a serial cross-sectional study. *BMC infectious diseases* 2015; **15**: 328.

29. McNairy ML, Teasdale CA, El-Sadr WM, Mave V, Abrams EJ. Mother and child both matter: reconceptualizing the prevention of mother-to-child transmission care continuum. *Current opinion in HIV and AIDS* 2015; **10**(6): 403-10.

30. Zash R, Souda S, Leidner J, et al. HIV-exposed children account for more than half of 24-month mortality in Botswana. *BMC pediatrics* 2016; **16**: 103.

31. Estill J, Tweya H, Egger M, et al. Tracing of patients lost to follow-up and HIV transmission: mathematical modeling study based on 2 large ART programs in Malawi. *Journal of acquired immune deficiency syndromes (1999)* 2014; **65**(5): e179-86.

32. WHO. Service Availability and Readiness Assessment (SARA): Reference Manual. Geneva, Switzerland, 2013.

33. Beach MC, Keruly J, Moore RD. Is the quality of the patient-provider relationship associated with better adherence and health outcomes for patients with HIV? *Journal of general internal medicine* 2006; **21**(6): 661-5.

34. Ware NC, Wyatt MA, Geng EH, et al. Toward an understanding of disengagement from HIV treatment and care in sub-Saharan Africa: a qualitative study. *PLoS medicine* 2013; **10**(1): e1001369; discussion e.

35. Jewkes R, Abrahams N, Mvo Z. Why do nurses abuse patients? Reflections from South African obstetric services. *Social science & medicine* 1998; **47**(11): 1781-95.

36. MOH. Malawi Guidelines for the Clinical Management of HIV in Children and Adults, 4th Edition. Lilongwe, Malawi: Ministry of Health, 2016.

37. MOH. HIV Testing and Counseling Guidelines, 4th Edition. Lilongwe, Malawi, 2016.

38. Griffin JT, Fraser C, Gras L, de Wolf F, Ghani AC. The effect on treatment comparisons of different measurement frequencies in human immunodeficiency virus observational databases. *American journal of epidemiology* 2006; **163**(7): 676-83.

39. Hernan MA, McAdams M, McGrath N, Lanoy E, Costagliola D. Observation plans in longitudinal studies with time-varying treatments. *Statistical methods in medical research* 2009; **18**(1): 27-52.

40. Geng EH, Emenyonu N, Bwana MB, Glidden DV, Martin JN. Sampling-based approach to determining outcomes of patients lost to follow-up in antiretroviral therapy scale-up programs in Africa. *Jama* 2008; **300**(5): 506-7.

41. Kamanga E GB, Mofolo I, Mwale G, Mwale M, Chikonda J, Sherman J, Chinkonde J, Herce M. Returning HIV-exposed infants to care: Results from a pilot integrating infant defaulter tracing into the national Option B+ programme in Lilongwe, Malawi. 21st International AIDS Conference. Durban, South Africa; 2016.
