# Supplementary material for: Community-facility linkage models and maternal and infant health outcomes in Malawi’s PMTCT/ART program: A cohort study
Source: PLoS Med. 2021 Sep 17;18(9):e1003780. doi: 10.1371/journal.pmed.1003780 (PMC8516224; doi:10.1371/journal.pmed.1003780)
Supplement: S1 Appendix — (PDF) [file pmed.1003780.s003.pdf]

Study Site: □□□-□□

Date Completed □□/□□□/□□□□

CFL001: COMMUNITY-FACILITY LINKAGES SITE ASSESSMENT SURVEY

Part I: Facility Factors

Instructions: This part is to be completed through direct observation and through interviews with up to 7 key informants (e.g. Clinic/Facility In-Charge or Designee, ANC In-Charge or Designee, PMTCT Coordinator or Designee, ART In-Charge or Designee, Under 5 In-Charge or Designee, Pharmacist In-Charge, and Laboratory In-Charge).

SECTION 1: BASIC INFORMATION (6 Questions)		
FIND THE CLINIC/ FACILITY IN-CHARGE INTRODUCE YOURSELF, EXPLAIN THE PURPOSE OF THE SURVEY AND ASK THE QUESTIONS IN SECTIONS 1 – 4.		
1. Date of facility visit:	*dd/MMM/yyyy* □□/□□□/□□□□	
2. Day of the week of facility visit:	□□□	
3. Interviewee / Key Informant Cadre:	*Name (printed):* ______	
4. Study Site & Health System Level	*Refer to Study Site Code List*	a. □□□-□□
	1=MOH Health Centre<br>*2=MOH Rural/ Community Hospital*<br>*3=MOH District Hospital*<br>*4=MOH National Referral Hospital*<br>*5=CHAM Facility*<br>*6=Other (Specify)*	b. □<br>*(specify if other):* ______
5. District Information:<br>*Please pre-population this information with district-level DHS or HMIS information.*	*a. District Name (please print):* ______	
	b. District Total Population:<br>*c. District Total Adult Population:* ______	
6. Catchment Area Information:<br>*If key informant is unfamiliar with these figures, please check with HMIS or district M&E officer.*	*a. Approximate Adult Population in Catchment Area:* ______	
	b. Approximate Under 5 Population in Catchment Area: ______	
	c. Approximate Adult HIV Prevalence: ______	
	d. Proportion of Women in the Catchment Area who Have Completed Primary Education: ______	
	e. Under 5 Mortality in Catchment Area:	

Completed By: □□ Verified By: □ Entered By: □□ Date Entered: □□/□□□/□□□□

	f. Infant Mortality in Catchment Area:

SECTION 2: FACILITY INFORMATION (4 Questions)		
**Question**	**Options**	**Response**
7. Number of days ANC open per week:	*# of days, from 1-7*	□
8. Number of days ART open per week:	*# of days, from 1-7*	□
9. Which organization manages this clinic?	*1=Public (government)*<br>*2=NGO/ Not-for-profit*<br>*3=Mission/ Faith-based*<br>*4=Private-for-profit*<br>*5=Other (specify)*	□<br>*(specify if other):* ______
10. Total population in the catchment area:	*Specify estimated # of people according to official (government) figures (consider reviewing HMIS reports/ data)*	______

PART I, Module 1: SERVICE AVAILABILITY

SECTION 3: STAFFING (13 Questions)		
***[Please Read:** “I have a few questions on the staffing for this facility. Please tell me how many staff with each of the following qualifications are currently assigned to, or employed by, or seconded to this facility. Please count every staff member only once, on the basis of the highest technical or professional qualification. For doctors, I would also like to know, of the total number, how many are part-time at this facility.”]*		
**Qualification**	**Reported**	**Observed**
11. Doctors:	a. □□□	b. □□□
12. Clinical Officers:	a. □□□	b. □□□
13. Medical Assistants:	a. □□□	b. □□□
14. Nursing Professionals (including Midwifes):	a. □□□	b. □□□
15. Laboratory Technicians/ Assistants:	a. □□□	b. □□□
16. HIV Testing Counselors:	a. □□□	b. □□□

17. Diagnostic Assistants:	a. ☐☐☐	b. ☐☐☐
18. Health Surveillance Assistants:	a. ☐☐☐	b. ☐☐☐
19. Mentor Mothers (all programs); *Specify program(s)*:________________________	a. ☐☐☐	b. ☐☐☐
20. Expert Clients/ Patients (all programs); *Specify program(s)*:__________________	a. ☐☐☐	b. ☐☐☐
21. Community health workers (all programs); *Specify program(s):* ______________________________	a. ☐☐☐	b. ☐☐☐
22. Other types of Lay/ Peer Health Workers (all programs); *Specify program(s):* ______________________________	a. ☐☐☐	b. ☐☐☐
23. ***Total*** Lay/ Peer Health Workers (all types, all programs)	a. ☐☐☐	b. ☐☐☐

**SECTION 4: INFRASTRUCTURE (7 Questions)**			
***[Please Read:** "This next section will focus on questions related to infrastructure."]*			
**Question**	**Options**	**Reported**	**Observed**
**COMMUNICATIONS**			
24. Is there access to internet within the facility today?	*1=Yes*<br>*0=No*	a. ☐	b. ☐
**POWER SUPPLY**			
25. Does your facility have electricity from any source (i.e. electricity grid, generator, solar or other) including for stand-alone devices (i.e. EPI/vaccine cold chain)?	*1=Yes*<br>*0=No*	a. ☐	b. ☐
**BASIC CLIENT AMENITIES**			
26. On average, how many hours per day is this facility open?	*1=4 hours or less*<br>*2=5 to 8 hours*<br>*3=9 to 16 hours*<br>*4=17 to 23 hours*<br>*5=24 hours*	a. ☐	b. ☐
27. How many outpatient medical examination/treatment rooms are there in the facility?	*Specify # of rooms*	__________	__________
**PHARMACY/ ARV STORAGE**			

28. Based on your average volume of clients, could you estimate your storage capacity for HIV test kits?	*Enough space for…*<br>*1=1 week or less of testing*<br>*2=More than 1 week but less than 1 month*<br>*3=Between 1 and 3 months*<br>*4=Between 4 and 6 months*<br>*5=More than 6 months*	a. □	b. □
29. Based on your average volume of clients, could you estimate your storage capacity for ARVs?	*Enough space for…*<br>*1=1 week or less*<br>*2=More than 1 week but less than 1 month*<br>*3=Between 1 and 3 months*<br>*4=Between 4 and 6 months*<br>*5=More than 6 months*	a. □	b. □
**SUPERVISION**			
30. When was the last time this facility received a quarterly supervision visit from the MOH HIV Unit?	*1=This month*<br>*2=In the last 3 months*<br>*3=More than 3 months ago*<br>*9=Don't know*	□	

PART I, Module 2: AVAILABLE SERVICES

**SECTION 5: ANTENATAL CARE SERVICES (3 Questions)**		
ASK TO BE SHOWN THE LOCATION IN THE FACILITY WHERE ANTENATAL CARE SERVICES ARE PROVIDED. FIND THE PERSON MOST KNOWLEDGEABLE ABOUT ANTENATAL CARE SERVICES IN THE FACILITY, USUALLY THE "ANTENATAL IN-CHARGE". INTRODUCE YOURSELF, EXPLAIN THE PURPOSE OF THE SURVEY AND ASK THE FOLLOWING QUESTIONS.		
**Question**	**Options**	**Response**
31. Do nurses or other health workers provide the following services to pregnant women as part of routine ANC?	*1=Yes,*<br>*0=No*	a. HIV testing and counseling □<br>b. Folic Acid Supplementation □<br>c. Intermittent preventive treatment in pregnancy (IPTp) for malaria □<br>d. Tetanus toxoid immunization □

		e. Monitoring for hypertensive disorders of pregnancy □
32. Please tell me if the following documents are available in the facility today (**if available, ask to see the document**)?	*1=Yes, observed*<br>*2=Yes, reported, not seen*<br>*0=No*	a. National ANC Guidelines □<br>b. Any ANC checklists or job aids (if so, specify below) □<br>__________<br>__________
33. How many provider(s) of ANC services at this facility received any training on ANC in the last two years?		□□□

**SECTION 6: PREVENTION OF MOTHER-TO-CHILD TRANSMISSION OF HIV (6 Questions)**		
ASK TO BE SHOWN THE LOCATION IN THE FACILITY WHERE PMTCT SERVICES ARE PROVIDED. FIND THE PERSON MOST KNOWLEDGEABLE ABOUT PMTCT SERVICES IN THE FACILITY, USUALLY THE "PMTCT COORDINATOR" OR "ART IN-CHARGE". INTRODUCE YOURSELF, EXPLAIN THE PURPOSE OF THE SURVEY AND ASK THE FOLLOWING QUESTIONS.		
**Question**	**Options**	**Key Informant**
34. Does this facility provide the following services as a part of PMTCT/EID?	*1=Yes*<br>*0=No*	a. HIV testing and counseling (HTC) □<br>b. Couples' HTC □<br>c. Early Infant HIV Diagnosis (EID) Services for Infants □<br>d. ART/ Option B+ to HIV-positive pregnant women □<br>e. ARV prophylaxis to exposed newborns □

		f. Breastfeeding/ feeding counseling □<br>g. Infant/young child nutritional counseling □<br>h. Family planning □<br>i. Infant cotrimoxazole preventive therapy (CPT) □
35. Does the facility provide any of the above PMTCT services as outreach/ through health posts?	*1=Yes*<br>*0=No*	□<br>*(specify which ones if yes):* ________
36. Do you have national guidelines for PMTCT available in this facility today?	*1=Yes, observed*<br>*2=Yes, reported, not seen*<br>*0=No*	□
**Question**	**Options**	**Response**
37. How many providers of ART/PMTCT services received any training in ART/PMTCT in the last two years?		□□□
38. How many providers received any training in Early Infant Diagnosis (EID) in the last two years?		□□□
39. Is there a room available for PMTCT service delivery with auditory and visual privacy?	*1= Privacy from people hearing you only*<br>*2= Privacy from people seeing you only*<br>*3=Privacy from people seeing or hearing you*<br>*0=No privacy*	a. Reported □ b. Observed □

SECTION 7: Child Health Services (4 Questions)		
**Question**	**Options**	**Response**

40. Does this facility offer immunization services?	*1=Yes*<br>*0=No*	
41. Does this facility offer child preventative and curative care services?	*1=Yes*<br>*0=No*	
ASK TO BE SHOWN THE LOCATION IN THE FACILITY WHERE CHILD PREVENTATIVE AND CURATIVE CARE SERVICES ARE PROVIDED. FIND THE PERSON MOST KNOWLEDGEABLE ABOUT CHILD PREVENTATIVE AND CURATIVE CARE SERVICES IN THE FACILITY. INTRODUCE YOURSELF, EXPLAIN THE PURPOSE OF THE SURVEY AND ASK THE FOLLOWING QUESTIONS.		
42. Please tell me if this facility provides the following services?	*1=Yes*<br>*0=No*	a. Child Malnutrition Screening & Treatment b. Provide Vitamin A supplementation c.Provide ORS for children with diarrhoea d. Provide Zinc to children with diarrhoea e. Child growth monitoring f. Treatment/ give antibiotics for pneumonia g. Treatment of malaria in children
43. How many of the current clinical staff received training in the Integrated Management of Childhood Illness (IMCI) or the equivalent at any time in the last 2 years?		

**SECTION 8: HIV COUNSELLING & TESTING (7 Questions)**		
ASK TO BE SHOWN THE LOCATION IN THE FACILITY WHERE HIV PREVENTION, TREATMENT, AND CARE SERVICES ARE PROVIDED. FIND THE PERSON MOST KNOWLEDGEABLE ABOUT THESE SERVICES IN THE FACILITY, USUALLY THE "ART IN-CHARGE". INTRODUCE YOURSELF, EXPLAIN THE PURPOSE OF THE SURVEY AND ASK THE QUESTIONS IN SECTIONS 5 - 7.		
**Question**	**Options**	**Response**
44. Does this facility offer HIV counseling and	*1=Yes*<br>*0=No*	

testing services (HTS)?		
45. Does this facility offer Couples’ HIV counseling and testing (cHTC) services?	*1=Yes*<br>*0=No*	□
46. Does this facility offer partner notification services for HIV?	*1=Yes*<br>*0=No*	□
47. Do you have national HIV counseling and testing guidelines available in the facility today?	*1=Yes, observed*<br>*2=Yes, reported, not seen*<br>*0=No*	□
48. How many providers of HIV/AIDS testing services received any training in HIV testing and counseling services (HTS) in the last two years?		□□□
49. Is the HIV testing and counseling service room or area a private room/area with auditory and visual privacy?	*1= Privacy from people hearing you only*<br>*2= Privacy from people seeing you only*<br>*3=Privacy from people seeing or hearing you*<br>*0=No privacy*	a. Reported □ b. Observed □
50. Does the facility have HIV rapid test kits (with valid expiration date) in stock in this service site today?	*1=Yes, observed*<br>*2=Yes, reported, not seen*<br>*0=No*	□

**SECTION 9: HIV TREATMENT (3 Questions)**		
**Question**	**Options**	**Response**
51. Does this facility offer HIV/AIDS antiretroviral prescription or antiretroviral treatment follow-up services?	*1=Yes*<br>*0=No*	□
52. Does this facility provide treatment follow-up services for persons on ART, including providing community-based services?	*1=Yes*<br>*0=No*	□
53. Do you have National guidelines for the clinical	*1=Yes, observed*<br>*2=Yes, reported, not seen*<br>*0=No*	□

management of HIV/AIDS available in this facility today?		

SECTION 10: HIV CARE AND SUPPORT (14 Questions)		
**Question**	**Options**	**Response**
54. Does this facility offer HIV & AIDS care and support services, including treatment of opportunistic infections and provisions of palliative care?	*1=Yes*<br>*0=No*	□
***[Please read:** "Please tell me if this facility provides the following services for HIV/AIDS clients."]*		
55. Prescribe treatment for any opportunistic infections or symptoms related to HIV/AIDS?	*1=Yes*<br>*0=No*	□
56. Prescribe or provide therapeutic feeding or protein supplementation (such as F100, Chiponde or soya-fortified porridge)?	*1=Yes*<br>*0=No*	□
57. Treatment and care for paediatric HIV/AIDS patients?	*1=Yes*<br>*0=No*	□
58. Prescribe or provide preventative treatment for TB (INH + Pyridoxine)?	*1=Yes*<br>*0=No*	□
59. Primary preventative treatment for opportunistic infections, such as cotrimoxazole preventative treatment (CPT)?	*1=Yes*<br>*0=No*	□
60. Family planning for HIV/AIDS clients?	*1=Yes*<br>*0=No*	□
61. Provide condoms for preventing further transmission of HIV?	*1=Yes*<br>*0=No*	□
**Question**	**Options**	**Response**
62. Have any providers of HIV treatment, care and support services received any training in the clinical management of HIV/AIDS in the last two years?	*1=Yes*<br>*0=No*	□

Page 9 of 21

Study Site: □□□-□□

Date Completed □□/□□□/□□□□

Question	Options	Response
63. Number of total (adults and children) clients alive and on ART during the last reporting quarter?	*Specify # of people, disaggregated by sex (if possible)*	a. Reported:<br>Male Female
64. Number of HIV-positive reproductive age women alive and on ART during the last reporting quarter?	*Specify # of people, disaggregated by sex (if possible)*	a. Reported:<br>Male Female
65. Number of HIV-positive pregnant women newly starting ART during the last reporting quarter?	*Specify # of people, disaggregated by sex (if possible)*	a. Reported:<br>Male Female
66. Number of children 24 months of age and younger alive and on ART during the last reporting quarter?	*Specify # of people, disaggregated by sex (if possible)*	a. Reported:<br>Male Female
67. Number of infants newly registered into the 2-month birth cohort for HIV-exposure child follow-up during the last reporting quarter?	*Specify # of people, disaggregated by sex (if possible)*	a. Reported:<br>Male Female

SECTION 11: HIV DIAGNOSTICS (10 Questions)		
ASK TO BE SHOWN THE LOCATION IN THE FACILITY WHERE HIV DIAGNOSTIC SERVICES ARE PROVIDED. FIND THE PERSON MOST KNOWLEDGEABLE ABOUT THESE SERVICES IN THE FACILITY, USUALLY THE "LABORATORY IN-CHARGE". INTRODUCE YOURSELF, EXPLAIN THE PURPOSE OF THE SURVEY AND ASK THE FOLLOWING QUESTIONS.		
**Question**	**Options**	**Response**
68. Does this facility offer the following testing services **on-site**?	*1=Yes*<br>*0=No*	a. HIV-1/2 Rapid Testing (e.g. Determine and Unigold) □<br>b. Dried Blood Spot (DBS) collection for DNA PCR Testing □<br>c. Point-of-Care DNA PCR/ EID Testing (e.g. Alere q Detect or Xpert)

Completed By: □□ Verified By: □ Entered By: □□ Date Entered: □□/□□□/□□□□

		□
69. Is an HIV rapid test kit available or not available today?	*1=Yes, observed at least one valid (i.e. not expired)*<br>*2=Yes, observed available but no-valid (i.e. expired)*<br>*3=Reported available but not observed*<br>*4=Not available today*<br>*0=Never available*	□
70. Is filter paper for collecting DBS available or not available today?	*1=Yes, observed at least one pack valid (i.e. not expired)*<br>*2=Yes, observed available but pack non-valid (i.e. expired)*<br>*3=Reported available but not observed*<br>*4=Not available today*<br>*0=Never available*	□
71. Are POC EID testing cartridges available or not available today?	*1=Yes, observed at least one test valid (i.e. not expired)*<br>*2=Yes, observed available but test non-valid (i.e. expired)*<br>*3=Reported available but not observed*<br>*4=Not available today*<br>*0=Never available*	□
72. Has there been a stock out of HIV rapid test kits in the past 4 weeks?<br>***If 0=No, then Skip to question 74***	*1=Yes*<br>*0=No*	□
73. How many days of HIV test kit stock out has there been in the past 4 weeks?	*1=Less than 7 days*<br>*2=7 to 14 days*<br>*3=More than 14 days*	□
74. Has there been a stock out of DBS filter paper packs or POC EID testing cartridges in the past 4 weeks?<br>***If 0=No, then Skip to question 76***	*1=Yes*<br>*0=No*	□
75. How many days of DBS pack/ POC EID test cartridge stock out has there	*1=Less than 7 days*<br>*2=7 to 14 days*<br>*3=More than 14*	□

been in the past 4 weeks?		*days*	
76. Does this facility conduct HIV Viral Load testing either onsite or offsite?		*1=Yes, on-site*<br>*2=Yes, off-site*<br>*0=No, the test is not offered through this facility*	☐
Please Read: Please tell me if the following HIV testing equipment items and reagents are available and functional today.		**Available**	**Functional**
77. POC DNA PCR testing platform (e.g. Alere q Detect, Xpert, etc.)	*1=Observed*<br>*2=Reported, not seen*<br>*0=Not available/ Not functional*	☐	☐

**SECTION 12: SUPPLY CHAIN (9 Questions)**		
ASK TO BE SHOWN THE LOCATION IN THE FACILITY WHERE ARVS AND OTHER HEALTH COMMODITIES ARE STORED, USUALLY THE ART CLINIC OR PHARMACY. FIND THE PERSON MOST KNOWLEDGEABLE ABOUT THESE SERVICES IN THE FACILITY, USUALLY THE "PHARMACIST IN-CHARGE". INTRODUCE YOURSELF, EXPLAIN THE PURPOSE OF THE SURVEY AND ASK THE FOLLOWING QUESTIONS.		
**Question**	**Options**	**Response**
***[Please read:** Are the following ARVs available today in this facility?]*		
78. Adult fixed-dose combination 'Atripla' (TDF + 3TC/FTC + EFV)	*1=Yes, observed at least one pack valid (i.e. not expired)*<br>*2=Yes, observed available but pack non-valid (i.e. expired)*<br>*3=Reported available but not seen*<br>*4=Not available today*<br>*0=Never available*	☐
79. Paediatric formulation Nevirapine syrup	*1=Yes, observed at least one pack valid (i.e. not expired)*<br>*2=Yes, observed available but pack non-valid (i.e. expired)*<br>*3=Reported available but not seen*<br>*4=Not available today*<br>*0=Never available*	☐
80. Paediatric fixed-dose combination AZT + 3TC + NVP	*1=Yes, observed at least one pack valid (i.e. not expired)*<br>*2=Yes, observed available but pack non-valid (i.e. expired)*<br>*3=Reported available but not seen*<br>*4=Not available today*<br>*0=Never available*	☐
81. Paediatric formulation Lopinavir/ritonavir	*1=Yes, observed at least one pack valid (i.e. not expired)*<br>*2=Yes, observed available but pack non-valid (i.e. expired)*<br>*3=Reported available but not seen*<br>*4=Not available today*<br>*0=Never available*	☐

82. During the past 6 months, how often did you receive the amount of ARVs that you ordered, or you were supposed to routinely receive?	*1=Always*<br>*2=Often*<br>*3=Almost never*<br>*8=Declined to respond*<br>*9=Don't know*	□
83. Who provides your HIV pharmaceutical supplies (e.g. ARVs and CPT)? *(Tick all that apply)*	□ *National medical stores*<br>□ *District/ Province medical stores*<br>□ *NGO/Donors*<br>□ *Private sources*<br>□ *Other (specify): _______________*	
84. Who provides your HIV rapid test kits? *(Tick all that apply)*	□ *National medical stores*<br>□ *District/ Province medical stores*<br>□ *NGO/Donors*<br>□ *Private sources*<br>□ *Other (specify): _______________*	
85. Who provides your HIV laboratory and diagnostic supplies (apart from HIV rapid test kits)? By this I mean who supplies your facility with DBS filter paper, POC EID test cartridges, etc.? *(Tick all that apply)*	□ *National medical stores*<br>□ *District/ Province medical stores*<br>□ *NGO/Donors*<br>□ *Private sources*<br>□ *Other (specify): _______________*	

***[Please Read:** "Thank you very much for your time and your participation in this interview. Do you have any questions for me?"]*

Part II: CFL Programme Factors

Instructions: This part of the survey is to be completed through direct observation and through interviews with the CFL Site-level Programme Supervisor or Designee.

SECTION 1: BASIC INFORMATION (6 Questions)	
PLEASE FIND THE CFL SITE-LEVEL SUPERVISOR OR EQUIVALENT, INTRODUCE YOURSELF, EXPLAIN THE PURPOSE OF THE SURVEY AND ASK THE QUESTIONS IN SECTIONS 1 – 5.	
1. Date of programme/ site visit:	*dd/MMM/yyyy* □□/□□□/□□□□

Study Site: □□□-□□

Date Completed □□/□□□/□□□□

2. Day of the week of programme/ site visit:		□□□
3. Interviewer / Study Staff:	*Name (printed):* ______________________	
4. Study Site:	*Refer to Study Site Code List*	□□□-□□
5. CFL Programme:	*0=Mother2Mothers*<br>*1=Partners In Health*<br>*2=Tingathe*<br>*3=UNC*<br>*4=EGPAF*<br>*5=Dignitas*<br>*6=EQUIP*<br>*7=Other (specify):* ___________	□
6. District:	*Please print:* ______________________	

**SECTION 2: PERSONNEL (19 Questions)**			
[Please Read: *"I have a few questions about your programme. Please tell me which types of staff/ workers/ volunteers you engage with, and the total number currently assigned to, employed by, or seconded to your programme to support PMTCT/EID-related service delivery at site-level. Please count every staff member/ worker/ volunteer only once."]*			
7. CFL Programme/ Provider Type(s):	*1=Mentor Mother*<br>*2=Community Health Worker*<br>*3=Expert Client/ Mother*<br>*4=More than 1 type (specify)*<br>*7=Other (specify)*	*(specify if more than 1 or other):* ______________________	□
FOR EACH PROVIDER TYPE INDICATED IN QUESTION #7, NOTE BELOW THE NUMBER OF PROVIDERS REPORTED BY THE RESPONDENT AND THE NUMBER OF PROVIDERS OBSERVED AT THE SITE. USUALLY THIS WILL BE JUST ONE PROVIDER TYPE PER CFL PROGRAMME. IF THERE IS MORE THAN ONE PROGRAMME AT THE SAME SITE, COMPLETE A DIFFERENT PART II OF THIS SURVEY FOR EACH CFL PROGRAMME. IF NON-APPLICABLE, TICK "N/A" AND GO TO THE NEXT QUESTION.			
**Provider Type**		**Reported**	**Observed**
8. Mentor Mother:	N/A □	a. □□□	b. □□□
9. Community Health Worker:	N/A □	a. □□□	b. □□□

Completed By: □□ Verified By: □ Entered By: □□ Date Entered: □□/□□□/□□□□

10. Expert Client/ Mother:	N/A □	a. □□□	b. □□□
11. Other CFL Provider, *specify*: ________________	N/A □	a. □□□	b. □□□
12. Field Supervisor Type(s):		*1=Facility-based supervisor*<br>*2=Community-based supervisor*<br>*3=Traveling supervisor (splits supervision activities between facilities and communities)*<br>*4=More than 1 supervisor type (specify)*<br>*7=Other (specify)*<br>*9=No field supervisors reported*	□<br>*(specify if more than 1 or other):* ________
**Supervisor Type**		**Reported**	**Observed**
13. Facility-based Supervisors:	N/A □	a. □□□	b. □□□
14. Community-based Supervisors:	N/A □	a. □□□	b. □□□
15. Traveling Supervisors:	N/A □	a. □□□	b. □□□
16. ***TOTAL*** Field Supervisors:	N/A □	a. □□□	b. □□□
17. CFL Programme/ Provider Minimum Educational Requirement	*1=None*<br>*2=Primary School*<br>*3=Malawi School Certificate of Education (MSCE)*<br>*4=Diploma*<br>*5=Bachelor's degree*<br>*7=Other (specify)*	□<br>*(specify if Other):* ________	
18. Are CFL lay/peer providers in your programme employed by your organization? That is do they receive a salary at regular intervals?<br>***If No, Skip to Question 21***	*1=Yes*<br>*0=No*	□	
19. If CFL lay/peer providers in your programme are employees or paid workers, what is their monthly salary, on average in MWK?<br>***Skip to Question 21***		□□□,□□□	

20. Are CFL lay/peer providers in your programme considered by your programme to be volunteers?	*1=Yes*<br>*0=No*	☐
21. Does your program provide a sitting allowance to CFL lay/peer providers for trainings and other meetings?<br>***If No, Skip to Question 23***	*1=Yes*<br>*0=No*	☐
22. If a sitting/ training allowance is provided, how much is provided, on average, per quarter (in MWK)?		☐☐☐,☐☐☐
23. Please tell me if your program provides the following types of non-salary remuneration to CFL lay/peer providers in your programme?	*1=Yes*<br>*0=No*	a. Uniform/ clothing (e.g. shirts or gumboots) ☐<br>b. Mobile phone airtime ☐<br>c. Agricultural inputs such as fertilizer ☐<br>d. Food package/ nutritional supplement ☐
24. Does your program provide another type of non-salary remuneration I haven't mentioned?	*1=Yes*<br>*0=No*	☐<br>*(please specify):* ______
25. On average, how many clients (counting mothers and their infants separately), does an individual CFL provider in your programme look after?		☐☐☐

SECTION 3: TRAINING & SUPERVISION (9 Questions)		
**Question**	**Options**	**Response**
26. Do lay/ peer providers in your programme receive formal introductory training before starting activities?<br>***If No, Skip to Question 29***	*1=Yes*<br>*0=No*	☐

27. If so, how long is the introductory training?		□□ days
28. Is a review of relevant material from Malawi National Guidelines (HIV Testing Services / Clinical Management of HIV/AIDS/STIs) included in your training?	*1=Yes*<br>*0=No*	□
29. Do CFL lay/peer providers in your programme receive formal training in HIV testing services (HTS)?	*1=Yes*<br>*0=No*	□
30. Do CFL lay/peer providers in your programme receive refresher training?<br>***If No, Skip to Question 32***	*1=Yes*<br>*0=No*	□
31. If so, with what frequency do refresher trainings occur?	*0=Daily*<br>*1=Weekly*<br>*2=Fortnightly*<br>*3=Monthly*<br>*4=Quarterly*<br>*5=Bi-Annually*<br>*7=Other (specify)*<br>*8=Declined to respond*<br>*9=Don't know*	□<br>*(specify if other):* ______
32. Are CFL lay/peer providers in your programme provided with paper-based or digital/mHealth job aids?	*0=No job aids are provided*<br>*1=Yes, paper-based*<br>*2=Yes, digital/ mHealth*	□
33. Do CFL lay/peer providers in your programme receive supportive supervision?<br>***If No, Skip to Question 35***	*1=Yes*<br>*0=No*	□
34. If so, with what frequency do CFL lay/peer providers in your programme receive supportive supervision?	*0=Daily*<br>*1=Weekly*<br>*2=Fortnightly*<br>*3=Monthly*<br>*4=Quarterly*<br>*5=Bi-Annually*<br>*6=Annually*<br>*7=Other (specify)*<br>*8=Declined to respond*<br>*9=Don't know*	□<br>*(specify if other):* ______

SECTION 4: ACTIVITIES (17 Questions)			
[Please Read: *"This next section will focus on questions related to CFL provider activities."]*			
**Question**	**Options**	**Reported**	**Observed**
**Psychosocial Support**			
35. Do CFL lay/peer providers in your programme facilitate patient support group meetings? ***If No, Skip to Question 38***	*1=Yes*<br>*0=No*	a. □	b. □
36. On average, how many clients are there per support group?	*Specify # of clients*	a. □□□	b. □□□
37. On average, how frequently do the support groups meet?	*0=Daily*<br>*1=Weekly*<br>*2=Fortnightly*<br>*3=Monthly*<br>*4=Quarterly*<br>*5=Bi-Annually*<br>*6=Annually*<br>*7=Other (specify)*<br>*8=Declined to respond*<br>*9=Don't know*	a. □<br>*(specify if other):* ________	b. □<br>*(specify if other):* ________
38. Do CFL lay/peer providers in your programme conduct *routine* home visits (i.e. NOT for defaulter tracing/ returning clients to care)? ***If No, Skip to Question 41***	*1=Yes*<br>*0=No*	a. □	b. □
39. On average, how many *routine* home visits does a CFL lay/peer provider in your programme offer each client per quarter?	*Specify average # of routine home visits per client per quarter*	a. □□	
40. On average, how many *routine* home visits does a CFL lay/peer provider in your programme offer each client during their first month on ART?	*Specify average # of routine home visits per client during their first month on ART*	a. □□	
41. Do CFL lay/peer providers in your programme phone clients when they miss an appointment?	*1=Yes*<br>*0=No*	a. □	b. □
42. Do CFL lay/peer providers in your programme physically visit clients in the community or at their home if they miss an appointment?	*1=Yes*<br>*0=No*	a. □	b. □
43. Do CFL lay/peer providers in your programme initiate defaulter tracing activities if the mother	*2=Either*<br>*1=Infant*<br>*0=Mother*	a. □	

misses an appointment, the infant misses an appointment, or either one misses an appointment?			
44. How long after a missed appointment does it take for a CFL lay/peer provider to initiate tracing activities (e.g. phone call or home visit)?	*0=Same Day<br>1=Next Day<br>2=Between 2 – 7 days<br>3=Between 8 – 14 days<br>4=Between 15 – 30 days<br>5=After 1 month<br>6=After 3 months<br>7=Other (specify)*	a. □<br>*(specify if other):* ____	
45. On average, how many “defaulting” clients does a CFL lay/peer provider in your programme physically trace/ visit in the community each month?	*Specify average # of “defaulting” clients traced/ visited in the community per CFL provider per month*	a. □□	
46. Do CFL lay/peer providers in your programme accompany clients to their clinic visits?<br>***If No, Skip to Question 48***	*1=Yes<br>0=No*	a. □	b. □
47. If so, how often do your CFL lay/peer providers accompany clients to their clinic visits?	*0=For every visit<br>1=For their first few visits after starting ART<br>2=Only for their first visit after being successfully traced/ returned to care<br>3=Other (specify)*	a. □<br>*(specify if other):* ____	
**Counseling**			
48. Do CFL lay/peer providers in your programme provide the following counseling services to clients?	*1=Yes, observed<br>2=Yes, reported, not seen<br>0=No*	a. HIV disclosure counseling to clients □<br>b. One-on-one psychosocial counseling □<br>c. Adherence counseling □<br>d. Nutritional counseling □<br>e. Breastfeeding/ infant feeding counseling □	
**HIV Testing Services**			
49. Do CFL lay/peer providers in your programme provide the	*1=Yes, observed<br>2=Yes,*		

Completed By: □□ Verified By: □ Entered By: □□ Date Entered: □□/□□□/□□□□

following HIV testing services to adults?	*reported, not seen*<br>*0=No*	a. HIV testing & counseling (HTC) for mothers ☐<br>b. Couples' HTC ☐<br>c. Dried Blood Spot (DBS) Collection for EID ☐<br>d. DBS Collection for Viral Load testing ☐<br>e. DNA PCR testing using a POC device ☐
**Community Mobilization & Health Education**		
50. Do CFL lay/peer providers in your programme conduct the following?	*1=Yes, observed*<br>*2=Yes, reported, not seen*<br>*0=No*	a. Health talks at the facility ☐<br>b. Health talks in the community ☐<br>c. Other community mobilization/ education ☐<br>*(specify):* ________
51. Do CFL lay/peer providers in your programme carry out other activities not mentioned previous?	*1=Yes*<br>*0=No*	a. ☐ *(specify):* ________ ________<br>b. ☐ *(specify):* ________ ________

SECTION 5: OVERLAP & COORDINATION (4 Questions)			
***[Please Read:** "This next section will focus on how CFL programmes/ projects coordinate in this catchment area."]*			
**Question**	**Options**	**Reported**	**Observed**
52. Are there other partners or projects in this catchment area currently supporting community-facility linkage for PMTCT/ EID through the engagement of lay/ peer workers or volunteers?	*1=Yes, observed*<br>*2=Yes, reported, not seen*<br>*0=No*	a. ☐	b. ☐

If No, Skip to Question 54			
53. If so, do you meet with or otherwise coordinate CFL services with the other partners or projects operating in this catchment area?	*1=Yes, observed*<br>*2=Yes, reported, not seen*<br>*0=No*	a. □	b. □
54. Do clients in your programme receive support from other types of CFL lay/ peer providers over the course of the antenatal and postnatal periods (e.g. a mentor mother during pregnancy and a CHW after delivery)?<br>***If No, STOP***	*1=Yes*<br>*0=No*<br>*8=Declined to respond*<br>*9=Don't know*	a. □	
55. Please briefly describe the services that your clients receive from other types of CFL lay/ peer providers in this catchment area?		*a.* ____<br>*b.* ____<br>*c.* ____<br>*d.* ____<br>*e.* ____	*a.* ____<br>*b.* ____<br>*c.* ____<br>*d.* ____<br>*e.* ____

[Please Read: *"Thank you very much for your time and your participation in this interview. Do you have any questions for me?"*]

Completed By: □□ Verified By: □ Entered By: □□ Date Entered: □□/□□□/□□□□
