# Supplementary material for: Community-facility linkage models and maternal and infant health outcomes in Malawi’s PMTCT/ART program: A cohort study
Source: PLoS Med. 2021 Sep 17;18(9):e1003780. doi: 10.1371/journal.pmed.1003780 (PMC8516224; doi:10.1371/journal.pmed.1003780)
Supplement: S1 Table — (DOCX) [file pmed.1003780.s004.docx]

| **Characteristic/ Activity^a^** | **Expert Clients** | **Community Health Workers** | **Mentor Mothers** | **No CFL model/ “Traditional” SOC** |
| --- | --- | --- | --- | --- |
| *Focus Population* | All people living with HIV, with a focus on women and children | Pregnant and breastfeeding women living with HIV and infants exposed to HIV | Pregnant and breastfeeding women living with HIV, infants exposed to HIV, and children living with HIV | All people within their catchment areas |
| *Work Setting* | Health facility & communities | Health facility & communities | Mostly Health facility  (Some sites have community-based Mentor Mothers) | Health facility & communities  (Ministry of Health Health Surveillance Assistants often work out of health posts based in the community) |
| *CFL Provider Mobility & Distance Travelled* | No restrictions on distances travelled | No restrictions on distances travelled; some have bicycles to improve their reach & efficiency | Facility-based mentor mothers are restricted to a 5 kilometer travel radius from their assigned facility | No restrictions on distances travelled |
| *Routine Home Visits* | ✔- | ✔ | ✔ | ✔ |
| *Routine Home Visit Frequency^b^* | 4.7 per quarter | 3.4 per quarter | 3 per quarter | 4 per quarter |
| *Tracing of Clients Lost to Care* | ✔ | ✔ | ✔ | ✔ |
| *Tracer Ratio^c^* | 1 Expert Clients per 10 Clients | 1 CHW per 4 Clients | 1 Mentor Mother per 5 Clients | 1 Health Surveillance Assistant per 4 Clients |
| *Provide HTS services* | ✔**-** | ✔ | ✔**-** | ✔ |
| *Conduct EID testing* | ⦸ | ✔ | ⦸ | ✔ |
| *Disclosure counseling* | ✔**-** | ✔ | ✔ | ✔ |

^a^For this table, a “✔**-**” refers to fewer than 100% of respondents reporting the characteristic or activity of interest for the CFL model, while “✔” refers to 100% of respondents reporting the characteristic or activity of interest for the CFL model; and “⦸” refers to 0% of respondents reporting the characteristic or activity of interest for the CFL model.

^b^Calculated as the average number of home visits a CFL provider offers a client each quarter as reported by CFL program manager.

^c^Calculated as average number of clients lost to care that one CFL provider traces within 1 month as reported by CFL program manager.

CFL, Community Facility Linkage; SOC, Standard of Care; HTS, HIV Testing Services; EID, Early Infant Diagnosis of HIV
