# Supplementary material for: Community-facility linkage models and maternal and infant health outcomes in Malawi’s PMTCT/ART program: A cohort study
Source: PLoS Med. 2021 Sep 17;18(9):e1003780. doi: 10.1371/journal.pmed.1003780 (PMC8516224; doi:10.1371/journal.pmed.1003780)
Supplement: S2 Table — (DOCX) [file pmed.1003780.s005.docx]

| **Site Number** | **Number of MIPs potentially eligible for Study Cohort after initial medical record review** | **Site-level Sampling Fraction for Field Survey** | **Number Sampled** | **Number Recruited, Contacted, and Completing Field Survey** |
| --- | --- | --- | --- | --- |
| LL-01 | 75 | 100% | 75 | 32 |
| LL-02 | 324 | 100%* | 243 | 47 |
| LL-03 | 15 | 100% | 15 | 9 |
| LL-04 | 28 | 100% | 28 | 11 |
| LL-05 | 76 | 100% | 76 | 26 |
| LL-06 | 60 | 100% | 60 | 16 |
| LL-07 | 58 | 100% | 58 | 19 |
| LL-08 | 31 | 100% | 31 | 10 |
| LL-09 | 6 | 100% | 6 | 3 |
| LL-10 | 122 | 100% | 122 | 31 |
| LL-11 | 307 | 100% | 307 | 38 |
| MZ-01 | 196 | 100% | 196 | 46 |
| MZ-02 | 54 | 100% | 54 | 31 |
| MZ-03 | 41 | 100% | 41 | 19 |
| MZ-04 | 20 | 100% | 20 | 9 |
| MZ-05 | 87 | 100% | 87 | 32 |
| MZ-06 | 38 | 100% | 38 | 23 |
| SA-01 | 36 | 100% | 36 | 17 |
| SA-02 | 37 | 100% | 37 | 23 |
| SA-03 | 228 | 100% | 228 | 114 |
| SA-04 | 52 | 100% | 52 | 41 |
| SA-05 | 61 | 100% | 61 | 38 |
| ZA-01 | 99 | 100% | 99 | 33 |
| ZA-02 | 74 | 100% | 74 | 35 |
| ZA-03 | 84 | 100% | 84 | 29 |
| ZA-04 | 127 | 75% | 93 | 29 |
| ZA-05 | 122 | 75% | 92 | 28 |
| ZA-06 | 38 | 100% | 38 | 21 |
| ZA-07 | 62 | 100% | 62 | 15 |
| ZA-08 | 31 | 100% | 31 | 7 |
| **Total** | **2,589** | **94%** | **2,444** | **832** |

*Initially sampled at 75%, but then changed to 100% because of low enrollment into field survey

MIPs, mother-infant pairs; LL, Lilongwe; MZ, Mzimba North/ South; SA, Salima; ZA, Zomba.
