# Supplementary material for: Community-facility linkage models and maternal and infant health outcomes in Malawi’s PMTCT/ART program: A cohort study
Source: PLoS Med. 2021 Sep 17;18(9):e1003780. doi: 10.1371/journal.pmed.1003780 (PMC8516224; doi:10.1371/journal.pmed.1003780)
Supplement: S3 Table — (DOCX) [file pmed.1003780.s006.docx]

| **Study Site Number** | **Distribution of CFL model exposure by site, as reported by field survey participants with complete medical records (N=817)** | | | | | **CFL model selected for imputation for all mother-infant pairs at the site** |
| --- | --- | --- | --- | --- | --- | --- |
|  | ***Expert Clients (%)*** | ***Community Health Workers (%)*** | ***Mentor Mothers (%)*** | ***≥2 CFL Models (%)*** | ***No CFL Model (%)*** |  |
| LL-01 | 0% | 19% | 22% | 58% | 0% | ≥2 CFL Models |
| LL-02 | 29% | 13% | 6% | 0% | 52% | No CFL Model |
| LL-03 | 88% | 0% | 0% | 0% | 13% | Expert Clients |
| LL-04 | 24% | 0% | 24% | 35% | 18% | ≥2 CFL Models |
| LL-05 | 0% | 0% | 61% | 39% | 0% | Mentor Mothers |
| LL-06 | 0% | 64% | 6% | 21% | 9% | CHW |
| LL-07 | 0% | 97% | 0% | 0% | 3% | CHW |
| LL-08 | 0% | 100% | 0% | 0% | 0% | CHW |
| LL-09 | 100% | 0% | 0% | 0% | 0% | Expert Clients |
| LL-10 | 17% | 0% | 60% | 20% | 3% | Mentor Mothers |
| LL-11 | 84% | 0% | 0% | 0% | 16% | Expert Clients |
| MZ-01 | 0% | 98% | 0% | 0% | 2% | CHW |
| MZ-02 | 19% | 0% | 35% | 31% | 15% | Mentor Mothers |
| MZ-03 | 27% | 0% | 50% | 13% | 10% | Mentor Mothers |
| MZ-04 | 33% | 0% | 67% | 0% | 0% | Mentor Mothers |
| MZ-05 | 45% | 2% | 11% | 14% | 27% | Expert Clients |
| MZ-06 | 100% | 0% | 0% | 0% | 0% | Expert Clients |
| SA-01 | 97% | 0% | 0% | 0% | 3% | Expert Clients |
| SA-02 | 0% | 29% | 0% | 0% | 71% | No CFL Model |
| SA-03 | 0% | 9% | 36% | 27% | 27% | Mentor Mothers |
| SA-04 | 0% | 0% | 0% | 0% | 100% | No CFL Model |
| SA-05 | 100% | 0% | 0% | 0% | 0% | Expert Clients |
| ZA-01 | 44% | 0% | 0% | 11% | 44% | Expert Clients |
| ZA-02 | 93% | 0% | 0% | 0% | 7% | Expert Clients |
| ZA-03 | 100% | 0% | 0% | 0% | 0% | Expert Clients |
| ZA-04 | 0% | 91% | 0% | 0% | 9% | CHW |
| ZA-05 | 0% | 88% | 0% | 0% | 12% | CHW |
| ZA-06 | 67% | 0% | 0% | 0% | 33% | Expert Clients |
| ZA-07 | 3% | 0% | 97% | 0% | 0% | Mentor Mothers |
| ZA-08 | 81% | 0% | 0% | 10% | 10% | Expert Clients |

CFL, Community Facility Linkage; CHW, Community Health Worker; LL, Lilongwe; MZ, Mzimba North/ South; SA, Salima; ZA, Zomba.
