# Supplementary material for: Community-facility linkage models and maternal and infant health outcomes in Malawi’s PMTCT/ART program: A cohort study
Source: PLoS Med. 2021 Sep 17;18(9):e1003780. doi: 10.1371/journal.pmed.1003780 (PMC8516224; doi:10.1371/journal.pmed.1003780)
Supplement: S4 Table — (DOCX) [file pmed.1003780.s007.docx]

| **Characteristics** | | **Study Cohort**  ***N* = 2,049**  **n, %** | | **Field Survey Participants**  *(i.e., sample of study cohort)*  ***N* = 817**  **n, %** | |
| --- | --- | --- | --- | --- | --- |
| Age, years |  |  |  |  |  |
|  | <18 | 83 | 4.6 | 33 | 4.5 |
|  | 18–24 | 698 | 39.0 | 265 | 36.0 |
|  | 25–29 | 450 | 25.2 | 182 | 24.7 |
|  | 30–34 | 379 | 21.2 | 173 | 23.5 |
|  | ≥35 | 178 | 10.0 | 83 | 11.3 |
|  | Missing^a^ | 261 |  | 81 |  |
| District |  |  |  |  |  |
|  | Lilongwe | 767 | 37.4 | 212 | 26.0 |
|  | Mzimba  (North plus South) | 400 | 19.5 | 163 | 20.0 |
|  | Salima | 353 | 17.2 | 241 | 29.5 |
|  | Zomba | 529 | 25.8 | 201 | 24.6 |
| Facility type/ level |  |  |  |  |  |
|  | Government Primary Health Centre | 1,255 | 61.3 | 452 | 55.3 |
|  | Government Rural/Community Hospital | 64 | 3.1 | 28 | 3.4 |
|  | Government District Hospital | 274 | 13.4 | 150 | 18.4 |
|  | Christian Health Association of Malawi (CHAM) Facility | 456 | 22.3 | 187 | 22.9 |
| WHO Stage at PMTCT/ ART enrolment |  |  |  |  |  |
|  | 1 | 1,897 | 99.8 | 767 | 99.6 |
|  | 2 | 4 | 0.2 | 3 | 0.4 |
|  | Missing^a^ | 148 |  | 47 |  |
| CFL Model Received^b^ |  |  |  |  |  |
|  | Expert Clients | 778 | 38.0 | 277 | 33.9 |
|  | Community Health Workers | 640 | 31.2 | 286 | 35.0 |
|  | Mentor Mothers | 345 | 16.8 | 154 | 18.8 |
|  | ≥2 CFL Models | 192 | 9.4 | 51 | 6.2 |
|  | No CFL Model | 94 | 4.6 | 49 | 6.0 |
|  | Missing^a^ |  |  |  |  |

^a^Missing data were not included in the denominator for calculation of percentages for characteristics of interest;

^b^Based on participant self-report, where available, and, where not available, based on single imputation derived from distribution of Field Survey and CFL Model Survey responses.

CFL, Community Facility Linkage; WHO, World Health Organization
