# Supplementary material for: Community-facility linkage models and maternal and infant health outcomes in Malawi’s PMTCT/ART program: A cohort study
Source: PLoS Med. 2021 Sep 17;18(9):e1003780. doi: 10.1371/journal.pmed.1003780 (PMC8516224; doi:10.1371/journal.pmed.1003780)
Supplement: S5 Table — (DOCX) [file pmed.1003780.s008.docx]

| **Last Known PMTCT program status at 18 months^a^** | **Study Cohort**  ***N*=1,549** | |
| --- | --- | --- |
|  | n | % |
| *Established HIV-infected* |  |  |
| In Care | 36 | 2.3 |
| Died | 1 | 0.1 |
| Defaulted | 2 | 0.1 |
| Transferred Out | 1 | 0.1 |
| *Sub-total* | *40* | *2.6* |
|  |  |  |
| *Established HIV-infected, Diagnosed by Study* |  |  |
| In Care | 2 | 0.1 |
| Newly Linked, missing follow-up card | 2 | 0.1 |
| *Sub-total* | *4* | *0.3* |
|  |  |  |
| *Established HIV-free* |  |  |
| Discharged | 17 | 1.1 |
| Alive, missing follow-up card but established HIV-free through Field Survey | 172 | 11.1 |
| *Sub-total* | *189* | *12.2* |
|  |  |  |
| *HIV-exposed, HIV status not established* |  |  |
| In Care | 1,054 | 68.0 |
| Died, documented in routine record | 9 | 0.6 |
| Died, ascertained by Field Survey | 10 | 0.7 |
| Defaulted | 213 | 13.8 |
| Transferred Out | 30 | 1.9 |
| *Sub-total* | *1,316* | *85.0%* |

^a^890 infants from the study cohort were administratively censored, i.e. not followed for the full period of 18 months. Of all infants who were administratively censored, the median duration of follow-up was 403 days (IQR 323-480).

PMTCT, prevention of mother-to-child transmission of HIV.
